# Accelerometer-Measured Physical Activity and Neuroimaging-Driven Brain Age

**DOI:** 10.34133/hds.0257

**Published:** 2025-05-02

**Authors:** Han Chen, Zhi Cao, Jing Zhang, Dun Li, Yaogang Wang, Chenjie Xu

**Affiliations:** ^1^ School of Public Health, Hangzhou Normal University, Hangzhou, China.; ^2^ Department of Psychiatry, Sir Run Run Shaw Hospital, Zhejiang University School of Medicine, Hangzhou, China.; ^3^ School of Integrative Medicine, Public Health Science and Engineering College, Tianjin University of Traditional Chinese Medicine, Tianjin, China.; ^4^ School of Public Health, Tianjin Medical University, Tianjin, China.; ^5^ National Institute of Health Data Science at Peking University, Peking University, Beijing, China.

## Abstract

**Background:** A neuroimaging-derived biomarker termed the brain age is considered to capture the degree and diversity in the aging process of the brain, serving as a robust indicator of overall brain health. The impact of different levels of physical activity (PA) intensities on brain age is still not fully understood. This study aimed to investigate the associations between accelerometer-measured PA and brain age. **Methods:** A total of 16,972 eligible participants with both valid *T*_1_-weighted neuroimaging and accelerometer data from the UK Biobank was included. Brain age was estimated using an ensemble learning approach called Light Gradient-Boosting Machine (LightGBM). Over 1,400 image-derived phenotypes (IDPs) were initially chosen to undergo data-driven feature selection for brain age prediction. A measure of accelerated brain aging, the brain age gap (BAG) can be derived by subtracting the chronological age from the estimated brain age. A positive BAG indicates accelerated brain aging. PA was measured over a 7-day period using wrist-worn accelerometers, and time spent on light-intensity PA (LPA), moderate-intensity PA (MPA), vigorous-intensity PA (VPA), and moderate- to vigorous-intensity PA (MVPA) was extracted. The generalized additive model was applied to examine the nonlinear association between PA and BAG after adjusting for potential confounders. **Results:** The brain age estimated by LightGBM achieved an appreciable performance (*r* = 0.81, mean absolute error [MAE] = 3.65), which was further improved by age bias correction (*r* = 0.90, MAE = 3.03). We found that LPA (*F* = 2.47, *P* = 0.04), MPA (*F* = 6.49, *P* < 1 × 10^−300^), VPA (*F* = 4.92, *P* = 2.58 × 10^−5^), and MVPA (*F* = 6.45, *P* < 1 × 10^−300^) exhibited an approximate U-shaped relationship with BAG, demonstrating that both insufficient and excessive PA levels adversely impact brain aging. Furthermore, mediation analysis suggested that BAG partially mediated the associations between PA and cognitive functions as well as brain-related disorders. **Conclusions:** Our study revealed a U-shaped association between accelerometer-measured PA and BAG, highlighting that advanced brain health may be attainable through engaging in moderate amounts of objectively measured PA irrespectively of intensities.

## Introduction

The process of aging affects each individual at different degrees and levels due to genetic and environmental effects, with the brain being particularly susceptible to its major consequences [1]. Structural brain properties undergo consistent change throughout the course of one’s life [2]. Thus, the changes in brain structure that occur with age can be utilized to estimate an individual’s structural brain age, regardless of their chronological age. Brain age is estimated by training machine learning algorithms to predict age from structural magnetic resonance imaging (MRI) data collected in large samples of individuals across a broad age range [3]. The difference between predicted brain age and chronological age is termed the brain age gap (BAG) and is usually interpreted as a measure of accelerated aging of the brain. The application of brain age prediction models has been spreading rapidly in recent years to investigate the influence of brain age on various health conditions [4], such as in dementia [1], depression [5], schizophrenia [6], and mortality [7]. However, investigations into the correlation to BAG by biological, environmental, and lifestyle factors linked to these health conditions remain relatively limited, with only a few factors, such as infections [8], smoking [9], and education level [10] being reported. Identification of modifiable risk factors for brain age is particularly crucial for establishing targeted preventive measures and promoting brain health.

One potential modifiable factor for brain health is physical activity (PA). Accumulating studies have reported the association between PA and brain structures [11–13], such as gray matter structures [14] and hippocampal volumes [13]. However, prior evidence has been largely derived from studies using subjective and self-reported data on PA [12,13,15], which are prone to recall biases, particularly in distinguishing level and intensities of activity. Moreover, brain age is increasingly recognized as a promising measure representing overall brain health [3]. The available evidence on the relationship between objectively measured PA and brain age is notably deficient. An accelerometer is a promising wearable device for objectively recording the density and duration of all PA under free-living conditions for a long time (usually several days) [16,17]. To our knowledge, only one study has examined the association between accelerometer-measured PA and brain age in a sample of 122 healthy older adults, which narrowly focused on walking speed, postural control, and hand-grip strength by an accelerometer [18]. Therefore, understanding the association between objectively measured PA and brain age carries important implications for advancing public health and optimizing treatment strategies.

**Fig. 1.**
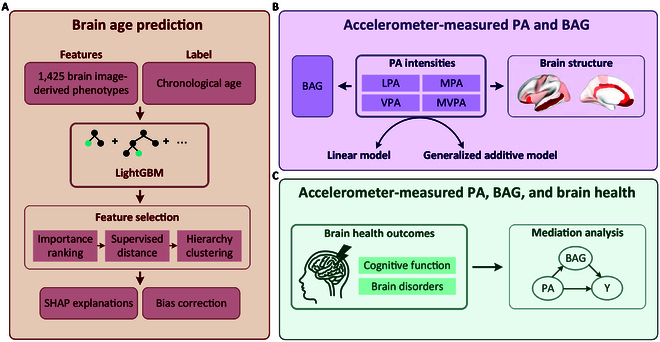
Workflow of the study. (A) The brain age prediction model is constructed by leveraging LightGBM algorithm training on 1,425 image-derived phenotypes (IDPs) from *T*_1_-weighted brain MRI and chronological age. Features initially undergo tree-based feature importance ranking, where the top 50 important features are picked out. Next, supervised distance between each feature is calculated and then underwent hierarchy clustering to identify redundant feature groups. After removing redundancy, we visually interpret the final selected subset of features using the SHAP technique. To deal with bias, predicted brain age was corrected by the linear method. (B) We first investigate correlations between objectively measured PA and BAG using both nonlinear and linear models. Next, to gain insight into PA and brain structures, we investigate correlations between PA and 1,425 IDPs using both nonlinear and linear models. (C) To verify whether PA and brain health was mediated by BAG, we conducted mediation analysis. Cognitive function and brain disorders were selected as brain health outcomes of interest. LightGBM, Light Gradient-Boosting Machine; SHAP, SHapley Additive exPlanations; PA, physical activity; LPA, light-intensity PA; MPA, moderate-intensity PA; VPA, vigorous-intensity PA; MVPA, moderate- to vigorous-intensity PA; BAG, brain age gap.

To address these gaps, this study aimed to investigate the associations of the amounts of light-intensity PA (LPA), moderate-intensity PA (MPA), and vigorous-intensity PA (VPA) measured by accelerometers with brain age (Fig. 1). Using data from the UK Biobank (UKB), the largest neuroimaging databases, we constructed a brain age prediction model by leveraging an ensemble learning approach termed Light Gradient-Boosting Machine (LightGBM), which offers advantages including higher prediction accuracy, efficient handling of high-dimensional data, and robust performance with complex neuroimaging features [19]. More importantly, the UKB holds the record for being the largest database to measure PA by an accelerometer; utilizing such devices to capture PA data may uncover the true magnitude of the association with brain age and facilitate personalized brain health in the context of the rapid growth of commercialized wearable devices equipped with accelerometers [17].

## Methods

### Study design and participants

The current study examined the data obtained from the UKB. Between 2006 March 1 and 2010 October 31, the UKB recruited over 500,000 adults (37 to 73 years old) from the general population [20]. It was approved by the North West Multicenter Research Ethnical Committee (reference numbers: 16/NW/0274 and 21/NW/0157) and all participants gave written informed consent at study entry. Participants attended 1 of 22 assessment centers across England, Scotland, and Wales, where they completed nurse-led electronic questionnaires, physical examination, and biological sample collections [20]. An imaging subsample was incorporated into the UKB in 2014 that strove to include brain, heart, and body MRI imaging from 100,000 participants [21]. In this study, we included 45,719 participants who possessed valid data on *T*_1_-weighted neuroimaging to calculate brain age. The UKB collected 7 days of accelerometers from 103,660 participants [22], with 92,602 participants passing quality control (Supplementary Methods). We included 17,621 participants who have both brain imaging and accelerometer data available, and an additional 412 participants were excluded due to missing covariates. Considering the highly discrete nature of PA values, we excluded PA values outside the range of 4 standard deviations (SDs). Consequently, we finally included 16,972 eligible participants for the primary analysis (Fig. S1). Summary statistics were presented as median (interquartile range [IQR]) and percentage (%).

### Image acquisition and preprocessing

A 3-T, 32-channel coil Siemens Skyra scanner (Siemens Medical Solutions, Germany) was used to obtain the MRIs, with 1 × 1 × 1 mm resolution and a field of view of 208 × 256 × 256 matrix. The raw imaging data have been processed to create a set of image-derived phenotypes (IDPs) [21,23]. In addition, several cortical surface and subcortical structures were derived by FreeSurfer modelling with various parcellation methods [24]. The investigation incorporated all relevant *T*_1_ structural brain MRI atlas regions (Field ID: 190 to 197; 1101 to 1102), yielding a total of 1,425 distinct MRI regions encompassing measurements of volumes of subcortical structures, surface area of cortical, and gray matter cortical thickness.

### Brain age prediction

The selection of IDPs for constructing a brain age prediction model was refined through the implementation of a 2-step feature selection approach. To avoid data leakage and ensure robust feature selection, we performed all feature selection steps within the cross-validation framework. Specifically, the dataset was randomly partitioned into 10 folds. For each iteration, 9 folds were used as training data and the remaining fold as test data. Initially, 1,425 IDPs were chosen for evaluation using the sophisticated ensemble learning algorithm, LightGBM [19]. This algorithm was fine-tuned to accurately measure the importance of each feature by employing a tree-based feature importance ranking system. To adopt the optimal combination of hyperparameters and minimize mean absolute error (MAE), we employed the blended search strategy [25] during 10-fold cross-validation. For detailed information on the hyperparameter search space and the final hyperparameters, please refer to Table S1. From this process, we identified 50 most crucial features for further scrutiny in the next step.

In the subsequent step, we employed a supervised distance approach as previously described to address feature redundancy [26]. For a detailed description of this methodology, please refer to Supplementary Methods. To identify potentially redundant feature clusters, hierarchical clustering with complete linkage was applied to all features based on their supervised distances. Using a threshold of 0.75, the top 27 brain MRI structures were selected by excluding redundant features (Table S2 and Fig. S2). To assess the robustness of our feature selection approach, we tested different thresholds (0.6, 0.75, and 0.9) for redundancy removal and compared them with results without threshold filtering. The association analyses remained consistent across these different threshold settings (Table S3 and Fig. S3). The remaining features underwent a permutation-based selection procedure to reassess their ranking order. Subsequently, we implemented a sequential forward selection strategy by progressively adding variables to the foundational LightGBM model (initiated with the top feature) one at a time, resulting in decreasing MAE values. During each iteration, only the subset of features that improved MAE were retained. Finally, we identified the 20 most predictive features for establishing a model to predict brain age. Bootstrapping with 5,000 replicates was used to compute the 95% confidence intervals (CIs) for MAE and 2 other performance metrics (coefficient of determination [*R*^2^] and root mean square error [RMSE]) of the final prediction model.

Beyond the aforementioned feature selection techniques, the SHAP (SHapley Additive exPlanations) model explanation technique was also employed to pinpoint the influence of input features in brain age prediction and to quantify their respective contributions to the prediction [27].

### Brain age correction

To mitigate spurious correlations between BAG and age-related variables in subsequent analyses, a bias adjustment process was implemented [7,28,29]. Briefly, an unbiased estimate of brain age was computed by the linear correction method [30]. Let *x* be the chronological age, and *y* be the predicted brain age. We fitted a linear regression *y =* (*a +* 1) × *x + b* to the test dataset, where *a* and *b* represent the slope and intercept of a linear regression model. Then, the corrected brain age was calculated by *y* – (*a* × *x* + *b*).

### Assessment of PA

Participants were instructed to wear Axivity AX3 triaxial accelerometers on their predominant hands for a continuous span of 7 days, during which the device recorded triaxial acceleration at a frequency of 100 Hz within a dynamic range of plus or minus 8 gravitational units. Exclusion criteria for the study included participants with inadequate device utilization time (less than 72 h), suboptimal device calibration, or those experiencing a transition into or out of daylight-saving time during the monitoring interval. Weekly minutes of LPA, MPA, and VPA were classified based on time engaged in activities with intensities ranging between 30 and 125 milligravities (mg), above 125 to 400 mg, and exceeding 400 mg, respectively [17]. The aggregate duration of MVPA was calculated by summing the minutes of MPA and VPA.

### Assessment of cognitive function and brain disorders

Cognitive assessments were conducted using a touchscreen interface at the UKB Assessment Centre during the initial imaging visit. This study incorporated a suite of 10 cognitive tests, namely, reaction time, numeric memory, fluid intelligence, trail making test part A (trail making A), trail making test part B (trail making B), matrix pattern completion, tower rearranging, symbol digit substitution, prospective memory, and pairs matching.

The brain disorders contain dementia, Parkinson’s disease, stroke, depressive disorder, anxiety disorder, and bipolar affective disorder. All participants have access to comprehensive electronic health records, including hospital admissions, death registers, and primary care records. The diagnosis of incident brain disorders was determined using 3-character International Classification of Diseases-10th Revision (ICD-10) codes extracted from the UKB’s first occurrences and algorithmically defined health outcomes (including cases from hospital admissions, death registers, primary care, and self-reported data, Table S4). We excluded individuals with pre-existing brain disorders at baseline to avoid reverse causation.

### Assessment of covariates

A wide range of sociodemographic and lifestyle factors were selected based on a priori-defined directed acyclic graph (Fig. S4). Covariate data include age at the start of accelerometry measurement (calculated from date of birth and start date of accelerometer assessment), sex (female and male), ethnicity (White and non-White), educational attainment (higher education, no qualification, and any other qualification), alcohol intake frequency (daily or almost daily, 3 or 4 times a week, once or twice a week, one to 3 times a month, special occasions only, and never), smoking status (never, previous, and current), Townsend deprivation index, and dietary pattern (healthy and unhealthy). The covariates with repeated measurements (educational attainment, dietary pattern, smoking status, and alcohol intake frequency) were obtained from a touchscreen questionnaire at the time point closest to the start date of the accelerometer assessment (Fig. S5). Townsend deprivation index represents the level of deprivation on the basis of post codes. It was derived from aggregated data of unemployment, car ownership, house ownership, and household overcrowding, with higher scores indicating higher deprivation [31]. A cumulative dietary pattern was constructed to reflect the healthy dietary pattern according to previous studies (Supplementary Methods) [32,33]. Detailed information about the source of covariates used in the study is listed in Table S5.

### Statistical analysis

The generalized additive models (GAMs) were applied to examine the nonlinear relationships between PA and BAG. The GAMs for PA and BAG were established with the following formula: BAG ∼ *s* (PA) + COV, where PA represents the task performance of LPA, MPA, VPA, and MVPA. We established models for each PA and BAG separately. The term *s* () estimates the smooth effect by utilizing thin-plate regression splines that amalgamate *k* quantity of nonlinear basis functions. The count of base functions *k* was ascertained based on the model fit. The smoothed term within the model computes the comprehensive nonlinear impact of PA on the BAG. The effective degrees of freedom (EDF) were computed to estimate the extent of nonlinearity in the models. The COV is the parametric terms in the model that are the covariates to be controlled in this model. We included age, sex, ethnicity, educational attainment, Townsend deprivation index, smoking status, alcohol intake frequency, and dietary pattern in all the GAMs. The smooth effects of PA and BAG have been illustrated using fitted GAM curves and 95% CIs.

We then used a linear model to investigate the association between PA quartiles (Q1 to Q4) and the BAG, with PA quartiles as independent variables and the BAG as the dependent variable; covariates adjusted included age, sex, ethnicity, educational attainment, Townsend deprivation index, smoking status, alcohol intake frequency, and dietary pattern. To assess and compare difference in BAG between PA quartiles, we utilized the Wilcoxon rank-sum test and standardized mean differences (SMD) after adjusting the above confounders.

We also investigated the linear and nonlinear associations between PA and a total of 1,425 IDPs after adjusting for age, sex, ethnicity, educational attainment, Townsend deprivation index, smoking status, alcohol intake frequency, and dietary pattern. Standardized regression coefficient was calculated for fast comparison between variables (Supplementary Methods). False discovery rate (FDR) correction (α = 0.05) was performed for multiple comparisons.

Mediation analysis was performed to test the hypotheses of (a) whether the BAG mediated the relationships between the PA and cognitive function; (b) whether the relationships between the PA and cognitive decline were mediated through BAG; and (c) whether the relationships between the PA and brain disorders were mediated through BAG.

Statistical analyses were conducted using R v4.3.1 and Python 3.9. All statistical packages were implemented in R, including the “mgcv” package for GAMs [34], “ggplot2” for data visualization, “mediation” for mediation analyses, and “stats” for general statistical testing. Multiple comparison corrections were performed using the FDR method with α = 0.05. Python libraries including scikit-learn and scipy were used for machine learning implementations. All *P* values were 2-sided.

## Results

### Baseline characteristics

Of the 16,972 participants included in this study, the median (IQR) age was 62 (56 to 68) years, and 9,377 (55.2%) were women. The median of accelerometer-measured PA level was 33.9 h/week for LPA, 7.73 h/week for MPA, 20.2 min/week for VPA, and 8.23 h/week for MVPA (Table). The median (IQR) BAG was 0.0 (−2.6 to 2.5). Baseline characteristics of participants across PA quartiles for each intensity level are presented in Table S6.

**Table.** Baseline characteristics of study participants. Data are *n* (%) or median (IQR).

Characteristics	*N* = 16,972
Age at start of accelerometry measurement (years)	62 (56 to 68)
Sex	
Female	9,377 (55.2%)
Male	7,595 (44.8%)
Townsend deprivation index	−2.6 (−3.9 to −0.5)
Ethnicity	
Non-White	411 (2.4%)
White	16,561 (97.6%)
Educational attainment	
Higher education	8,524 (50.2%)
No qualification	919 (5.4%)
Any other qualification	7,529 (44.4%)
Smoking status	
Never smoked	10,662 (62.8%)
Previous smoker	5,735 (33.8%)
Current smoker	575 (3.4%)
Alcohol intake frequency	
Daily or almost daily	3,094 (18.2%)
Three or four times a week	4,739 (27.9%)
Once or twice a week	4,380 (25.8%)
One to three times a month	1,985 (11.7%)
Special occasions only	1,773 (10.4%)
Never	1,001 (5.9%)
Dietary pattern	
Healthy	10,237 (60.3%)
Unhealthy	6,735 (39.7%)
LPA, h/week	33.9 (29.4 to 38.8)
MPA, h/week	7.73 (5.54 to 10.4)
VPA, min/week	20.2 (10.1 to 40.3)
MVPA, h/week	8.23 (5.88 to 11.3)

LPA, light-intensity physical activity; MPA, moderate-intensity physical activity; VPA, vigorous-intensity physical activity; MVPA, moderate- to vigorous-intensity physical activity.

### Brain age prediction

**Fig. 2.**
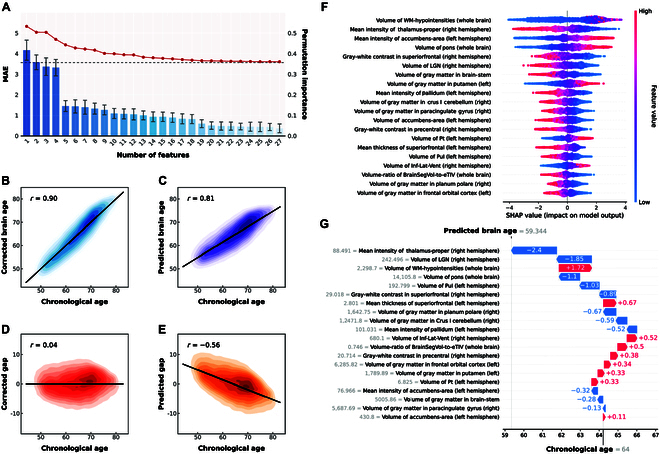
Brain age prediction model. (A) Sequential forward selection from selected subset of features after removing redundancy. The line chart represents descending MAE (left *y*-axis) upon the inclusion of features one per iteration. The bar chart exhibits descending permutation-based importance of features (right *y*-axis). MAE, mean absolute error. (B to E) Associations of brain age and brain age gap with chronological age before and after correction. Results are aggregated from 10-fold cross-validation, where each fold’s predictions were generated using models trained on the other 9 folds. For bias correction, parameters were estimated independently within each training fold and applied to the corresponding test fold. Each graph displays a kernel density plot of brain age, brain age gap plotted against chronological age, and the linear regression lines and Spearman’s correlation coefficients (*r*). (F) Distribution of the SHAP values for the top 27 features based on the highest mean absolute SHAP value. Each sample in the test set is represented as a data point per feature. The *x*-axis shows the SHAP value and the color coding reflects the feature values. (G) Individualized explanation of brain age for a single individual aged 64 years. The output value (the gray dashed line with the number at the top of the plot) shows predicted brain age for that individual. The base value (the gray dashed line with the number at the bottom of the plot) approximates chronological age (i.e., 64 years). The features in red increase brain age, and those in blue decrease it.

Among the initial set of 1,425 IDPs, the top 50 most important features were selected based on their tree-based feature importance (Table S7). Redundant features were further investigated by hierarchically clustering the subset of features according to supervised distance, such as volume of gray matter in frontal pole (left) and volume of gray matter in frontal orbital cortex (left) denoted adjacent anatomy positioning, and gave the approximate information about the prediction task (Fig. S2). Sequential forward selection demonstrated incremental improvements in MAE, while permutation importance decreased gradually within the selected feature sets (Fig. 2A). Given the diminishing returns incorporating additional features, the top 20 features were ultimately chosen for final model development. The final brain age prediction model, employing LightGBM through a 10-fold cross-validation, demonstrated an MAE of 3.65 (95% CI: 3.63 to 3.68) years, an *R*^2^ of 0.65 (95% CI: 0.64 to 0.65), and an RMSE of 4.59 (95% CI: 4.56 to 4.62). After brain age correction, enhanced performance was observed with an MAE of 3.03 (95% CI: 3.01 to 3.05) years, an *R*^2^ of 0.76 (95% CI: 0.76 to 0.77), and an RMSE of 3.78 (95% CI: 3.75 to 3.80) (Table S8). Additionally, the BAG exhibited a negative correlation with chronological age before correction (Spearman’s *r* = −0.56). After correction, the BAG was found to be orthogonal to chronological age (Spearman’s *r* = 0.04) (Fig. 2D and E). To further validate the model’s generalizability, we used MRI data from the first scan as the training set (*N* = 45,719) and the first repeat follow-up scans (*N* = 4,564) as an independent test set. The model trained on the training set achieved comparable performance on the independent test set (MAE = 3.69 [95% CI: 3.61 to 3.78] years, *R*^2^ = 0.61 [0.60 to 0.63], and RMSE = 4.62 [4.53 to 4.72]), demonstrating robust generalizability of our brain age prediction model.

Global and local SHAP plots were utilized to visualize the magnitude of each selected feature on the prediction model (Fig. 2F and G). Figure 2F provides a comprehensive overview of the top 20 features for predicting chronological age, where each participant was represented as a data point per feature. Notably, volume of WM-hypointensities (whole brain) appears to possess substantial predictive power as greater values of white matter hypointensities exhibit a higher value of predicted brain age.

### Associations between accelerometer-measured PA and BAG

**Fig. 3.**
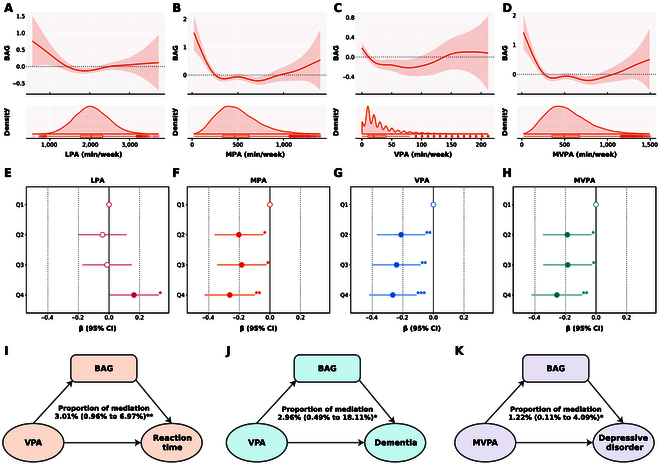
Associations between accelerometer-measured PA, brain age gap, and brain health. (A to D) Nonlinear association between PA and BAG (top subplots). The bottom subplots show kernel density for each PA in the population. (E to H) Association between PA quartile and BAG. Data are presented as β (95% CI). ^*^P < 0.05; ^**^P < 0.01; ^***^P < 0.001. Models were adjusted for age, sex, ethnicity, educational attainment, Townsend deprivation index, smoking status, alcohol consumption, and dietary pattern. (I to K) Mediation analyses for association between PA, brain health, and BAG. Cognitive function, cognitive decline, and brain disorders were selected as brain health outcomes of interest. Proportion of mediation (95% CI) was the size of the average causal mediation effects relative to the total effect. Models were adjusted for age, sex, ethnicity, educational attainment, Townsend deprivation index, smoking status, alcohol consumption, and dietary pattern. ^*^P < 0.05; ^**^P < 0.01; ^***^P < 0.001.

We found that PA was significantly correlated with BAG in U-shaped associations (Fig. 3A to D), including LPA (EDF = 3.27, *F* = 2.47, *P* = 0.04), MPA (EDF = 6.27, *F* = 6.49, *P* < 1 × 10^−300^), VPA (EDF = 5.76, *F* = 4.92, *P* = 2.58 × 10^−5^), and MVPA (EDF = 6.10, *F* = 6.45, *P* < 1 × 10^−300^), which demonstrated the unfavorable impact of insufficient or excessive PA on accelerated brain aging process (Table S9).

Furthermore, we observed the significant difference of BAG distribution across PA quartiles, including MPA (Q4 vs. Q1: *P* = 0.002; SMD = 0.065), VPA (Q4 vs. Q1: *P* = 0.012; SMD = 0.053), and MVPA (Q4 vs. Q1: *P* = 0.003; SMD = 0.061) after FDR correction, whereas the difference was not significant in LPA (*P* > 0.05) (Table S10). Higher PA quartile was significantly associated with decreased BAG, including MPA (Q4 vs. Q1: β [95% CI] = −0.26 [−0.43, −0.10]), VPA (Q4 vs. Q1: β [95% CI] = −0.27 [−0.42, −0.11]), and MVPA (Q4 vs. Q1: β [95% CI] = −0.26 [−0.42, −0.09]) (Fig. 3E to H and Table S11).

### Mediation analysis

We then investigated whether the association between PA and brain health was mediated by BAG. Firstly, we found that BAG partially mediated the effect of VPA on reaction time (proportion of mediation = 3.01% [0.96%, 6.97%]; *P* = 0.004), MVPA on reaction time (proportion of mediation = 2.50% [0.43%, 5.87%]; *P* = 0.016), and MVPA on fluid intelligence (proportion of mediation = −4.64% [−25.18%, −0.20%]; *P* = 0.04) (Fig. 3I and Table S12). Secondly, we found that BAG partially mediated the effect of VPA on dementia (proportion of mediation = 2.96% [0.49%, 18.11%]; *P* = 0.03), MVPA on depressive disorder (proportion of mediation = 1.22% [0.11%, 4.09%]; *P* = 0.024), and MPA on depressive disorder (proportion of mediation = 1.19% [0.07%, 3.89%]; *P* = 0.034) (Fig. 3J and K and Table S13).

### Associations between accelerometer-measured PA and brain structure

**Fig. 4.**
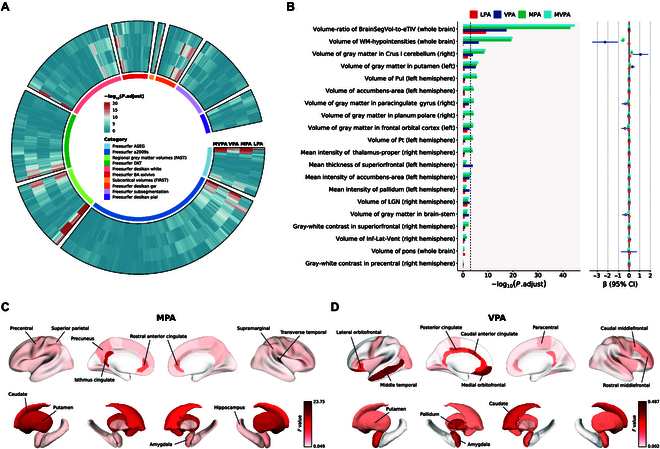
Associations between accelerometer-measured PA and brain structure. (A) A circular heatmap showing the association between PA and 1,425 IDPs. Models were adjusted for age, sex, ethnicity, educational attainment, Townsend deprivation index, smoking status, alcohol consumption, and dietary pattern. *P* was adjusted for FDR correction (α = 0.05). (B) Association between PA and the top 20 IDPs that were incorporated in the brain age prediction model. The left bar chart represents descending significance of features correlated with PA. The right point chart shows corresponding β (95% CI). Models were adjusted for age, sex, ethnicity, educational attainment, Townsend deprivation index, smoking status, alcohol consumption, and dietary pattern. *P* was adjusted for FDR correction (α = 0.05). (C and D) Cortical and subcortical regions with their volume that were nonlinearly associated with PA adjusted for age, sex, ethnicity, educational attainment, Townsend deprivation index, smoking status, alcohol consumption, and dietary pattern.

The associations between PA and 1,425 IDPs across 10 main categories were visually demonstrated in Fig. 4A. Associations between PA and the top 20 features for final brain age prediction model were particularly investigated (Fig. 4B and Table S14). In particular, we found that the volume ratio of BrainSegVol-to-eTIV (whole brain), namely, the brain segmentation volumes (sum of the volume of the structures identified in the ASEG atlas) divided by the estimated total intracranial volume, was strongly associated with 4 intensities of PA after FDR correction. We also found that the volume of WM-hypointensities (whole brain), namely, hypointensity in white matter areas, was significantly positively negatively associated with MPA (β [95% CI] = −0.59 [−0.78, −0.41]; *P* = 4.07 × 10^−9^), VPA (β [95% CI] = −2.27 [−3.47, −1.07]; *P* = 0.002), and MVPA (β [95% CI] = −0.56 [−0.73, −0.38]; *P* = 2.53 × 10^−9^) after FDR correction (Table S14). Cortical areas of the isthmus cingulate (left hemisphere: EDF = 1.00, *F* = 16.84, *P* = 0.001), rostral anterior cingulate (left hemisphere: EDF = 1.26, *F* = 9.03, *P* = 0.013; right hemisphere: EDF = 1.00, *F* = 7.39, *P* = 0.027), and transverse temporal (right hemisphere: EDF = 1.00, *F* = 23.75, *P* = 8.47 × 10^−5^) showed the most significant nonlinear or linear association with MPA, while subcortical areas of the caudate (left hemisphere: EDF = 1.00, *F* = 14.24, *P* = 6.15 × 10^−4^; right hemisphere: EDF = 1.55, *F* = 11.86, *P* = 6.42 × 10^−5^) and putamen (left hemisphere: EDF = 1.00, *F* = 17.54, *P* = 1.33 × 10^−4^; right hemisphere: EDF = 1.00, *F* = 17.50, *P* = 1.33 × 10^−4^) showed the most significant association with MPA after FDR correction (Fig. 4C and Table S15).

## Discussion

In this largest population-based study of neuroimaging data, we developed a prediction model of brain age by LightGBM, which achieved an appreciable performance. Moreover, we found a U-shaped association between objectively measured PA and BAG, suggesting that moderate amounts of PA were associated with reduced brain aging, irrespectively of PA intensities. Our findings provided valuable insights into engaging moderate PA as an evidence-driven preventive strategy to improve brain health.

While numerous studies have explored the relationship between PA and brain health, most have focused on specific pathological states or isolated MRI indicators. For instance, a prospective study with 1,604 US participants found that increased PA levels have been linked to reduced cerebrovascular lesions and improved white matter integrity [35]. In addition, a meta-analysis demonstrated that randomized clinical trials involving exercise led to enhancements in cognitive performance compared to controls in late adulthood. The effects, while modest, were most pronounced in executive function [36]. However, these individual indicators or specific disorders provide only a fragmented view of brain health. Brain age, derived from neuroimaging data, serves as a holistic indicator that captures the complete spectrum of brain aging profiles—from accelerated to decelerated aging [29,37]. With the increasing application of MRI in large population cohorts, brain age has emerged as a comprehensive marker of overall brain health [3]. Our study advances this field in 2 key aspects. First, by using BAG as an integrative measure, we provide a more comprehensive assessment of how PA influences brain health status. Second, while previous research has predominantly documented the benefits of PA, our finding of a U-shaped relationship reveals the potential adverse effects of excessive PA on brain aging, an aspect that has received limited attention in prior studies.

Notably, our study emphasized that the utilization of objectively measured PA data via accelerometry allowed for an authentic examination of the association between PA and brain health. While previous studies using self-reported PA measures have established general associations between PA and brain MRI metrics [38,39], questionnaire-based assessments often face limitations in accurately quantifying activity intensities and durations [40]. The use of accelerometry in our study enabled more objective and detailed measurement of PA patterns across various intensity levels. Therefore, our findings held the potential to substantially impact the field, providing valuable insights and contributing to the existing body of knowledge.

The biological mechanisms underlying the U-shaped relationship between PA and brain health remain ambiguous, but several previous biological studies may contribute to explaining the results of our study. For insufficient PA, sedentary behavior may compromise brain health through multiple pathways. Physical inactivity reduces cerebral blood flow and neuroplasticity, potentially limiting the delivery of nutrients and oxygen to brain tissues [41]. Additionally, insufficient PA may lead to decreased production of brain-derived neurotrophic factor, a crucial protein that stimulates cell growth and maintains neurons [42]. On the other hand, excessive PA might accelerate brain aging through distinct mechanisms. Extreme exercise can induce oxidative stress and trigger inflammatory responses in the brain, potentially causing accelerated aging in the brain [43,44]. The interaction between PA and brain health likely involves complex modulation of insulin/glucose signaling pathways, which can be compromised by both insufficient and excessive PA [45,46].

Our mediation analysis revealed that BAG serves as a significant mediator between PA and brain-related outcomes. This finding suggests that PA’s effects on cognitive function and brain disorders may operate through pathways that manifest as structural brain changes captured by BAG. Furthermore, BAG’s mediating role supports its utility as an early indicator of brain modifications induced by lifestyle factors, potentially preceding clinical manifestations of cognitive decline or neurological disorders. This finding aligns with the emerging concept of BAG as a sensitive biomarker for monitoring brain health interventions and adds to our understanding of how lifestyle modifications might translate into brain health outcomes. Future research should prioritize 3 critical directions: (a) longitudinal investigations to establish causality between PA patterns and brain aging trajectories; (b) intervention studies determining optimal PA thresholds across different age groups; and (c) mechanistic studies exploring the biological pathways, particularly focusing on inflammatory and oxidative stress mechanisms, through which PA influences brain aging. These studies will help develop more precise recommendations for maintaining optimal brain health.

In this study, the ensemble learning algorithm was employed to construct brain age using over a thousand MRI data from the UKB. However, there is a lack of studies that apply the LightGBM model for brain age construction. Compared to other conventional integration algorithm models, LightGBM exhibits higher accuracy [47–49]. Meanwhile, with its parallel learning capabilities, LightGBM effectively manages large-scale datasets and supports the direct utilization of category features, eliminating the need for manual encoding or preprocessing [19]. In this study, the brain age model that we developed exhibited superior performance. Our nonadjusted brain age prediction model achieved an MAE of 3.69 years, an RMSE of 0.05, and an *R*^2^ of 0.64. Consequently, this brain age model possesses considerable potential for future application in the prevention and early intervention of brain-related disorders.

The present study has certain limitations. Firstly, the cross-sectional nature of the data source precludes establishing causal associations, necessitating further longitudinal sequential studies for adequate confirmation. Secondly, the UKB cohort may not fully represent the general population due to its predominantly White demographic and certain distinctive characteristics. Because of the low response rates (5.5%) of UKB and the healthy volunteer effect, we acknowledged that the sample in our study may not be representative of the middle-aged and older UK population, which may limit the generalizability of the current findings [50]. However, the recent evidence strongly supported the notion that any potential bias resulting from poor representation would have minimal impact on estimates of the strength of the associations [51].

## Conclusion

Using the largest population-based sample of neuroimaging data and the recent popular machine learning technique named LightGBM, we achieved a relatively high prediction accuracy of brain age. More importantly, we found a U-shaped association between objectively measured PA and BAG, suggesting that moderate amounts of PA were associated with reduced brain aging, irrespectively of PA intensities. Our findings provide a comprehensive insight into the association between PA and brain health, which could facilitate the development of personalized preventive strategy for brain health.

## Ethical Approval

UKB received ethical approval from the National Information Governance Board for Health and Social Care and the National Health Service North West Multi-Center Research Ethics Committee. All participants gave informed consent through electronic signature before enrollment in the study. Analyses were conducted under application number 79095. This research conforms to the Declaration of Helsinki.

## Data Availability

This research has been conducted using the UK Biobank Resource under application number 79095. The UK Biobank data are available on application to the UK Biobank (www.ukbiobank.ac.uk) with access fees.

## References

[1] Mishra S, Beheshti I, Khanna P. A review of neuroimaging-driven brain age estimation for identification of brain disorders and health conditions. IEEE Rev Biomed Eng. 2023;16:371–385. 34428153 10.1109/RBME.2021.3107372

[2] Raz N, Rodrigue KM. Differential aging of the brain: Patterns, cognitive correlates and modifiers. Neurosci Biobehav Rev. 2006;30(6):730–748. 16919333 10.1016/j.neubiorev.2006.07.001 PMC6601348

[3] Cole JH, Franke K. Predicting age using neuroimaging: Innovative brain ageing biomarkers. Trends Neurosci. 2017;40(12):681–690. 29074032 10.1016/j.tins.2017.10.001

[4] Leonardsen EH, Peng H, Kaufmann T, Agartz I, Andreassen OA, Celius EG, Espeseth T, Harbo HF, Høgestøl EA, Lange AM, et al. Deep neural networks learn general and clinically relevant representations of the ageing brain. NeuroImage. 2022;256: Article 119210. 35462035 10.1101/2021.10.29.21265645 PMC7614754

[5] Han LKM, Dinga R, Hahn T, Ching CRK, Eyler LT, Aftanas L, Aghajani M, Aleman A, Baune BT, Berger K, et al. Brain aging in major depressive disorder: Results from the ENIGMA major depressive disorder working group. Mol Psychiatry. 2021;26(9):5124–5139. 32424236 10.1038/s41380-020-0754-0 PMC8589647

[6] Constantinides C, Han LKM, Alloza C, Antonucci LA, Arango C, Ayesa-Arriola R, Banaj N, Bertolino A, Borgwardt S, Bruggemann J, et al. Brain ageing in schizophrenia: Evidence from 26 international cohorts via the ENIGMA schizophrenia consortium. Mol Psychiatry. 2023;28(3):1201–1209. 36494461 10.1038/s41380-022-01897-w PMC10005935

[7] Cole JH, Ritchie SJ, Bastin ME, Valdés Hernández MC, Muñoz Maniega S, Royle N, Corley J, Pattie A, Harris SE, Zhang Q, et al. Brain age predicts mortality. Mol Psychiatry. 2018;23(5):1385–1392. 28439103 10.1038/mp.2017.62 PMC5984097

[8] Cole JH, Underwood J, Caan MWA, de Francesco D, van Zoest RA, Leech R, Wit FWNM, Portegies P, Geurtsen GJ, Schmand BA, et al. Increased brain-predicted aging in treated HIV disease. Neurology. 2017;88(14):1349–1357. 28258081 10.1212/WNL.0000000000003790 PMC5379929

[9] Linli Z, Feng J, Zhao W, Guo S. Associations between smoking and accelerated brain ageing. Prog Neuro-Psychopharmacol Biol Psychiatry. 2022;113: Article 110471. 10.1016/j.pnpbp.2021.110471 34740709

[10] Wrigglesworth J, Ward P, Harding IH, Nilaweera D, Wu Z, Woods RL, Ryan J. Factors associated with brain ageing—A systematic review. BMC Neurol. 2021;21:312. 34384369 10.1186/s12883-021-02331-4 PMC8359541

[11] Neves LM, Ritti-Dias R, Juday V, Marquesini R, Gerage AM, Laurentino GC, Nunes RH, Stubb B, Ugriniwitsch C. Objective physical activity accumulation and brain volume in older adults: An MRI and whole-brain volume study. J Gerontol A Biol Sci Med Sci. 2023;78:902–910. 35857361 10.1093/gerona/glac150

[12] Festa F, Medori S, Macrì M. Move your body, boost your brain: The positive impact of physical activity on cognition across all age groups. Biomedicines. 2023;11(6):1765. 37371860 10.3390/biomedicines11061765 PMC10296541

[13] Tan ZS, Spartano NL, Beiser AS, DeCarli C, Auerbach SH, Vasan RS, Seshadri S. Physical activity, brain volume, and dementia risk: The Framingham study. J Gerontol A Biol Sci Med Sci. 2017;72(6):789–795. 27422439 10.1093/gerona/glw130 PMC6075525

[14] Erickson KI, Leckie RL, Weinstein AM. Physical activity, fitness, and gray matter volume. Neurobiol Aging. 2014;35(Suppl 2):S20–S28. 24952993 10.1016/j.neurobiolaging.2014.03.034 PMC4094356

[15] Steffener J, Habeck C, O’Shea D, Razlighi Q, Bherer L, Stern Y. Differences between chronological and brain age are related to education and self-reported physical activity. Neurobiol Aging. 2016;40:138–144. 26973113 10.1016/j.neurobiolaging.2016.01.014 PMC4792330

[16] Welk GJ, Bai Y, Lee J-M, Godino J, Saint-Maurice PR, Carr L. Standardizing analytic methods and reporting in activity monitor validation studies. Med Sci Sports Exerc. 2019;51(8):1767–1780. 30913159 10.1249/MSS.0000000000001966 PMC6693923

[17] Strain T, Wijndaele K, Dempsey PC, Sharp SJ, Pearce M, Jeon J, Lindsay T, Wareham N, Brage S. Wearable-device-measured physical activity and future health risk. Nat Med. 2020;26(9):1385–1391. 32807930 10.1038/s41591-020-1012-3 PMC7116559

[18] Sanders A-M, Richard G, Kolskår K, Ulrichsen KM, Kaufmann T, Alnæs D, Beck D, Dørum ES, de Lange AMG, Egil Nordvik J, et al. Linking objective measures of physical activity and capability with brain structure in healthy community dwelling older adults. NeuroImage Clin. 2021;31: Article 102767. 34330086 10.1016/j.nicl.2021.102767 PMC8329542

[19] Ke, G, Meng Q, Finley T, Wang T, Chen W, MA W, Ye C, Liu T-C. LightGBM: A highly efficient gradient boosting decision tree. In: *Neural Information Processing Systems*. Red Hook (NY): Curran Associates Inc.;2017.

[20] Sudlow C, Gallacher J, Allen N, Beral V, Burton P, Danesh J, Downey P, Elliott P, Green J, Landray M, et al. UK Biobank: An open access resource for identifying the causes of a wide range of complex diseases of middle and old age. PLOS Med. 2015;12(3): Article e1001779. 25826379 10.1371/journal.pmed.1001779 PMC4380465

[21] Miller KL, Alfaro-Almagro F, Bangerter NK, Thomas DL, Yacoub E, Xu J, Bartsch AJ, Jbabdi S, Sotiropoulos SN, Andersson JLR, et al. Multimodal population brain imaging in the UK Biobank prospective epidemiological study. Nat Neurosci. 2016;19(11):1523–1536. 27643430 10.1038/nn.4393 PMC5086094

[22] Doherty A, Jackson D, Hammerla N, Plötz T, Olivier P, Granat MH, White T, van Hees VT, Trenell MI, Owen CG, et al. Large scale population assessment of physical activity using wrist worn accelerometers: The UK Biobank study. PLOS ONE. 2017;12(2): Article e0169649. 28146576 10.1371/journal.pone.0169649 PMC5287488

[23] Alfaro-Almagro F, Jenkinson M, Bangerter NK, Andersson JLR, Griffanti L, Douaud G, Sotiropoulos SN, Jbabdi S, Hernandez-Fernandez M, Vallee E, et al. Image processing and quality control for the first 10,000 brain imaging datasets from UK Biobank. NeuroImage. 2018;166:400–424. 29079522 10.1016/j.neuroimage.2017.10.034 PMC5770339

[24] Elliott LT, Sharp K, Alfaro-Almagro F, Shi S, Miller KL, Douaud G, Marchini J, Smith SM. Genome-wide association studies of brain imaging phenotypes in UK Biobank. Nature. 2018;562(7726):210–216. 30305740 10.1038/s41586-018-0571-7 PMC6786974

[25] Wang C, Wu Q, Huang S, Saied A. Economical hyperparameter optimization with blended search strategy. In: *The Ninth International Conference on Learning Representations (ICLR 2021)*. OpenReview.net (online). 2021.

[26] Qiu W, Chen H, Dincer AB, Lundberg S, Kaeberlein M, Lee SI. Interpretable machine learning prediction of all-cause mortality. Commun Med. 2022;2:125. 36204043 10.1038/s43856-022-00180-x PMC9530124

[27] Lundberg, SM, Lee S-I. A unified approach to interpreting model predictions. In: *Proceedings of the 31st International Conference on Neural Information Processing Systems*. Red Hook (NY): Curran Associates Inc.; 2017. p. 4768–4777.

[28] Beheshti I, Nugent S, Potvin O, Duchesne S. Bias-adjustment in neuroimaging-based brain age frameworks: A robust scheme. NeuroImage Clin. 2019;24: Article 102063. 31795063 10.1016/j.nicl.2019.102063 PMC6861562

[29] Cole JH, Leech R, Sharp DJ, for the Alzheimer’s Disease Neuroimaging Initiative. Prediction of brain age suggests accelerated atrophy after traumatic brain injury. Ann Neurol. 2015;77(4):571–581. 25623048 10.1002/ana.24367 PMC4403966

[30] De Lange A-MG, Cole JH. Commentary: Correction procedures in brain-age prediction. NeuroImage Clin. 2020;26: Article 102229. 32120292 10.1016/j.nicl.2020.102229 PMC7049655

[31] Grambsch PM, Therneau TM. Proportional hazards tests and diagnostics based on weighted residuals. Biometrika. 1994;81:515–526.

[32] Mozaffarian D. Dietary and policy priorities for cardiovascular disease, diabetes, and obesity: A comprehensive review. Circulation. 2016;133(2):187–225. 26746178 10.1161/CIRCULATIONAHA.115.018585 PMC4814348

[33] Morris MC, Tangney CC, Wang Y, Sacks FM, Bennett DA, Aggarwal NT. MIND diet associated with reduced incidence of Alzheimer’s disease. Alzheimers Dement. 2015;11(9):1007–1014. 25681666 10.1016/j.jalz.2014.11.009 PMC4532650

[34] Pedersen EJ, Miller DL, Simpson GL, Ross N. Hierarchical generalized additive models in ecology: An introduction with mgcv. PeerJ. 2019;7: Article e6876. 31179172 10.7717/peerj.6876 PMC6542350

[35] Palta P, Sharrett AR, Gabriel KP, Gottesman RF, Folsom AR, Power MC, Evenson KR, Jack CR Jr, Knopman DS, Mosley TH, et al. Prospective analysis of leisure-time physical activity in midlife and beyond and brain damage on MRI in older adults. Neurology. 2021;96:e964–e974. 33408144 10.1212/WNL.0000000000011375 PMC8055339

[36] Colcombe S, Kramer AF. Fitness effects on the cognitive function of older adults: A meta-analytic study. Psychol Sci. 2003;14(2):125–130. 12661673 10.1111/1467-9280.t01-1-01430

[37] Franke K, Ten Gaser C. Years of BrainAGE as a neuroimaging biomarker of brain aging: What insights have we gained? Front Neurol. 2019;10:789. 31474922 10.3389/fneur.2019.00789 PMC6702897

[38] Spartano NL, Davis-Plourde KL, Himali JJ, Andersson C, Pase MP, Maillard P, DeCarli C, Murabito JM, Beiser AS, Vasan RS, et al. Association of accelerometer-measured light-intensity physical activity with brain volume: The Framingham Heart Study. JAMA Netw Open. 2019;2(4): Article e192745. 31002329 10.1001/jamanetworkopen.2019.2745 PMC6481600

[39] Hamer M, Sharma N, Batty GD. Association of objectively measured physical activity with brain structure: UK Biobank study. J Intern Med. 2018;284(4):439–443. 29776014 10.1111/joim.12772

[40] Steene-Johannessen J, Anderssen SA, van der Ploeg HP, Hendriksen IJM, Donnelly AE, Brage S, Ekelund U. Are self-report measures able to define individuals as physically active or inactive? Med Sci Sports Exerc. 2016;48(2):235–244. 26322556 10.1249/MSS.0000000000000760 PMC6235100

[41] Boecker H. On the emerging role of neuroimaging in determining functional and structural brain integrity induced by physical exercise: Impact for predictive, preventive, and personalized medicine. EPMA J. 2011;2(3):277–285. 23199163 10.1007/s13167-011-0093-y PMC3405390

[42] Håkansson K, Ledreux A, Daffner K, Terjestam Y, Bergman P, Carlsson R, Kivipelto M, Winblad B, Granholm AC, Mohammed AKH. BDNF responses in healthy older persons to 35 minutes of physical exercise, cognitive training, and mindfulness: Associations with working memory function. J Alzheimers Dis. 2016;55(2):645–657. 10.3233/JAD-160593 PMC6135088 27716670

[43] Powers SK, Deminice R, Ozdemir M, Yoshihara T, Bomkamp MP, Hyatt H. Exercise-induced oxidative stress: Friend or foe? J Sport Health Sci. 2020;9(5):415–425. 32380253 10.1016/j.jshs.2020.04.001 PMC7498668

[44] Sangüesa G, Batlle M, Muñoz-Moreno E, Soria G, Alcarraz A, Rubies C, Sitjà-Roqueta L, Solana E, Martínez-Heras E, Meza-Ramos A, et al. Intense long-term training impairs brain health compared with moderate exercise: Experimental evidence and mechanisms. Ann N Y Acad Sci. 2022;1518(1):282–298. 36256544 10.1111/nyas.14912 PMC10092505

[45] Erickson KI, Hillman CH, Kramer AF. Physical activity, brain, and cognition. Curr Opin Behav Sci. 2015;4:27–32.

[46] Bagi Z, Broskova Z, Feher A. Obesity and coronary microvascular disease—Implications for adipose tissue-mediated remote inflammatory response. Curr Vasc Pharmacol. 2014;12(3):453–461. 24846234 10.2174/1570161112666140423221843 PMC4167722

[47] Cumplido-Mayoral I, García-Prat M, Operto G, Falcon C, Shekari M, Cacciaglia R, Milà-Alomà M, Lorenzini L, Ingala S, Meije Wink A, et al. Biological brain age prediction using machine learning on structural neuroimaging data: Multi-cohort validation against biomarkers of Alzheimer’s disease and neurodegeneration stratified by sex. eLife. 2023;12: Article e81067. 37067031 10.7554/eLife.81067 PMC10181824

[48] Elliott ML, Belsky DW, Knodt AR, Ireland D, Melzer TR, Poulton R, Ramrakha S, Caspi A, Moffitt TE, Hariri AR. Brain-age in midlife is associated with accelerated biological aging and cognitive decline in a longitudinal birth cohort. Mol Psychiatry. 2021;26:3829–3838. 31822815 10.1038/s41380-019-0626-7 PMC7282987

[49] Liew S-L, Schweighofer N, Cole JH, Zavaliangos-Petropulu A, Tavenner BP, Han LKM, Hahn T, Schmaal L, Donnelly MR, Jeong JN, et al. Association of brain age, lesion volume, and functional outcome in patients with stroke. Neurology. 2023;100(20):e2103–e2113. 37015818 10.1212/WNL.0000000000207219 PMC10186236

[50] Fry A, Littlejohns TJ, Sudlow C, Doherty N, Adamska L, Sprosen T, Collins R, Allen NE. Comparison of sociodemographic and health-related characteristics of UK Biobank participants with those of the general population. Am J Epidemiol. 2017;186(9):1026–1034. 28641372 10.1093/aje/kwx246 PMC5860371

[51] Stamatakis E, Owen KB, Shepherd L, Drayton B, Hamer M, Bauman AE. Is cohort representativeness passé? Poststratified associations of lifestyle risk factors with mortality in the UK Biobank. Epidemiology. 2021;32(2):179–188. 33492009 10.1097/EDE.0000000000001316 PMC7850587

