# Supplementary material for: Accelerometer-Measured Physical Activity and Neuroimaging-Driven Brain Age
Source: Health Data Sci. 2025 May 2;5:0257. doi: 10.34133/hds.0257 (PMC12046135; doi:10.34133/hds.0257)
Supplement: Supplementary 1 — Supplementary Methods Figs. S1 to S5 Tables S1 to S15 [file hds.0257.f1.docx]

**Supplementary Materials**

**Supplementary Methods**

**Fig. S1. Flowchart of participants included in the analysis**

**Fig. S2. Dendrogram of hierarchical clustering based on supervised distances**

**Fig. S3. Linear regression analyses across different threshold settings**

**Fig. S4. The directed acyclic graph to guide covariate selection**

**Fig. S5. Timeline of study design**

**Table S1. Hyperparameter search space and final adopted hyperparameters for LightGBM regressor**

**Table S2. Sequential forward selection of top 27 features**

**Table S3. Nonlinear associations between PA and BAG across different threshold settings**

**Table S4. Codes used for brain disorders diagnosis and classification in the UKB**

**Table S5. Codes used for covariates in the UKB**

**Table S6. Baseline characteristics** **according to quartiles of LPA, MPA, VPA, and MVPA**

**Table S7.** **Tree-based feature importance ranking of 1425 IDPs**

**Table S8.** **Final model performance before and after brain age correction**

**Table S9.** **Nonlinear associations between PA and BAG**

**Table S10.** **The difference in BAG between PA quartile**

**Table S11.** **Linear associations between PA quartile and BAG**

**Table S12.** **Mediation analysis between PA, cognitive function, and BAG**

**Table S13.** **Mediation analysis between PA, brain disorders, and BAG**

**Table S14.** **Linear associations between PA and 20 selected features**

**Table S15.** **Nonlinear associations between PA and cortical and subcortical brain structure**

**Supplementary Methods**

**Participants, accelerometer assessment and brain imaging data**

The UK Biobank is a large population-based prospective cohort that recruited approximately 500,000 participants aged 40-69 years in the UK between 2006 and 2010. Further details of the study rationale, design, and survey methods for UK Biobank are available online (www.ukbiobank.ac.uk). Between February 2013 and December 2015, 236,519 UK Biobank participants were invited to participate in the accelerometer study. A total of 106,053 participants agreed to participate and were provided with a wrist-worn Axivity AX3 accelerometer. The accelerometer was set up to start at 10 a.m. two working days after postal dispatch, and capture triaxial acceleration data over a seven-day period at 100Hz with a dynamic range of ± 8 gravity (9.8 m/s^2^). Participants were instructed to wear the device on their dominant wrist continuously for seven days while continuing with their usual activities. Participants were asked to mail the device in a pre-paid envelope back to the coordinating centers, after the seven-day monitoring period. A total of 103,712 raw accelerometer datasets were received for data analysis. More details about data processing and data analysis have been published elsewhere.

Based on the data quality metrics provided by the UK Biobank accelerometer working group, the exclusion criteria are as follows: (1) those data flagged by UK Biobank as being unreliable due to unexpectedly small or large size (Field ID: 90002; n= 4694); (2) those with accelerometry data for less than 72 h or did not provide data for all 1-h periods within a 24-h cycle during the 7-day data collection (Field ID: 90015; n=6991); (3) those data identified by UK Biobank as not well-calibrated (Field ID: 90016; n=11); (4) those data were recalibrated using the previous accelerometer record from the same device worn by a different participant (Field ID: 90017; n=3046); (5) those data with a non-zero count of interrupted recording periods (Field ID: 90180; n=2268); (6) those data with more than 768 (Q3 + 1.5 × IQR) data recording errors (Field ID: 90182; n=1236).

**Feature importance ranking**

Predictor ranking, also known as feature importance ranking, was calculated using a built-in function within the LightGBM algorithm. The LightGBM algorithm, in general, is an ensembled tree-based model that contains a bunch of decision tree models. Hence, the feature importance can be quantified by the frequency of attributes taken as split nodes, known as the model’s “cover”. The more an attribute is used as a split node, the higher its relative importance to the prediction model. The importance can be calculated explicitly for each feature in the whole dataset, allowing them to be ranked and compared to each other.

**Ensemble learning algorithm (LightGBM)**

The LightGBM (lightweight gradient boosted machines) algorithm is currently one of the most popular machine learning techniques in the data scientist community. It is an example of ensemble learning methods that are constructed based on numerous underlying base learners, usually decision tree models. The LightGBM works by starting from a weak base learner (decision tree) and sequentially training each new tree to correct the errors from the previously trained ones. In such a manner, the predictions can be added up to produce a strong overall final predictive model. During validation, the output probability of a participant being classified into either class is calculated by averaging over the outcomes obtained from applying each individual decision tree to this participant.

**SHAP model explanation**

Ensemble learning models have been denounced due to their uninterpretable results, and are often referred to as “black-box” models. This is primarily due to their highly complex inherent structures. The SHapley Additive exPlanations (SHAP) is an available visualization tool to unpack different prediction models. On the one hand, the SHAP plot captures the extent to which each predictor contributes to the target variable; on the other, it transparentizes the prediction process by localizing all participants within the dataset, thereby allowing readers to pinpoint and compare the impact of a specific predictor on each participant. In this study, we employed a SHAP plot to visualize and interpret the roles and impacts of the selected predictors on the proposed ensemble learning model.

**Standardized regression coefficient**

Standardized regression coefficients, also called beta coefficients or beta weights, are the estimates resulting from a regression analysis where the underlying data have been standardized so that the variances of dependent and independent variables are equal to 1. Therefore, standardized coefficients are unitless and refer to how many standard deviations a dependent variable will change, per standard deviation increase in the predictor variable. The standardized coefficient simply results as:

$$\text{β*=}\frac{\text{S}_{\text{x}}}{\text{S}_{\text{y}}}\text{β}$$

Where *S_x_* and *S_y_* are the (estimated) standard deviations of *x* and *y*, respectively.

**Fig. S1. Flowchart of participants included in the analysis**

**
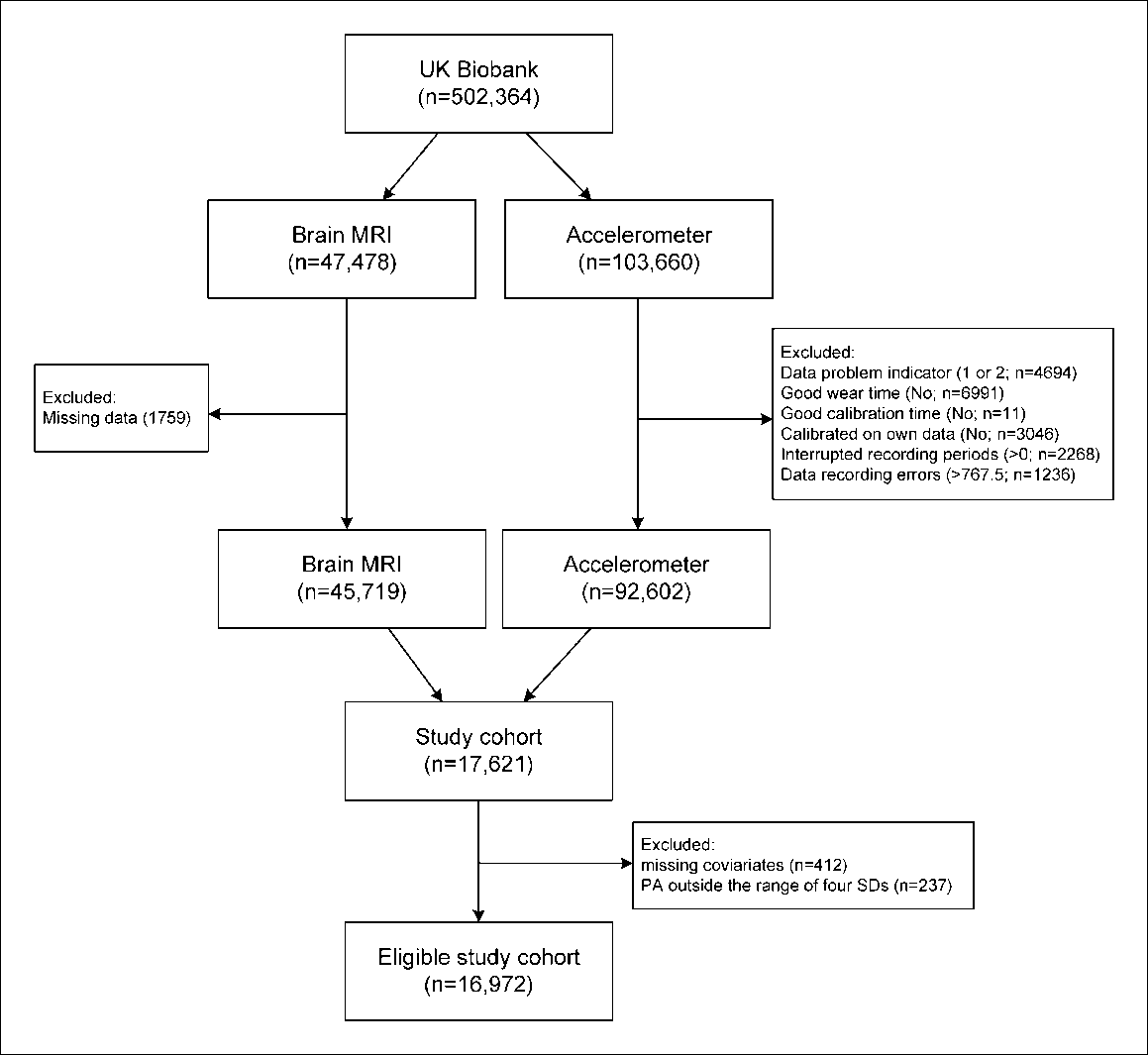
**

Flow diagram of participants selection, inclusion, and reason for exclusion.

**Fig. S2. Dendrogram of hierarchical clustering based on supervised distances**

**
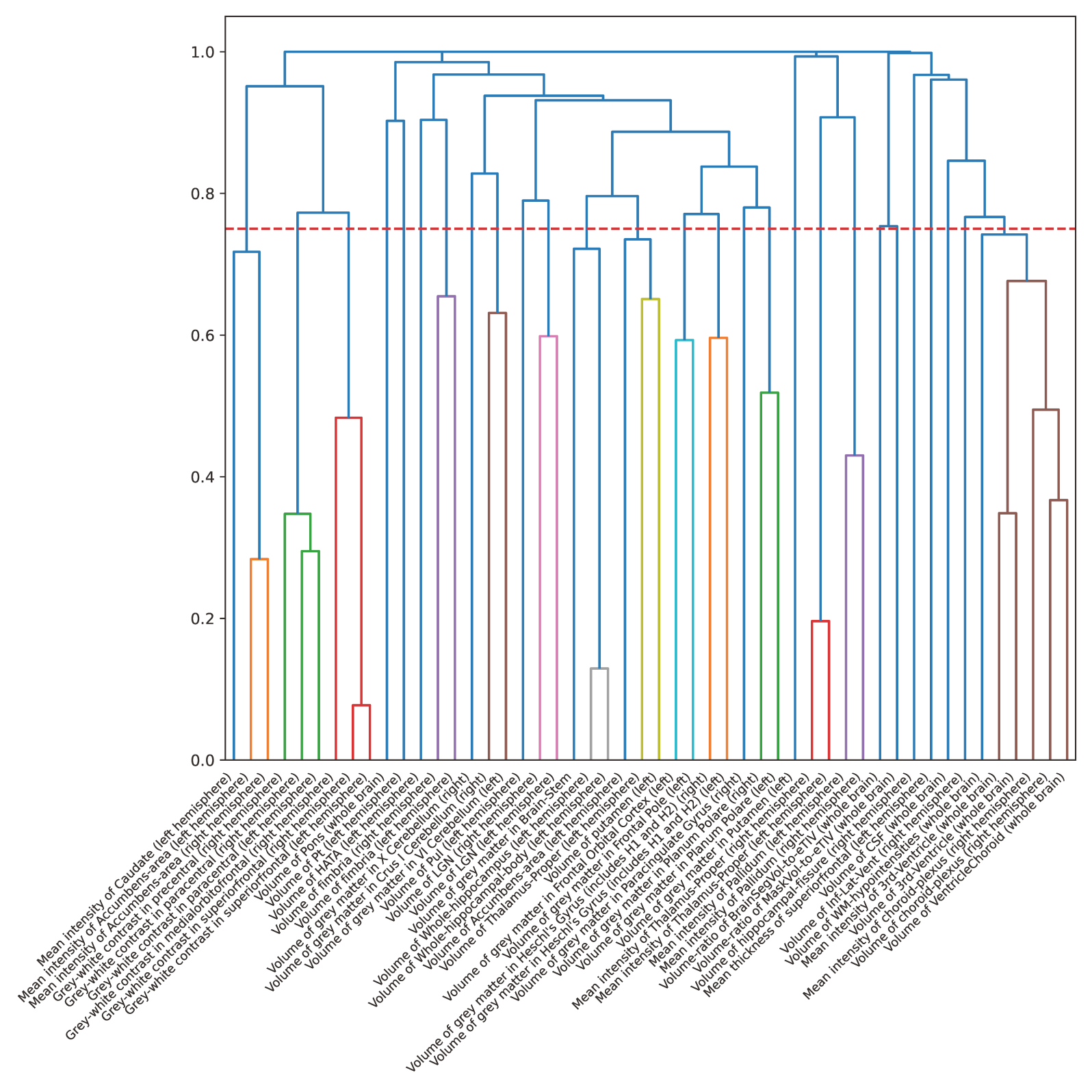
**

The horizontal dash line, 0.75, was the cutoff of clusters, and only one predictor was chosen within each cluster (grouped predictors under a threshold of 0.75).

**Fig. S3. Linear regression analyses across different threshold settings**

**
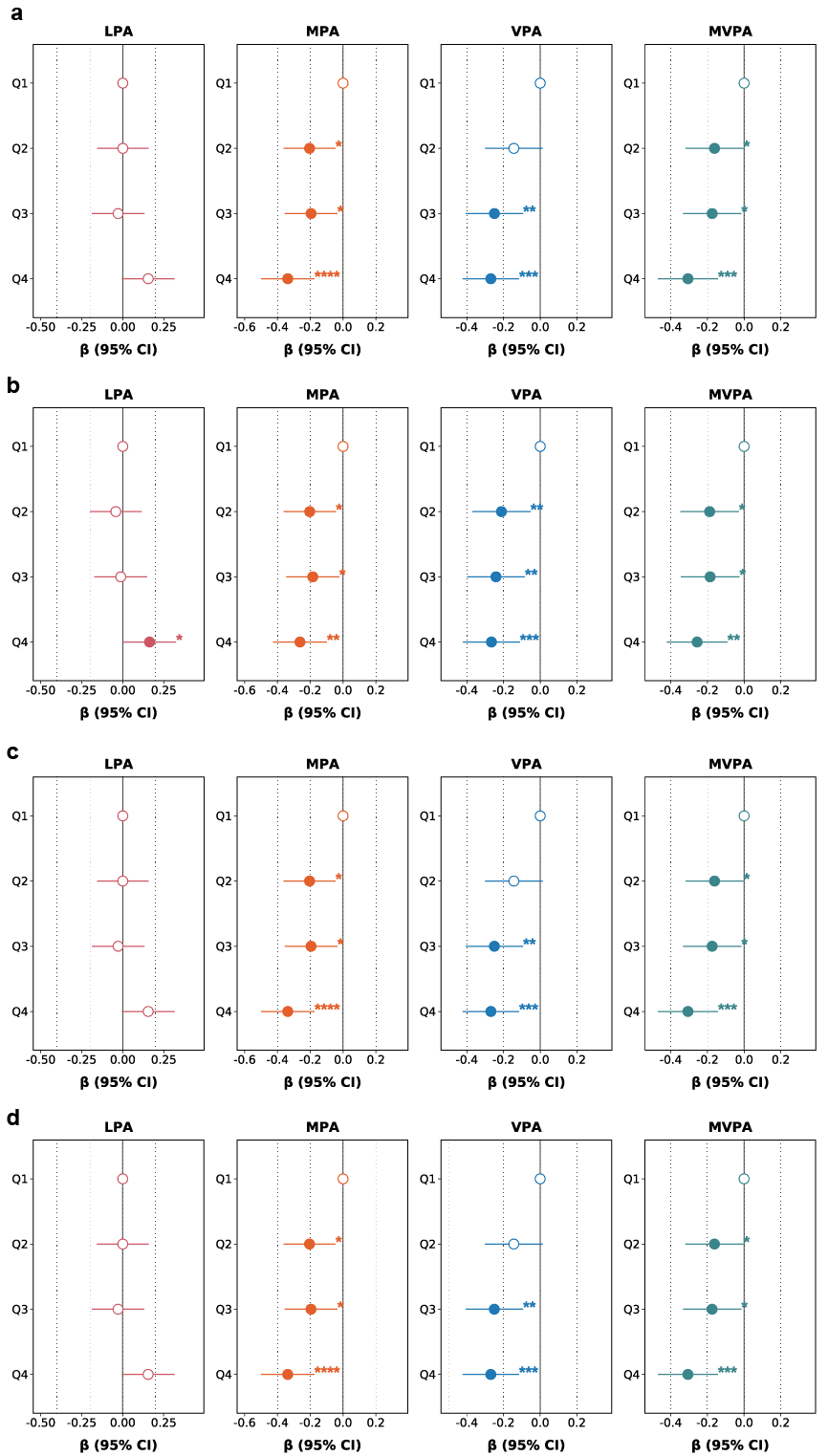
**

**a.** 0.6 threshold **b.** 0.75 threshold **c.** 0.9 threshold **d.** without thresholding

**Fig. S4. The directed acyclic graph to guide covariate selection**

**
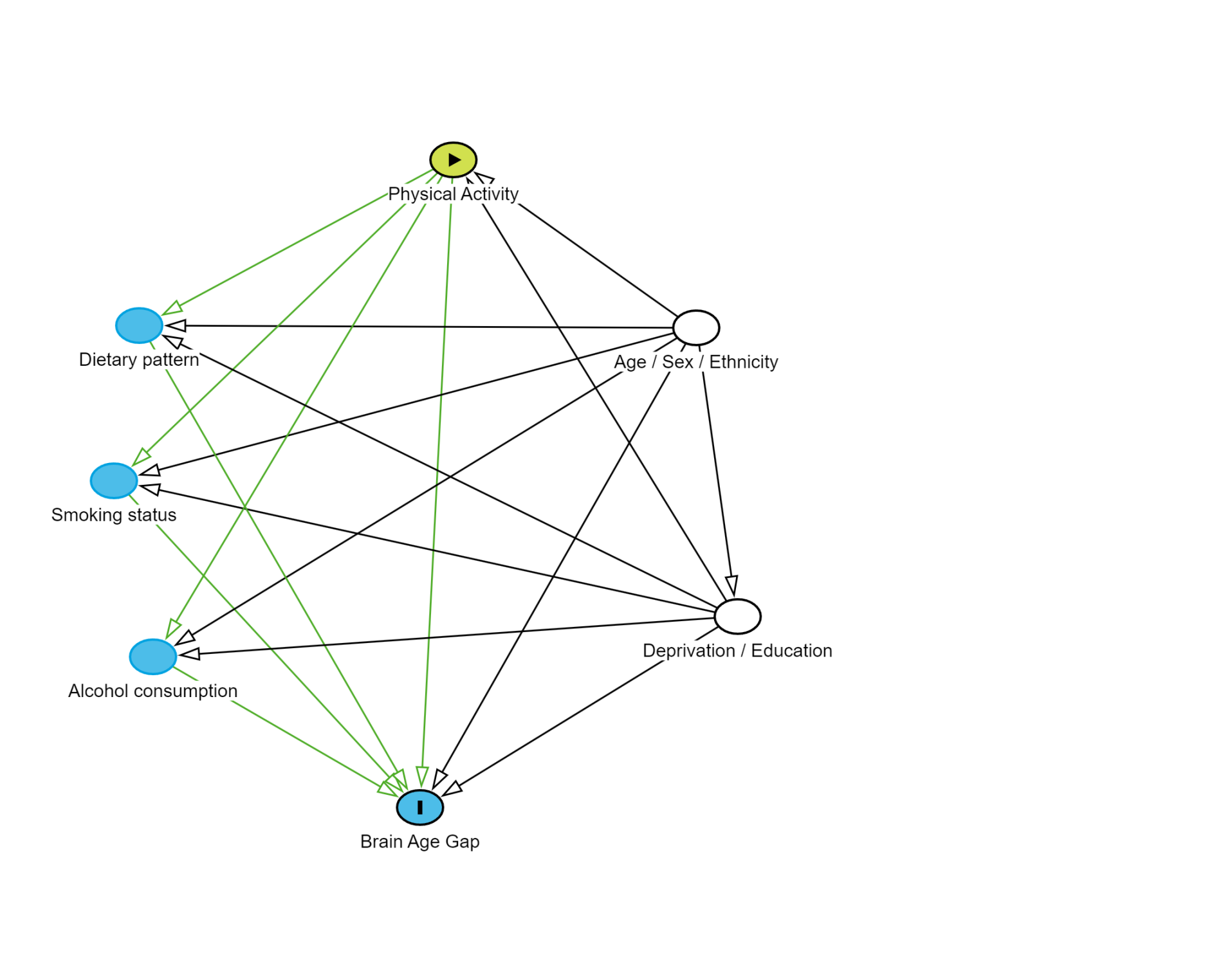
**

The directed acyclic graph to guide covariate selection.

**Fig. S5. Timeline of study design**

**
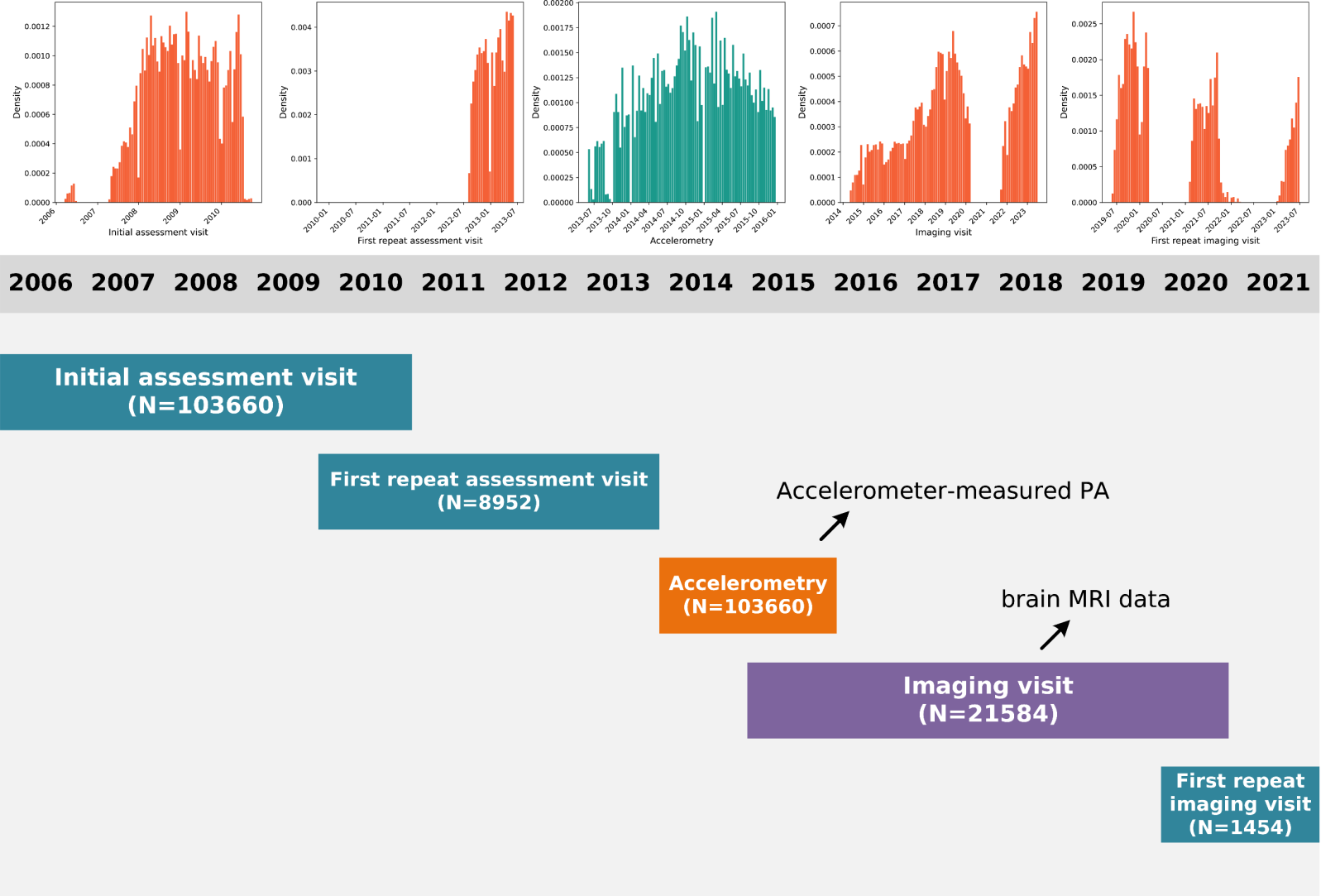
**

The covariates with repeated measurements (educational attainment, dietary pattern, smoking status, alcohol intake frequency, longstanding illness, diabetes, hypertension, and cancer history) were obtained from touchscreen questionnaires at the time-point closest to the accelerometry.

**Table S1. Hyperparameter search space and final adopted hyperparameters**

| **Hyperparameters** | **Search space** | **Final choice** |
| --- | --- | --- |
| num_leaves | lograndint[8, 2^10] | 13 |
| min_data_in_leaf | lograndint[2, 2^7+1] | 4 |
| max_depth | randint[3, 10] | 5 |
| bagging_fraction | uniform[0.5, 1.0] | 0.785010121 |
| bagging_freq | randint[3, 5] | 4 |
| learning_rate | loguniform[1 / 1024, 1.0] | 0.165278078 |
| n_estimators | lograndint[100, 1000] | 760 |
| max_bin | lograndint[2^3, 2^11] | 25 |
| feature_fraction | uniform[0.5, 1.0] | 0.871757489 |
| reg_alpha | loguniform[1 / 1024, 1024] | 0.001155212 |
| reg_lambda | loguniform[1 / 1024, 1024] | 390.0019417 |

Note: LightGBM regressor was fine-tuned with FLAML framework using blended search strategy. Detailed parameters information and search space can be found at <https://microsoft.github.io/FLAML/>, <https://docs.ray.io/en/latest/tune/index.html> and <https://lightgbm.readthedocs.io/en/latest/Parameters.html>

**Table S2. Sequential forward selection of top 27 features**

| **FieldID** | **Features** | **Category** | **MAE (SD)** | ***R*^2^ (SD)** | **RMSE (SD)** | **Permutation importance (SD)** |
| --- | --- | --- | --- | --- | --- | --- |
| f.26528.2.0 | Volume of WM-hypointensities (whole brain) | Freesurfer ASEG | 5.332 (0.068) | 0.286 (0.009) | 0.081 (0.049) | 0.417 (0.049) |
| f.26548.2.0 | Mean intensity of Accumbens-area (left hemisphere) | Freesurfer ASEG | 5.045 (0.064) | 0.355 (0.007) | 0.069 (0.036) | 0.358 (0.036) |
| f.26717.2.0 | Volume of Pons (whole brain) | Freesurfer subsegmentation | 5.042 (0.072) | 0.355 (0.009) | 0.078 (0.043) | 0.337 (0.043) |
| f.26573.2.0 | Mean intensity of Thalamus-Proper (right hemisphere) | Freesurfer ASEG | 4.700 (0.068) | 0.433 (0.010) | 0.078 (0.040) | 0.332 (0.040) |
| f.26688.2.0 | Volume of LGN (right hemisphere) | Freesurfer subsegmentation | 4.431 (0.056) | 0.491 (0.007) | 0.064 (0.027) | 0.146 (0.027) |
| f.27051.2.0 | Grey-white contrast in superiorfrontal (right hemisphere) | Freesurfer desikan gw | 4.281 (0.049) | 0.523 (0.006) | 0.056 (0.032) | 0.144 (0.032) |
| f.25882.2.0 | Volume of grey matter in Putamen (left) | Regional grey matter volumes (FAST) | 4.229 (0.049) | 0.535 (0.007) | 0.055 (0.034) | 0.140 (0.034) |
| f.26545.2.0 | Mean intensity of Pallidum (left hemisphere) | Freesurfer ASEG | 4.176 (0.039) | 0.544 (0.006) | 0.047 (0.027) | 0.133 (0.027) |
| f.25892.2.0 | Volume of grey matter in Brain-Stem | Regional grey matter volumes (FAST) | 4.016 (0.034) | 0.579 (0.004) | 0.045 (0.024) | 0.127 (0.024) |
| f.26683.2.0 | Volume of Pt (left hemisphere) | Freesurfer subsegmentation | 4.000 (0.036) | 0.582 (0.004) | 0.042 (0.024) | 0.109 (0.024) |
| f.26586.2.0 | Volume of Inf-Lat-Vent (right hemisphere) | Freesurfer ASEG | 3.951 (0.036) | 0.590 (0.004) | 0.040 (0.024) | 0.107 (0.024) |
| f.27047.2.0 | Grey-white contrast in precentral (right hemisphere) | Freesurfer desikan gw | 3.943 (0.034) | 0.592 (0.004) | 0.038 (0.027) | 0.105 (0.027) |
| f.25837.2.0 | Volume of grey matter in Paracingulate Gyrus (right) | Regional grey matter volumes (FAST) | 3.831 (0.038) | 0.613 (0.006) | 0.039 (0.022) | 0.100 (0.022) |
| f.26564.2.0 | Volume of Accumbens-area (left hemisphere) | Freesurfer ASEG | 3.806 (0.036) | 0.619 (0.006) | 0.041 (0.024) | 0.093 (0.024) |
| f.26536.2.0 | Volume-ratio of BrainSegVol-to-eTIV (whole brain) | Freesurfer ASEG | 3.776 (0.037) | 0.625 (0.005) | 0.045 (0.023) | 0.093 (0.023) |
| f.26666.2.0 | Volume of PuI (left hemisphere) | Freesurfer subsegmentation | 3.750 (0.040) | 0.629 (0.006) | 0.048 (0.023) | 0.089 (0.023) |
| f.27199.2.0 | Mean thickness of superiorfrontal (left hemisphere) | Freesurfer DKT | 3.716 (0.037) | 0.635 (0.005) | 0.039 (0.024) | 0.083 (0.024) |
| f.25902.2.0 | Volume of grey matter in Crus I Cerebellum (right) | Regional grey matter volumes (FAST) | 3.686 (0.041) | 0.642 (0.006) | 0.048 (0.022) | 0.082 (0.022) |
| f.25846.2.0 | Volume of grey matter in Frontal Orbital Cortex (left) | Regional grey matter volumes (FAST) | 3.661 (0.036) | 0.646 (0.005) | 0.037 (0.019) | 0.060 (0.019) |
| f.25869.2.0 | Volume of grey matter in Planum Polare (right) | Regional grey matter volumes (FAST) | 3.654 (0.041) | 0.648 (0.005) | 0.040 (0.018) | 0.051 (0.018) |
| f.26638.2.0 | Volume of HATA (left hemisphere) | Freesurfer subsegmentation | 3.646 (0.035) | 0.650 (0.005) | 0.038 (0.020) | 0.048 (0.020) |
| f.25920.2.0 | Volume of grey matter in X Cerebellum (right) | Regional grey matter volumes (FAST) | 3.631 (0.040) | 0.652 (0.004) | 0.043 (0.020) | 0.048 (0.020) |
| f.25871.2.0 | Volume of grey matter in Heschl's Gyrus (includes H1 and H2) (right) | Regional grey matter volumes (FAST) | 3.632 (0.035) | 0.652 (0.005) | 0.036 (0.016) | 0.046 (0.016) |
| f.26537.2.0 | Volume-ratio of MaskVol-to-eTIV (whole brain) | Freesurfer ASEG | 3.623 (0.047) | 0.653 (0.005) | 0.043 (0.022) | 0.045 (0.022) |
| f.26646.2.0 | Volume of hippocampal-fissure (right hemisphere) | Freesurfer subsegmentation | 3.615 (0.037) | 0.655 (0.004) | 0.041 (0.023) | 0.044 (0.023) |
| f.26658.2.0 | Volume of fimbria (right hemisphere) | Freesurfer subsegmentation | 3.614 (0.038) | 0.656 (0.004) | 0.037 (0.017) | 0.044 (0.017) |
| f.26527.2.0 | Volume of CSF (whole brain) | Freesurfer ASEG | 3.611 (0.040) | 0.656 (0.004) | 0.043 (0.021) | 0.037 (0.021) |

Note: This step was after redundancy was handled and ranking order was re-prioritized by permutation importance. Permutation importance was calculated based on validation set within ten-fold cross-validation, and performance metrics (MAE, *R*^2^ and RMSE) were also averaged from ten-fold cross-validation results. Detailed information about feature evaluation and performance metrics can be found at https://scikit-learn.org/.

**Table S3. Nonlinear associations between PA and BAG across different threshold settings**

| **Threshold** | **Exposure** | **Outcome** | **EDF** | **F-statistic** | ***P*** |
| --- | --- | --- | --- | --- | --- |
| 0.5 | LPA | BAG | 2.277634 | 1.274748 | 0.345872 |
|  | MPA | BAG | 6.647501 | 6.92626 | 0 |
|  | VPA | BAG | 6.607078 | 5.765683 | 1.04E-07 |
|  | MVPA | BAG | 6.748047 | 7.234649 | 0 |
| 0.75 | LPA | BAG | 3.267195 | 2.473168 | 0.041585 |
|  | MPA | BAG | 6.274474 | 6.488485 | 0 |
|  | VPA | BAG | 5.763165 | 4.924382 | 2.58E-05 |
|  | MVPA | BAG | 6.098688 | 6.453597 | 0 |
| 0.9 | LPA | BAG | 3.024643 | 2.783114 | 0.033287 |
|  | MPA | BAG | 6.14984 | 9.610945 | 0 |
|  | VPA | BAG | 4.616864 | 10.4724 | 0 |
|  | MVPA | BAG | 6.301388 | 9.919494 | 0 |
| Without  thresholding | LPA | BAG | 2.515824 | 2.374116 | 0.068304 |
|  | MPA | BAG | 6.024447 | 6.05071 | 6.89E-07 |
|  | VPA | BAG | 4.911486 | 4.833912 | 9.79E-05 |
|  | MVPA | BAG | 6.33285 | 6.239776 | 0 |

**Table S4. Codes used for brain disorders diagnosis and classification in the UKB**

| **Brain disorders** | **First occurrences** | | | **Algorithmically-defined** | |
| --- | --- | --- | --- | --- | --- |
|  | **FieldID** | **ICD-10 codes** | **Description** | **FieldID** | **Description** |
| Dementia | 130836 | F00 | Date F00 first reported (dementia in alzheimer’s disease) | 42018 | Date of all cause dementia report |
|  | 130838 | F01 | Date F01 first reported (vascular dementia) | 42020 | Date of alzheimer’s disease report |
|  | 130840 | F02 | Date F02 first reported (dementia in other diseases classified elsewhere) | 42022 | Date of vascular dementia report |
|  | 130842 | F03 | Date F03 first reported (unspecified dementia) | 42024 | Date of frontotemporal dementia report |
|  | 131036 | G30 | Date G30 first reported (alzheimer’s disease) | - | - |
| Parkinson’s disease | 131022 | G20 | Date G20 first reported (parkinson’s disease) | 42030 | Date of all cause parkinsonism report |
|  | - | - | - | 42032 | Date of parkinson’s disease report |
|  | - | - | - | 42034 | Date of progressive supranuclear palsy report |
|  | - | - | - | 42036 | Date of multiple system atrophy report |
| Stroke | 131056 | G45 | Date G45 first reported (transient cerebral ischaemic attacks and related syndromes) | 42006 | Date of stroke |
|  | 131058 | G46 | Date G46 first reported (vascular syndromes of brain in cerebrovascular diseases) | 42008 | Date of ischaemic stroke |
|  | 131360 | G47 | Date G47 first reported (sleep disorders) | 42010 | Date of intracerebral haemorrhage |
|  | 131362 | G50 | Date G50 first reported (disorders of trigeminal nerve) | 42012 | Date of subarachnoid haemorrhage |
|  | 131366 | G52 | Date G52 first reported (disorders of other cranial nerves) | - | - |
|  | 131368 | G53 | Date G53 first reported (cranial nerve disorders in diseases classified elsewhere) | - | - |
| Depressive disorder | 130894 | F32 | Date F32 first reported (depressive episode) | - | - |
|  | 130896 | F33 | Date F33 first reported (recurrent depressive disorder) | - | - |
| Anxiety disorder | 130904 | F40 | Date F40 first reported (phobic anxiety disorders) | - | - |
|  | 130906 | F41 | Date F41 first reported (other anxiety disorders) | - | - |
| Bipolar affective disorder | 130892 | F31 | Date F31 first reported (bipolar affective disorder) | - | - |

Note: First occurrences and algorithmically-defined outcomes holds classification of selected health-related events, obtained through hospital admission records, death register, primary care and self-reported data. Participants were followed up until the date of earliest diagnosis, death, or last available censorship date (September 2021), whichever came first. Please refer to the webpage of UK Biobank for detailed information (https://www.ukbiobank.ac.uk).

**Table S5. Codes used for covariates in the UKB**

| **Covariates** | **Touchscreen questionnaire** | | **ICD-10 codes** | **Accelerometer** |
| --- | --- | --- | --- | --- |
|  | **Initial assessment** | **Repeated assessments** |  |  |
| Age at accelerometry | FieldID 34, 52 | - | - | FieldID 90010 |
| Sex | FieldID 31 | - | - | - |
| Ethnicity | FieldID 21000 | - | - | - |
| Townsend deprivation index | FieldID 189 | - | - | - |
| Educational attainment | FieldID 6138 | FieldID 6138 | - | - |
| Dietary pattern | FieldID 1289, 1299, 1309, 1319, 1329, 1339, 1349, 1369, 1379, 1389, 1438, 1448, 1458, 1468 | FieldID 1289, 1299, 1309, 1319, 1329, 1339, 1349, 1369, 1379, 1389, 1438, 1448, 1458, 1468 | - | - |
| Smoking status | FieldID 20116 | FieldID 20116 | - | - |
| Alcohol consumption | FieldID 1558 | FieldID 1558 | - | - |

**Table S6. Baseline characteristics** **according to quartiles of LPA, MPA, VPA, and MVPA**

**Table S6a. Baseline characteristics** **according to quartiles of LPA**

| Characteristics | Total  (*N* = 16,972) | LPA | | | | *P* value |
| --- | --- | --- | --- | --- | --- | --- |
|  |  | Q1  (*n* = 4,352) | Q2  (*n* = 4,345) | Q3  (*n* = 4,096) | Q4  (*n* = 4,179) |  |
| Age, years | 61.7 (7.6) | 61.9 (7.7) | 61.6 (7.6) | 61.5 (7.5) | 61.7 (7.6) | 0.1 |
| Sex |  |  |  |  |  | <0.001 |
| Female | 9,377 (55.2) | 1,782 (40.9) | 2,327 (53.6) | 2,447 (59.7) | 2,821 (67.5) |  |
| Male | 7,595 (44.8) | 2,570 (59.1) | 2,018 (46.4) | 1,649 (40.3) | 1,358 (32.5) |  |
| Townsend deprivation index | -1.9 (2.7) | -1.7 (2.8) | -1.9 (2.7) | -2.0 (2.7) | -2.0 (2.7) | <0.001 |
| Ethnicity |  |  |  |  |  | 0.8 |
| Non-White | 411 (2.4) | 107 (2.5) | 100 (2.3) | 95 (2.3) | 109 (2.6) |  |
| White | 16,561 (97.6) | 4,245 (97.5) | 4,245 (97.7) | 4,001 (97.7) | 4,070 (97.4) |  |
| Educational attainment |  |  |  |  |  | <0.001 |
| Higher education | 8,524 (50.2) | 2,281 (52.4) | 2,236 (51.5) | 2,024 (49.4) | 1,983 (47.5) |  |
| No qualification | 919 (5.4) | 233 (5.4) | 222 (5.1) | 206 (5.0) | 258 (6.2) |  |
| Any other qualification | 7,529 (44.4) | 1,838 (42.2) | 1,887 (43.4) | 1,866 (45.6) | 1,938 (46.4) |  |
| Smoking status |  |  |  |  |  | 0.5 |
| Never smoked | 10,662 (62.8) | 2,702 (62.1) | 2,725 (62.7) | 2,590 (63.2) | 2,645 (63.3) |  |
| Previous smoker | 5,735 (33.8) | 1,487 (34.2) | 1,479 (34.0) | 1,383 (33.8) | 1,386 (33.2) |  |
| Current smoker | 575 (3.4) | 163 (3.7) | 141 (3.2) | 123 (3.0) | 148 (3.5) |  |
| Alcohol intake frequency |  |  |  |  |  | 0.3 |
| Daily or almost daily | 3,094 (18.2) | 801 (18.4) | 748 (17.2) | 761 (18.6) | 784 (18.8) |  |
| Three or four times a week | 4,739 (27.9) | 1,162 (26.7) | 1,252 (28.8) | 1,155 (28.2) | 1,170 (28.0) |  |
| Once or twice a week | 4,380 (25.8) | 1,175 (27.0) | 1,125 (25.9) | 1,045 (25.5) | 1,035 (24.8) |  |
| One to three times a month | 1,985 (11.7) | 512 (11.8) | 516 (11.9) | 490 (12.0) | 467 (11.2) |  |
| Special occasions only | 1,773 (10.4) | 451 (10.4) | 451 (10.4) | 419 (10.2) | 452 (10.8) |  |
| Never | 1,001 (5.9) | 251 (5.8) | 253 (5.8) | 226 (5.5) | 271 (6.5) |  |
| Dietary pattern |  |  |  |  |  | <0.001 |
| Healthy | 10,237 (60.3) | 2,357 (54.2) | 2,605 (60.0) | 2,569 (62.7) | 2,706 (64.8) |  |
| Unhealthy | 6,735 (39.7) | 1,995 (45.8) | 1,740 (40.0) | 1,527 (37.3) | 1,473 (35.2) |  |

Data are n (%) or mean (SD). *P* values were calculated based on the one-way ANOVA for continuous variables, and Pearson’s Chi-square tests for categorical variables.

**Table S6b. Baseline characteristics** **according to quartiles of MPA**

| Characteristics | Total  (*N* = 16,972) | MPA | | | | *P* value |
| --- | --- | --- | --- | --- | --- | --- |
|  |  | Q1  (*n* = 4,285) | Q2  (*n* = 4,263) | Q3  (*n* = 4,253) | Q4  (*n* = 4,171) |  |
| Age, years | 61.7 (7.6) | 64.1 (7.2) | 62.1 (7.7) | 60.7 (7.5) | 59.7 (7.3) | <0.001 |
| Sex |  |  |  |  |  | <0.001 |
| Female | 9,377 (55.2) | 2,197 (51.3) | 2,337 (54.8) | 2,362 (55.5) | 2,481 (59.5) |  |
| Male | 7,595 (44.8) | 2,088 (48.7) | 1,926 (45.2) | 1,891 (44.5) | 1,690 (40.5) |  |
| Townsend deprivation index | -1.9 (2.7) | -1.9 (2.7) | -2.0 (2.7) | -1.9 (2.7) | -1.8 (2.7) | 0.009 |
| Ethnicity |  |  |  |  |  | 0.3 |
| Non-White | 411 (2.4) | 95 (2.2) | 119 (2.8) | 103 (2.4) | 94 (2.3) |  |
| White | 16,561 (97.6) | 4,190 (97.8) | 4,144 (97.2) | 4,150 (97.6) | 4,077 (97.7) |  |
| Educational attainment |  |  |  |  |  | <0.001 |
| Higher education | 8,524 (50.2) | 1,992 (46.5) | 2,183 (51.2) | 2,205 (51.8) | 2,144 (51.4) |  |
| No qualification | 919 (5.4) | 279 (6.5) | 233 (5.5) | 197 (4.6) | 210 (5.0) |  |
| Any other qualification | 7,529 (44.4) | 2,014 (47.0) | 1,847 (43.3) | 1,851 (43.5) | 1,817 (43.6) |  |
| Smoking status |  |  |  |  |  | <0.001 |
| Never smoked | 10,662 (62.8) | 2,569 (60.0) | 2,689 (63.1) | 2,720 (64.0) | 2,684 (64.3) |  |
| Previous smoker | 5,735 (33.8) | 1,544 (36.0) | 1,438 (33.7) | 1,401 (32.9) | 1,352 (32.4) |  |
| Current smoker | 575 (3.4) | 172 (4.0) | 136 (3.2) | 132 (3.1) | 135 (3.2) |  |
| Alcohol intake frequency |  |  |  |  |  | <0.001 |
| Daily or almost daily | 3,094 (18.2) | 778 (18.2) | 782 (18.3) | 773 (18.2) | 761 (18.2) |  |
| Three or four times a week | 4,739 (27.9) | 1,021 (23.8) | 1,178 (27.6) | 1,239 (29.1) | 1,301 (31.2) |  |
| Once or twice a week | 4,380 (25.8) | 1,047 (24.4) | 1,133 (26.6) | 1,120 (26.3) | 1,080 (25.9) |  |
| One to three times a month | 1,985 (11.7) | 563 (13.1) | 489 (11.5) | 489 (11.5) | 444 (10.6) |  |
| Special occasions only | 1,773 (10.4) | 559 (13.0) | 449 (10.5) | 399 (9.4) | 366 (8.8) |  |
| Never | 1,001 (5.9) | 317 (7.4) | 232 (5.4) | 233 (5.5) | 219 (5.3) |  |
| Dietary pattern |  |  |  |  |  | <0.001 |
| Healthy | 10,237 (60.3) | 2,375 (55.4) | 2,509 (58.9) | 2,674 (62.9) | 2,679 (64.2) |  |
| Unhealthy | 6,735 (39.7) | 1,910 (44.6) | 1,754 (41.1) | 1,579 (37.1) | 1,492 (35.8) |  |

Data are n (%) or mean (SD). *P* values were calculated based on the one-way ANOVA for continuous variables, and Pearson’s Chi-square tests for categorical variables.

**Table S6c. Baseline characteristics** **according to quartiles of VPA**

| Characteristics | Total  (*N* = 16,972) | VPA | | | | *P* value |
| --- | --- | --- | --- | --- | --- | --- |
|  |  | Q1  (*n* = 6,627) | Q2  (*n* = 3,203) | Q3  (*n* = 3,348) | Q4  (*n* = 3,794) |  |
| Age, years | 61.7 (7.6) | 63.4 (7.4) | 62.0 (7.4) | 61.0 (7.5) | 59.0 (7.3) | <0.001 |
| Sex |  |  |  |  |  | <0.001 |
| Female | 9,377 (55.2) | 4,033 (60.9) | 1,739 (54.3) | 1,742 (52.0) | 1,863 (49.1) |  |
| Male | 7,595 (44.8) | 2,594 (39.1) | 1,464 (45.7) | 1,606 (48.0) | 1,931 (50.9) |  |
| Townsend deprivation index | -1.9 (2.7) | -1.8 (2.8) | -1.9 (2.7) | -2.0 (2.6) | -1.9 (2.7) | 0.001 |
| Ethnicity |  |  |  |  |  | 0.044 |
| Non-White | 411 (2.4) | 142 (2.1) | 72 (2.2) | 83 (2.5) | 114 (3.0) |  |
| White | 16,561 (97.6) | 6,485 (97.9) | 3,131 (97.8) | 3,265 (97.5) | 3,680 (97.0) |  |
| Educational attainment |  |  |  |  |  | 0.001 |
| Higher education | 8,524 (50.2) | 3,275 (49.4) | 1,561 (48.7) | 1,671 (49.9) | 2,017 (53.2) |  |
| No qualification | 919 (5.4) | 386 (5.8) | 184 (5.7) | 171 (5.1) | 178 (4.7) |  |
| Any other qualification | 7,529 (44.4) | 2,966 (44.8) | 1,458 (45.5) | 1,506 (45.0) | 1,599 (42.1) |  |
| Smoking status |  |  |  |  |  | <0.001 |
| Never smoked | 10,662 (62.8) | 4,076 (61.5) | 1,999 (62.4) | 2,100 (62.7) | 2,487 (65.6) |  |
| Previous smoker | 5,735 (33.8) | 2,289 (34.5) | 1,101 (34.4) | 1,144 (34.2) | 1,201 (31.7) |  |
| Current smoker | 575 (3.4) | 262 (4.0) | 103 (3.2) | 104 (3.1) | 106 (2.8) |  |
| Alcohol intake frequency |  |  |  |  |  | <0.001 |
| Daily or almost daily | 3,094 (18.2) | 1,163 (17.5) | 615 (19.2) | 649 (19.4) | 667 (17.6) |  |
| Three or four times a week | 4,739 (27.9) | 1,639 (24.7) | 906 (28.3) | 1,007 (30.1) | 1,187 (31.3) |  |
| Once or twice a week | 4,380 (25.8) | 1,666 (25.1) | 806 (25.2) | 879 (26.3) | 1,029 (27.1) |  |
| One to three times a month | 1,985 (11.7) | 834 (12.6) | 368 (11.5) | 358 (10.7) | 425 (11.2) |  |
| Special occasions only | 1,773 (10.4) | 838 (12.6) | 325 (10.1) | 309 (9.2) | 301 (7.9) |  |
| Never | 1,001 (5.9) | 487 (7.3) | 183 (5.7) | 146 (4.4) | 185 (4.9) |  |
| Dietary pattern |  |  |  |  |  | <0.001 |
| Healthy | 10,237 (60.3) | 3,890 (58.7) | 1,913 (59.7) | 2,032 (60.7) | 2,402 (63.3) |  |
| Unhealthy | 6,735 (39.7) | 2,737 (41.3) | 1,290 (40.3) | 1,316 (39.3) | 1,392 (36.7) |  |

Data are n (%) or mean (SD). *P* values were calculated based on the one-way ANOVA for continuous variables, and Pearson’s Chi-square tests for categorical variables.

**Table S6d. Baseline characteristics** **according to quartiles of MVPA**

| Characteristics | Total  (*N* = 16,972) | MVPA | | | | *P* value |
| --- | --- | --- | --- | --- | --- | --- |
|  |  | Q1  (*n* = 4,376) | Q2  (*n* = 4,209) | Q3  (*n* = 4,326) | Q4  (*n* = 4,061) |  |
| Age, years | 61.7 (7.6) | 64.2 (7.2) | 62.2 (7.6) | 60.7 (7.5) | 59.5 (7.2) | <0.001 |
| Sex |  |  |  |  |  | <0.001 |
| Female | 9,377 (55.2) | 2,283 (52.2) | 2,325 (55.2) | 2,401 (55.5) | 2,368 (58.3) |  |
| Male | 7,595 (44.8) | 2,093 (47.8) | 1,884 (44.8) | 1,925 (44.5) | 1,693 (41.7) |  |
| Townsend deprivation index | -1.9 (2.7) | -1.9 (2.7) | -2.0 (2.7) | -1.9 (2.7) | -1.8 (2.7) | 0.017 |
| Ethnicity |  |  |  |  |  | 0.3 |
| Non-White | 411 (2.4) | 98 (2.2) | 118 (2.8) | 100 (2.3) | 95 (2.3) |  |
| White | 16,561 (97.6) | 4,278 (97.8) | 4,091 (97.2) | 4,226 (97.7) | 3,966 (97.7) |  |
| Educational attainment |  |  |  |  |  | <0.001 |
| Higher education | 8,524 (50.2) | 2,037 (46.5) | 2,138 (50.8) | 2,261 (52.3) | 2,088 (51.4) |  |
| No qualification | 919 (5.4) | 287 (6.6) | 224 (5.3) | 216 (5.0) | 192 (4.7) |  |
| Any other qualification | 7,529 (44.4) | 2,052 (46.9) | 1,847 (43.9) | 1,849 (42.7) | 1,781 (43.9) |  |
| Smoking status |  |  |  |  |  | <0.001 |
| Never smoked | 10,662 (62.8) | 2,630 (60.1) | 2,637 (62.7) | 2,788 (64.4) | 2,607 (64.2) |  |
| Previous smoker | 5,735 (33.8) | 1,569 (35.9) | 1,434 (34.1) | 1,410 (32.6) | 1,322 (32.6) |  |
| Current smoker | 575 (3.4) | 177 (4.0) | 138 (3.3) | 128 (3.0) | 132 (3.3) |  |
| Alcohol intake frequency |  |  |  |  |  | <0.001 |
| Daily or almost daily | 3,094 (18.2) | 790 (18.1) | 774 (18.4) | 791 (18.3) | 739 (18.2) |  |
| Three or four times a week | 4,739 (27.9) | 1,042 (23.8) | 1,161 (27.6) | 1,253 (29.0) | 1,283 (31.6) |  |
| Once or twice a week | 4,380 (25.8) | 1,064 (24.3) | 1,128 (26.8) | 1,119 (25.9) | 1,069 (26.3) |  |
| One to three times a month | 1,985 (11.7) | 572 (13.1) | 490 (11.6) | 505 (11.7) | 418 (10.3) |  |
| Special occasions only | 1,773 (10.4) | 578 (13.2) | 439 (10.4) | 408 (9.4) | 348 (8.6) |  |
| Never | 1,001 (5.9) | 330 (7.5) | 217 (5.2) | 250 (5.8) | 204 (5.0) |  |
| Dietary pattern |  |  |  |  |  | <0.001 |
| Healthy | 10,237 (60.3) | 2,421 (55.3) | 2,499 (59.4) | 2,694 (62.3) | 2,623 (64.6) |  |
| Unhealthy | 6,735 (39.7) | 1,955 (44.7) | 1,710 (40.6) | 1,632 (37.7) | 1,438 (35.4) |  |

Data are n (%) or mean (SD). *P* values were calculated based on the one-way ANOVA for continuous variables, and Pearson’s Chi-square tests for categorical variables.

**Table S7. Tree-based feature importance ranking of top-100 IDPs**

| **Rank** | **FieldID** | **Features** | **Importance (%)** |
| --- | --- | --- | --- |
| 1 | f.26528.2.0 | Volume of WM-hypointensities (whole brain) | 0.184612655 |
| 2 | f.27051.2.0 | Grey-white contrast in superiorfrontal (right hemisphere) | 0.146681989 |
| 3 | f.26501.2.0 | Mean intensity of 3rd-Ventricle (whole brain) | 0.060924637 |
| 4 | f.26564.2.0 | Volume of Accumbens-area (left hemisphere) | 0.023631513 |
| 5 | f.26688.2.0 | Volume of LGN (right hemisphere) | 0.022816502 |
| 6 | f.26548.2.0 | Mean intensity of Accumbens-area (left hemisphere) | 0.018540974 |
| 7 | f.26573.2.0 | Mean intensity of Thalamus-Proper (right hemisphere) | 0.01737291 |
| 8 | f.26598.2.0 | Volume of choroid-plexus (right hemisphere) | 0.013487238 |
| 9 | f.27037.2.0 | Grey-white contrast in medialorbitofrontal (right hemisphere) | 0.013437041 |
| 10 | f.26717.2.0 | Volume of Pons (whole brain) | 0.013350078 |
| 11 | f.25892.2.0 | Volume of grey matter in Brain-Stem | 0.012952031 |
| 12 | f.26545.2.0 | Mean intensity of Pallidum (left hemisphere) | 0.012829687 |
| 13 | f.26523.2.0 | Volume of 3rd-Ventricle (whole brain) | 0.012651377 |
| 14 | f.26558.2.0 | Volume of Thalamus-Proper (left hemisphere) | 0.011045553 |
| 15 | f.26641.2.0 | Volume of Whole-hippocampus (left hemisphere) | 0.010475681 |
| 16 | f.25837.2.0 | Volume of grey matter in Paracingulate Gyrus (right) | 0.009332936 |
| 17 | f.26579.2.0 | Mean intensity of Accumbens-area (right hemisphere) | 0.008445766 |
| 18 | f.25869.2.0 | Volume of grey matter in Planum Polare (right) | 0.007336022 |
| 19 | f.27199.2.0 | Mean thickness of superiorfrontal (left hemisphere) | 0.006981247 |
| 20 | f.25882.2.0 | Volume of grey matter in Putamen (left) | 0.006398327 |
| 21 | f.25871.2.0 | Volume of grey matter in Heschl's Gyrus (includes H1 and H2) (right) | 0.00632027 |
| 22 | f.25902.2.0 | Volume of grey matter in Crus I Cerebellum (right) | 0.006130926 |
| 23 | f.26639.2.0 | Volume of Whole-hippocampal-body (left hemisphere) | 0.005246028 |
| 24 | f.27047.2.0 | Grey-white contrast in precentral (right hemisphere) | 0.005188528 |
| 25 | f.26536.2.0 | Volume-ratio of BrainSegVol-to-eTIV (whole brain) | 0.005116771 |
| 26 | f.26658.2.0 | Volume of fimbria (right hemisphere) | 0.005056566 |
| 27 | f.26542.2.0 | Mean intensity of Thalamus-Proper (left hemisphere) | 0.005010365 |
| 28 | f.26683.2.0 | Volume of Pt (left hemisphere) | 0.004955394 |
| 29 | f.26636.2.0 | Volume of fimbria (left hemisphere) | 0.004881895 |
| 30 | f.26586.2.0 | Volume of Inf-Lat-Vent (right hemisphere) | 0.004817686 |
| 31 | f.26582.2.0 | Mean intensity of choroid-plexus (right hemisphere) | 0.004682086 |
| 32 | f.26576.2.0 | Mean intensity of Pallidum (right hemisphere) | 0.00452754 |
| 33 | f.27016.2.0 | Grey-white contrast in superiorfrontal (left hemisphere) | 0.004393859 |
| 34 | f.26666.2.0 | Volume of PuI (left hemisphere) | 0.004288608 |
| 35 | f.27040.2.0 | Grey-white contrast in paracentral (right hemisphere) | 0.00423323 |
| 36 | f.26646.2.0 | Volume of hippocampal-fissure (right hemisphere) | 0.004121744 |
| 37 | f.25868.2.0 | Volume of grey matter in Planum Polare (left) | 0.004044476 |
| 38 | f.26527.2.0 | Volume of CSF (whole brain) | 0.003715879 |
| 39 | f.26522.2.0 | Volume of VentricleChoroid (whole brain) | 0.003668804 |
| 40 | f.26665.2.0 | Volume of LGN (left hemisphere) | 0.003662928 |
| 41 | f.25846.2.0 | Volume of grey matter in Frontal Orbital Cortex (left) | 0.003550372 |
| 42 | f.26537.2.0 | Volume-ratio of MaskVol-to-eTIV (whole brain) | 0.003485092 |
| 43 | f.25870.2.0 | Volume of grey matter in Heschl's Gyrus (includes H1 and H2) (left) | 0.003308925 |
| 44 | f.27005.2.0 | Grey-white contrast in paracentral (left hemisphere) | 0.003179381 |
| 45 | f.25015.2.0 | Volume of putamen (left) | 0.00288913 |
| 46 | f.25897.2.0 | Volume of grey matter in VI Cerebellum (left) | 0.002679974 |
| 47 | f.26638.2.0 | Volume of HATA (left hemisphere) | 0.002562625 |
| 48 | f.25782.2.0 | Volume of grey matter in Frontal Pole (left) | 0.002508352 |
| 49 | f.25920.2.0 | Volume of grey matter in X Cerebellum (right) | 0.002466703 |
| 50 | f.26543.2.0 | Mean intensity of Caudate (left hemisphere) | 0.00246353 |
| 51 | f.26572.2.0 | Mean intensity of Cerebellum-Cortex (right hemisphere) | 0.002460289 |
| 52 | f.26690.2.0 | Volume of PuI (right hemisphere) | 0.002448718 |
| 53 | f.25847.2.0 | Volume of grey matter in Frontal Orbital Cortex (right) | 0.002418371 |
| 54 | f.26531.2.0 | Volume of CC-Posterior (whole brain) | 0.002252218 |
| 55 | f.26661.2.0 | Volume of Whole-hippocampal-body (right hemisphere) | 0.002227027 |
| 56 | f.26718.2.0 | Volume of SCP (whole brain) | 0.002217802 |
| 57 | f.26555.2.0 | Volume of Inf-Lat-Vent (left hemisphere) | 0.002197779 |
| 58 | f.26699.2.0 | Volume of VAmc (right hemisphere) | 0.002162931 |
| 59 | f.26567.2.0 | Volume of choroid-plexus (left hemisphere) | 0.002112831 |
| 60 | f.26709.2.0 | Volume of Pt (right hemisphere) | 0.00203957 |
| 61 | f.25783.2.0 | Volume of grey matter in Frontal Pole (right) | 0.00199321 |
| 62 | f.27054.2.0 | Grey-white contrast in supramarginal (right hemisphere) | 0.001977168 |
| 63 | f.25885.2.0 | Volume of grey matter in Pallidum (right) | 0.001926956 |
| 64 | f.27376.2.0 | Area of S-circular-insula-inf (left hemisphere) | 0.001911683 |
| 65 | f.27031.2.0 | Grey-white contrast in inferiorparietal (right hemisphere) | 0.001888945 |
| 66 | f.25883.2.0 | Volume of grey matter in Putamen (right) | 0.001820365 |
| 67 | f.27044.2.0 | Grey-white contrast in pericalcarine (right hemisphere) | 0.00181546 |
| 68 | f.27036.2.0 | Grey-white contrast in lingual (right hemisphere) | 0.00178787 |
| 69 | f.25862.2.0 | Volume of grey matter in Frontal Operculum Cortex (left) | 0.001717317 |
| 70 | f.26587.2.0 | Volume of Cerebellum-White-Matter (right hemisphere) | 0.001693678 |
| 71 | f.26574.2.0 | Mean intensity of Caudate (right hemisphere) | 0.001678627 |
| 72 | f.26585.2.0 | Volume of Lateral-Ventricle (right hemisphere) | 0.001624726 |
| 73 | f.26624.2.0 | Volume of hippocampal-fissure (left hemisphere) | 0.001622034 |
| 74 | f.25863.2.0 | Volume of grey matter in Frontal Operculum Cortex (right) | 0.001595014 |
| 75 | f.26575.2.0 | Mean intensity of Putamen (right hemisphere) | 0.001570724 |
| 76 | f.26551.2.0 | Mean intensity of choroid-plexus (left hemisphere) | 0.001537053 |
| 77 | f.27598.2.0 | Area of S-circular-insula-inf (right hemisphere) | 0.001532826 |
| 78 | f.25830.2.0 | Volume of grey matter in Frontal Medial Cortex (left) | 0.001496345 |
| 79 | f.25792.2.0 | Volume of grey matter in Inferior Frontal Gyrus, pars opercularis (left) | 0.00147603 |
| 80 | f.27103.2.0 | Area of BA3a (right hemisphere) | 0.001466436 |
| 81 | f.25843.2.0 | Volume of grey matter in Precuneous Cortex (right) | 0.001412468 |
| 82 | f.25915.2.0 | Volume of grey matter in IX Cerebellum (left) | 0.00139329 |
| 83 | f.26502.2.0 | Mean intensity of 4th-Ventricle (whole brain) | 0.001364871 |
| 84 | f.26530.2.0 | Volume of Optic-Chiasm (whole brain) | 0.001354066 |
| 85 | f.26513.2.0 | Mean intensity of CC-Anterior (whole brain) | 0.001334991 |
| 86 | f.27004.2.0 | Grey-white contrast in parahippocampal (left hemisphere) | 0.001292542 |
| 87 | f.26509.2.0 | Mean intensity of CC-Posterior (whole brain) | 0.001281146 |
| 88 | f.26588.2.0 | Volume of Cerebellum-Cortex (right hemisphere) | 0.001280308 |
| 89 | f.27013.2.0 | Grey-white contrast in precuneus (left hemisphere) | 0.001273699 |
| 90 | f.26589.2.0 | Volume of Thalamus-Proper (right hemisphere) | 0.001206804 |
| 91 | f.26596.2.0 | Volume of VentralDC (right hemisphere) | 0.001176276 |
| 92 | f.27739.2.0 | Volume of Lat-Fis-post (right hemisphere) | 0.001138313 |
| 93 | f.27019.2.0 | Grey-white contrast in supramarginal (left hemisphere) | 0.001126253 |
| 94 | f.27039.2.0 | Grey-white contrast in parahippocampal (right hemisphere) | 0.001108579 |
| 95 | f.27134.2.0 | Volume of BA4p (right hemisphere) | 0.001106372 |
| 96 | f.26720.2.0 | Volume of Whole-brainstem (whole brain) | 0.001086949 |
| 97 | f.26535.2.0 | Volume of CC-Anterior (whole brain) | 0.001072654 |
| 98 | f.27131.2.0 | Volume of BA3a (right hemisphere) | 0.001056835 |
| 99 | f.25805.2.0 | Volume of grey matter in Middle Temporal Gyrus, posterior division (right) | 0.001036812 |
| 100 | f.26591.2.0 | Volume of Putamen (right hemisphere) | 0.001025784 |

**Table S8. Final model performance before and after brain age correction**

| **Metrics** | **Before correction** | **After correction** |
| --- | --- | --- |
| MAE | 3.654 (95% CI: 3.628-3.680) | 3.027 (95% CI: 3.008-3.048) |
| *R*^2^ | 0.648 (95% CI: 0.643-0.653) | 0.762 (95% CI: 0.758-0.766) |
| RMSE | 4.591 (95% CI: 4.560-4.621) | 3.777 (95% CI: 3.753-3.801) |

Note: 95% confidence intervals were computed by bootstrapping with 5000 replicates

**Table S9. Nonlinear associations between PA and BAG**

| **Exposure** | **Outcome** | **EDF** | **F-statistic** | ***P*** |
| --- | --- | --- | --- | --- |
| LPA | BAG | 3.267195279 | 2.473168665 | 0.041585213 |
| MPA | BAG | 6.274474199 | 6.488485383 | 0 |
| VPA | BAG | 5.76316571 | 4.9243823 | 2.58E-05 |
| MVPA | BAG | 6.098688195 | 6.453597898 | 0 |

Note: Models were adjusted for age, sex, ethnicity, educational attainment, Townsend deprivation index, smoking status, alcohol consumption, and dietary pattern. EDF (effective degrees of freedom) estimated from generalized additive models were used as a proxy for the degree of non-linearity.

**Table S10. The difference in BAG between PA quartiles**

|  | ***N*** | **BAG (SD)** | ***P*** | **SMD** |
| --- | --- | --- | --- | --- |
| Overall | 16972 | -0.05 (3.75) |  |  |
| LPA.Q1 | 4352 | -0.04 (3.83) |  |  |
| LPA.Q2 | 4345 | -0.12 (3.68) | 0.6876 | 0.023 |
| LPA.Q3 | 4096 | -0.11 (3.72) | 1 | 0.018 |
| LPA.Q4 | 4179 | 0.05 (3.75) | 0.8724 | 0.023 |
| MPA.Q1 | 4285 | 0.10 (3.82) |  |  |
| MPA.Q2 | 4263 | -0.10 (3.65) | 0.0126 | 0.053 |
| MPA.Q3 | 4253 | -0.07 (3.71) | 0.0135 | 0.046 |
| MPA.Q4 | 4171 | -0.15 (3.80) | 0.0018 | 0.065 |
| VPA.Q1 | 6627 | 0.06 (3.79) |  |  |
| VPA.Q2 | 3203 | -0.11 (3.73) | 0.0399 | 0.047 |
| VPA.Q3 | 3348 | -0.13 (3.73) | 0.0249 | 0.051 |
| VPA.Q4 | 3794 | -0.14 (3.70) | 0.0123 | 0.053 |
| MVPA.Q1 | 4376 | 0.09 (3.82) |  |  |
| MVPA.Q2 | 4209 | -0.09 (3.65) | 0.0294 | 0.049 |
| MVPA.Q3 | 4326 | -0.08 (3.71) | 0.0147 | 0.046 |
| MVPA.Q4 | 4061 | -0.14 (3.80) | 0.0033 | 0.061 |

Note: BAG was reported as mean (SD). *P* was calculated by Wilcoxon rank-sum test between Q2 vs Q1, Q3 vs Q1 and Q4 vs Q1, respectively, and then adjusted by FDR correction (α=0.05). SMD (standardized mean differences) was calculated between Q2 vs Q1, Q3 vs Q1 and Q4 vs Q1, respectively.

**Table S11. Linear associations between PA quartiles and BAG**

| **Exposure** | **Outcome** | ***N*** | **β (95% CI)** | **SE** | **T-statistic** | ***P*** |
| --- | --- | --- | --- | --- | --- | --- |
| LPA.Q1 | BAG | 4352 | Reference |  |  |  |
| LPA.Q2 | BAG | 4345 | -0.043 (-0.200,0.115) | 0.08055533 | -0.52856234 | 0.597116012 |
| LPA.Q3 | BAG | 4096 | -0.012 (-0.174,0.149) | 0.08229022 | -0.151821475 | 0.879329551 |
| LPA.Q4 | BAG | 4179 | 0.163 (0.001,0.325) | 0.08272629 | 1.974267955 | 0.048367493 |
| MPA.Q1 | BAG | 4285 | Reference |  |  |  |
| MPA.Q2 | BAG | 4263 | -0.203 (-0.363,-0.044) | 0.081460565 | -2.495253772 | 0.012596145 |
| MPA.Q3 | BAG | 4253 | -0.185 (-0.346,-0.023) | 0.082452176 | -2.24026194 | 0.025086828 |
| MPA.Q4 | BAG | 4171 | -0.263 (-0.427,-0.098) | 0.083850661 | -3.133893345 | 0.001727978 |
| VPA.Q1 | BAG | 6627 | Reference |  |  |  |
| VPA.Q2 | BAG | 3203 | -0.212 (-0.370,-0.053) | 0.080874843 | -2.616322528 | 0.008896139 |
| VPA.Q3 | BAG | 3348 | -0.241 (-0.399,-0.084) | 0.080399381 | -3.001519951 | 0.002690266 |
| VPA.Q4 | BAG | 3794 | -0.266 (-0.421,-0.111) | 0.079249213 | -3.356475974 | 7.91E-04 |
| MVPA.Q1 | BAG | 4376 | Reference |  |  |  |
| MVPA.Q2 | BAG | 4209 | -0.188 (-0.347,-0.029) | 0.081292238 | -2.312501 | 0.02076201 |
| MVPA.Q3 | BAG | 4326 | -0.185 (-0.346,-0.025) | 0.081729339 | -2.269005204 | 0.023280525 |
| MVPA.Q4 | BAG | 4061 | -0.256 (-0.422,-0.091) | 0.084285774 | -3.042904678 | 0.002346652 |

Note: Q1-4 represents quartiles of PA, and Q1 was used as reference. Models were adjusted for age, sex, ethnicity, educational attainment, Townsend deprivation index, smoking status, alcohol consumption, and dietary pattern.

**Table S12. Mediation analysis between PA, cognitive function, and BAG**

| **Exposure** | **Mediator** | **Outcome** | ***N*** | **ACME (95% CI)** | **ACME *P*** | **ADE (95% CI)** | **ADE *P*** | **Prop Med (95% CI)** | **Prop Med *P*** |
| --- | --- | --- | --- | --- | --- | --- | --- | --- | --- |
| VPA | BAG | Reaction time | 15957 | -0.0033 (-0.0061,-0.0011) | 0.004 | -0.1045 (-0.1561,-0.0550) | 0 | 3.0113 (0.9582,6.9714) | 0.004 |
| VPA | BAG | Fluid intelligence | 15780 | 0.0001 (0.0000,0.0001) | 0.004 | -0.0003 (-0.0013,0.0005) | 0.5 | -9.4097 (-152.0785,183.1195) | 0.586 |
| VPA | BAG | Numeric memory | 11270 | 0.0000 (-0.0000,0.0001) | 0.558 | 0.0007 (-0.0001,0.0015) | 0.096 | 1.4783 (-13.7069,27.5232) | 0.606 |
| VPA | BAG | Matrix pattern completion | 10899 | 0.0000 (-0.0000,0.0001) | 0.594 | -0.0002 (-0.0012,0.0009) | 0.728 | -0.2491 (-98.1444,76.2537) | 0.98 |
| VPA | BAG | Trail making A | 11010 | -0.0005 (-0.0024,0.0012) | 0.584 | -0.0428 (-0.0869,0.0005) | 0.06 | 1.1452 (-5.9322,12.8329) | 0.588 |
| VPA | BAG | Trail making B | 11010 | -0.0019 (-0.0079,0.0041) | 0.494 | -0.1201 (-0.2558,0.0113) | 0.078 | 1.4343 (-7.6695,15.5346) | 0.528 |
| VPA | BAG | Symbol–digit substitution | 10920 | 0.0001 (-0.0001,0.0002) | 0.576 | 0.0039 (0.0013,0.0063) | 0.002 | 1.3218 (-3.5310,8.0117) | 0.578 |
| VPA | BAG | Pair matching | 16051 | -0.0020 (-0.0037,-0.0006) | 0.002 | 0.0091 (-0.0281,0.0482) | 0.638 | -5.6678 (-176.9746,179.1383) | 0.718 |
| VPA | BAG | Tower rearranging | 10836 | 0.0000 (-0.0000,0.0001) | 0.62 | -0.0017 (-0.0033,0.0001) | 0.076 | -0.5039 (-8.1201,6.9606) | 0.668 |
| VPA | BAG | Prospective memory | 16043 | -0.0000 (-0.0000,0.0000) | 0.21 | 0.0000 (-0.0001,0.0002) | 0.618 | -1.3132 (-48.6284,39.4684) | 0.678 |
| MPA | BAG | Reaction time | 15957 | -0.0004 (-0.0008,0.0000) | 0.06 | -0.0145 (-0.0220,-0.0078) | 0 | 2.4011 (-0.0400,6.3682) | 0.06 |
| MPA | BAG | Fluid intelligence | 15780 | 0.0000 (-0.0000,0.0000) | 0.07 | -0.0002 (-0.0003,-0.0000) | 0.016 | -3.8968 (-17.2982,0.7032) | 0.09 |
| MPA | BAG | Numeric memory | 11270 | 0.0000 (-0.0000,0.0000) | 0.23 | 0.0001 (-0.0000,0.0002) | 0.178 | 3.7121 (-38.7361,59.2254) | 0.354 |
| MPA | BAG | Matrix pattern completion | 10899 | 0.0000 (-0.0000,0.0000) | 0.2 | -0.0003 (-0.0005,-0.0001) | 0 | -2.1876 (-8.3105,1.4189) | 0.2 |
| MPA | BAG | Trail making A | 11010 | -0.0002 (-0.0005,0.0001) | 0.172 | -0.0070 (-0.0144,0.0003) | 0.068 | 2.3856 (-3.8402,20.4290) | 0.224 |
| MPA | BAG | Trail making B | 11010 | -0.0007 (-0.0018,0.0002) | 0.136 | -0.0160 (-0.0374,0.0043) | 0.122 | 3.4922 (-19.3688,37.0644) | 0.218 |
| MPA | BAG | Symbol–digit substitution | 10920 | 0.0000 (-0.0000,0.0000) | 0.212 | 0.0006 (0.0001,0.0010) | 0.008 | 3.1478 (-2.7138,15.2725) | 0.214 |
| MPA | BAG | Pair matching | 16051 | -0.0002 (-0.0005,0.0000) | 0.054 | 0.0020 (-0.0037,0.0081) | 0.488 | -4.6555 (-113.4291,105.9242) | 0.556 |
| MPA | BAG | Tower rearranging | 10836 | 0.0000 (-0.0000,0.0000) | 0.202 | -0.0003 (-0.0005,-0.0000) | 0.046 | -1.5004 (-18.1647,3.2601) | 0.254 |
| MPA | BAG | Prospective memory | 16043 | -0.0000 (-0.0000,0.0000) | 0.286 | 0.0000 (-0.0000,0.0000) | 0.406 | -0.8890 (-16.2405,25.8477) | 0.588 |
| MVPA | BAG | Reaction time | 15957 | -0.0004 (-0.0008,-0.0001) | 0.016 | -0.0148 (-0.0214,-0.0082) | 0 | 2.5033 (0.4323,5.8716) | 0.016 |
| MVPA | BAG | Fluid intelligence | 15780 | 0.0000 (0.0000,0.0000) | 0.024 | -0.0002 (-0.0003,-0.0000) | 0.012 | -4.6407 (-25.1843,-0.1952) | 0.04 |
| MVPA | BAG | Numeric memory | 11270 | 0.0000 (-0.0000,0.0000) | 0.214 | 0.0001 (-0.0000,0.0002) | 0.172 | 3.6556 (-59.6735,32.6263) | 0.34 |
| MVPA | BAG | Matrix pattern completion | 10899 | 0.0000 (-0.0000,0.0000) | 0.214 | -0.0003 (-0.0004,-0.0001) | 0.004 | -2.4329 (-9.1693,1.7209) | 0.214 |
| MVPA | BAG | Trail making A | 11010 | -0.0002 (-0.0005,0.0001) | 0.164 | -0.0071 (-0.0136,-0.0004) | 0.044 | 2.4850 (-2.1611,12.7918) | 0.194 |
| MVPA | BAG | Trail making B | 11010 | -0.0006 (-0.0016,0.0002) | 0.142 | -0.0156 (-0.0355,0.0043) | 0.13 | 3.3468 (-20.9885,37.5909) | 0.236 |
| MVPA | BAG | Symbol–digit substitution | 10920 | 0.0000 (-0.0000,0.0000) | 0.196 | 0.0006 (0.0002,0.0010) | 0.004 | 2.7792 (-1.5166,11.7781) | 0.2 |
| MVPA | BAG | Pair matching | 16051 | -0.0002 (-0.0005,-0.0000) | 0.022 | 0.0021 (-0.0032,0.0075) | 0.412 | -6.1141 (-140.5752,121.8307) | 0.476 |
| MVPA | BAG | Tower rearranging | 10836 | 0.0000 (-0.0000,0.0000) | 0.2 | -0.0003 (-0.0005,-0.0000) | 0.036 | -1.3948 (-10.1425,2.3484) | 0.232 |
| MVPA | BAG | Prospective memory | 16043 | -0.0000 (-0.0000,0.0000) | 0.256 | 0.0000 (-0.0000,0.0000) | 0.404 | -1.0483 (-34.0824,32.2177) | 0.576 |
| LPA | BAG | Reaction time | 15957 | 0.0001 (-0.0000,0.0003) | 0.124 | 0.0005 (-0.0032,0.0043) | 0.77 | 3.0231 (-109.1896,78.8163) | 0.762 |
| LPA | BAG | Fluid intelligence | 15780 | -0.0000 (-0.0000,0.0000) | 0.252 | -0.0003 (-0.0004,-0.0002) | 0 | 0.6971 (-0.4051,1.9506) | 0.252 |
| LPA | BAG | Numeric memory | 11270 | -0.0000 (-0.0000,0.0000) | 0.298 | -0.0001 (-0.0002,-0.0000) | 0.004 | 1.9661 (-2.3172,10.2148) | 0.3 |
| LPA | BAG | Matrix pattern completion | 10899 | -0.0000 (-0.0000,0.0000) | 0.448 | -0.0003 (-0.0004,-0.0002) | 0 | 0.6058 (-0.9425,2.3964) | 0.448 |
| LPA | BAG | Trail making A | 11010 | 0.0001 (-0.0001,0.0002) | 0.458 | 0.0047 (0.0010,0.0083) | 0.014 | 1.0384 (-2.5175,7.6526) | 0.468 |
| LPA | BAG | Trail making B | 11010 | 0.0002 (-0.0003,0.0007) | 0.468 | 0.0219 (0.0107,0.0329) | 0 | 0.6567 (-1.4823,3.3962) | 0.468 |
| LPA | BAG | Symbol–digit substitution | 10920 | -0.0000 (-0.0000,0.0000) | 0.416 | -0.0006 (-0.0007,-0.0003) | 0 | 1.0573 (-1.7899,4.1297) | 0.416 |
| LPA | BAG | Pair matching | 16051 | 0.0001 (-0.0000,0.0002) | 0.132 | 0.0052 (0.0023,0.0083) | 0.004 | 1.4637 (-0.5469,4.8893) | 0.136 |
| LPA | BAG | Tower rearranging | 10836 | -0.0000 (-0.0000,0.0000) | 0.454 | -0.0005 (-0.0006,-0.0003) | 0 | 0.2977 (-0.4857,1.3720) | 0.454 |
| LPA | BAG | Prospective memory | 16043 | 0.0000 (-0.0000,0.0000) | 0.418 | 0.0000 (0.0000,0.0000) | 0.012 | 0.3515 (-0.7554,3.0239) | 0.422 |

Note: Causal mediation was estimated by 1000 Monte Carlo simulations. Models were adjusted for age, sex, ethnicity, educational attainment, Townsend deprivation index, smoking status, alcohol consumption, and dietary pattern. ACME (average causal mediation effects), ADE (average direct effects), Prop Med (proportions mediated, %), and CI (confidence interval). Prop Med was the size of the average causal mediation effects relative to the total effect. *P* represents two-sided p-value.

**Table S13. Mediation analysis between PA, brain disorders, and BAG**

| **Exposure** | **Mediator** | **Outcome** | **Case/N** | **ACME (95% CI)** | **ACME *P*** | **ADE (95% CI)** | **ADE *P*** | **Prop Med (95% CI)** | **Prop Med *P*** |
| --- | --- | --- | --- | --- | --- | --- | --- | --- | --- |
| VPA | BAG | Dementia | 58/16969 | -0.0000 (-0.0000,-0.0000) | 0.014 | -0.0001 (-0.0002,-0.0000) | 0.03 | 2.9640 (0.4943,18.1098) | 0.03 |
| VPA | BAG | Parkinson's disease | 37/16956 | -0.0000 (-0.0000,0.0000) | 0.214 | -0.0001 (-0.0002,-0.0000) | 0.04 | 0.6146 (-0.8987,4.4372) | 0.25 |
| VPA | BAG | Stroke | 250/16657 | -0.0000 (-0.0000,-0.0000) | 0.02 | -0.0000 (-0.0001,0.0000) | 0.228 | 4.3962 (-40.8510,59.3833) | 0.218 |
| VPA | BAG | Depressive disorder | 278/15138 | -0.0000 (-0.0000,0.0000) | 0.09 | -0.0001 (-0.0002,-0.0000) | 0.01 | 1.1725 (-0.1855,7.1408) | 0.096 |
| VPA | BAG | Anxiety disorder | 381/15972 | -0.0000 (-0.0000,0.0000) | 0.894 | -0.0001 (-0.0003,-0.0000) | 0.006 | 0.0525 (-1.3145,1.7580) | 0.892 |
| VPA | BAG | Bipolar affective disorder | 4/16912 | 0.0000 (-0.0000,0.0000) | 0.298 | -0.0000 (-0.0001,0.0000) | 0.63 | -0.8082 (-35.6719,29.5385) | 0.74 |
| MPA | BAG | Dementia | 58/16969 | -0.0000 (-0.0000,-0.0000) | 0.008 | -0.0000 (-0.0000,0.0000) | 0.49 | 7.1074 (-98.1053,111.5840) | 0.442 |
| MPA | BAG | Parkinson's disease | 37/16956 | -0.0000 (-0.0000,0.0000) | 0.246 | -0.0000 (-0.0001,-0.0000) | 0 | 0.5219 (-0.3733,2.4507) | 0.246 |
| MPA | BAG | Stroke | 250/16657 | -0.0000 (-0.0000,-0.0000) | 0.022 | -0.0000 (-0.0000,0.0000) | 0.95 | 0.0728 (-112.4455,138.7801) | 1 |
| MPA | BAG | Depressive disorder | 278/15138 | -0.0000 (-0.0000,-0.0000) | 0.034 | -0.0000 (-0.0001,-0.0000) | 0.002 | 1.1887 (0.0698,3.8928) | 0.034 |
| MPA | BAG | Anxiety disorder | 381/15972 | -0.0000 (-0.0000,0.0000) | 0.816 | -0.0000 (-0.0001,-0.0000) | 0.002 | 0.0839 (-1.2944,1.7257) | 0.812 |
| MPA | BAG | Bipolar affective disorder | 4/16912 | 0.0000 (-0.0000,0.0000) | 0.234 | -0.0001 (-0.0004,-0.0000) | 0.036 | -0.5550 (-3.3798,0.6304) | 0.254 |
| MVPA | BAG | Dementia | 58/16969 | -0.0000 (-0.0000,-0.0000) | 0.014 | -0.0000 (-0.0000,0.0000) | 0.328 | 8.0647 (-84.2423,100.7464) | 0.286 |
| MVPA | BAG | Parkinson's disease | 37/16956 | -0.0000 (-0.0000,0.0000) | 0.204 | -0.0000 (-0.0001,-0.0000) | 0.002 | 0.4948 (-0.2724,2.5954) | 0.206 |
| MVPA | BAG | Stroke | 250/16657 | -0.0000 (-0.0000,-0.0000) | 0.018 | -0.0000 (-0.0000,0.0000) | 0.864 | 4.0924 (-100.4013,110.8835) | 0.812 |
| MVPA | BAG | Depressive disorder | 278/15138 | -0.0000 (-0.0000,-0.0000) | 0.024 | -0.0000 (-0.0001,-0.0000) | 0 | 1.2235 (0.1074,4.0885) | 0.024 |
| MVPA | BAG | Anxiety disorder | 381/15972 | -0.0000 (-0.0000,0.0000) | 0.894 | -0.0000 (-0.0001,-0.0000) | 0 | 0.0480 (-1.3768,1.9098) | 0.894 |
| MVPA | BAG | Bipolar affective disorder | 4/16912 | 0.0000 (-0.0000,0.0000) | 0.268 | -0.0001 (-0.0004,-0.0000) | 0.028 | -0.6216 (-5.6575,0.9889) | 0.294 |
| LPA | BAG | Dementia | 58/16969 | 0.0000 (-0.0000,0.0000) | 0.262 | 0.0000 (-0.0000,0.0000) | 0.514 | 3.4100 (-70.0420,73.7662) | 0.602 |
| LPA | BAG | Parkinson's disease | 37/16956 | 0.0000 (-0.0000,0.0000) | 0.394 | -0.0000 (-0.0001,-0.0000) | 0.006 | -0.3439 (-3.3699,0.6662) | 0.4 |
| LPA | BAG | Stroke | 250/16657 | 0.0000 (-0.0000,0.0000) | 0.244 | -0.0000 (-0.0000,0.0000) | 0.8 | -1.1875 (-72.1988,62.1212) | 0.882 |
| LPA | BAG | Depressive disorder | 278/15138 | 0.0000 (-0.0000,0.0000) | 0.182 | -0.0000 (-0.0000,0.0000) | 0.174 | -1.8470 (-38.8108,14.9465) | 0.34 |
| LPA | BAG | Anxiety disorder | 381/15972 | 0.0000 (-0.0000,0.0000) | 0.846 | -0.0000 (-0.0000,0.0000) | 0.7 | -0.0232 (-11.7617,15.0385) | 0.962 |
| LPA | BAG | Bipolar affective disorder | 4/16912 | -0.0000 (-0.0000,0.0000) | 0.442 | -0.0000 (-0.0001,0.0000) | 0.386 | 0.4394 (-9.7343,13.0490) | 0.65 |

Note: Causal mediation was estimated by 1000 Monte Carlo simulations. Models were adjusted for age, sex, ethnicity, educational attainment, Townsend deprivation index, smoking status, alcohol consumption, and dietary pattern. ACME (average causal mediation effects), ADE (average direct effects), Prop Med (proportions mediated, %), and CI (confidence interval). Prop Med was the size of the average causal mediation effects relative to the total effect. *P* represents two-sided p-value.

**Table S14. Linear associations between PA and 20 selected features**

| **Exposure** | **Outcome** | **Category** | **Features** | **β** | **βstd** | **SE** | **T-**  **statistic** | ***P*** | **CI-low** | **CI-high** | **P-FDR** |
| --- | --- | --- | --- | --- | --- | --- | --- | --- | --- | --- | --- |
| LPA | f.26536.2.0 | Freesurfer ASEG | Volume-ratio of BrainSegVol-to-eTIV (whole brain) | 2.67E-06 | 0.034367791 | 5.86E-07 | 4.564111319 | 5.05E-06 | 1.53E-06 | 3.82E-06 | 1.01E-04 |
| LPA | f.25902.2.0 | Regional grey matter volumes (FAST) | Volume of grey matter in Crus I Cerebellum (right) | 0.072428389 | 0.017590933 | 0.029702944 | 2.4384246 | 0.014761633 | 0.014207531 | 0.130649247 | 0.147616326 |
| LPA | f.25846.2.0 | Regional grey matter volumes (FAST) | Volume of grey matter in Frontal Orbital Cortex (left) | -0.028647551 | -0.01436274 | 0.013759893 | -2.081960284 | 0.037361062 | -0.055618372 | -0.00167673 | 0.18680531 |
| LPA | f.26688.2.0 | Freesurfer subsegmentation | Volume of LGN (right hemisphere) | -0.001031523 | -0.015209956 | 4.78E-04 | -2.158459195 | 0.030906141 | -0.001968252 | -9.48E-05 | 0.18680531 |
| LPA | f.26548.2.0 | Freesurfer ASEG | Mean intensity of Accumbens-area (left hemisphere) | -7.06E-05 | -0.013646667 | 3.87E-05 | -1.825124679 | 0.067999807 | -1.46E-04 | 5.22E-06 | 0.271999227 |
| LPA | f.26545.2.0 | Freesurfer ASEG | Mean intensity of Pallidum (left hemisphere) | -4.58E-05 | -0.011216874 | 3.14E-05 | -1.458113605 | 0.14482776 | -1.07E-04 | 1.58E-05 | 0.450729398 |
| LPA | f.25837.2.0 | Regional grey matter volumes (FAST) | Volume of grey matter in Paracingulate Gyrus (right) | -0.019663578 | -0.009703923 | 0.014477975 | -1.358171799 | 0.174427254 | -0.048041915 | 0.008714759 | 0.450729398 |
| LPA | f.26717.2.0 | Freesurfer subsegmentation | Volume of Pons (whole brain) | 0.041368757 | 0.009345772 | 0.030874223 | 1.33991249 | 0.180291759 | -0.01914793 | 0.101885444 | 0.450729398 |
| LPA | f.26564.2.0 | Freesurfer ASEG | Volume of Accumbens-area (left hemisphere) | -0.001799839 | -0.008546091 | 0.001458143 | -1.234336784 | 0.217094547 | -0.00465795 | 0.001058272 | 0.482432326 |
| LPA | f.25882.2.0 | Regional grey matter volumes (FAST) | Volume of grey matter in Putamen (left) | 0.008326287 | 0.008370583 | 0.0076898 | 1.082770321 | 0.278925785 | -0.00674652 | 0.023399095 | 0.557851569 |
| LPA | f.25892.2.0 | Regional grey matter volumes (FAST) | Volume of grey matter in Brain-Stem | 0.01334967 | 0.007131616 | 0.013240476 | 1.008246992 | 0.313350296 | -0.012603039 | 0.039302378 | 0.569727811 |
| LPA | f.26666.2.0 | Freesurfer subsegmentation | Volume of PuI (left hemisphere) | -3.57E-04 | -0.006789631 | 3.86E-04 | -0.925791217 | 0.354567673 | -0.001113284 | 3.99E-04 | 0.590946122 |
| LPA | f.26573.2.0 | Freesurfer ASEG | Mean intensity of Thalamus-Proper (right hemisphere) | -2.10E-05 | -0.004927184 | 3.08E-05 | -0.683105462 | 0.494549541 | -8.13E-05 | 3.93E-05 | 0.659399388 |
| LPA | f.26586.2.0 | Freesurfer ASEG | Volume of Inf-Lat-Vent (right hemisphere) | 0.003521129 | 0.005245537 | 0.004741996 | 0.742541669 | 0.457769505 | -0.005773675 | 0.012815934 | 0.659399388 |
| LPA | f.27051.2.0 | Freesurfer desikan gw | Grey-white contrast in superiorfrontal (right hemisphere) | 3.07E-05 | 0.005013359 | 4.22E-05 | 0.725781713 | 0.46798262 | -5.21E-05 | 1.13E-04 | 0.659399388 |
| LPA | f.27047.2.0 | Freesurfer desikan gw | Grey-white contrast in precentral (right hemisphere) | 1.22E-05 | 0.002687498 | 3.33E-05 | 0.36691898 | 0.713684034 | -5.31E-05 | 7.76E-05 | 0.892105043 |
| LPA | f.26528.2.0 | Freesurfer ASEG | Volume of WM-hypointensities (whole brain) | -0.012994968 | -0.001953328 | 0.049202757 | -0.26411056 | 0.791697938 | -0.109437486 | 0.083447551 | 0.918774623 |
| LPA | f.25869.2.0 | Regional grey matter volumes (FAST) | Volume of grey matter in Planum Polare (right) | -7.95E-04 | -0.0014824 | 0.003839523 | -0.207099276 | 0.835934815 | -0.008321026 | 0.006730702 | 0.918774623 |
| LPA | f.26683.2.0 | Freesurfer subsegmentation | Volume of Pt (left hemisphere) | -2.27E-06 | -0.001156571 | 1.42E-05 | -0.160059778 | 0.872835892 | -3.01E-05 | 2.55E-05 | 0.918774623 |
| LPA | f.27199.2.0 | Freesurfer DKT | Mean thickness of superiorfrontal (left hemisphere) | 5.24E-08 | 1.39E-04 | 2.71E-06 | 0.019307538 | 0.984595998 | -5.27E-06 | 5.37E-06 | 0.984595998 |
| MPA | f.26536.2.0 | Freesurfer ASEG | Volume-ratio of BrainSegVol-to-eTIV (whole brain) | 1.05E-05 | 0.070707589 | 1.13E-06 | 9.334205315 | 1.14E-20 | 8.33E-06 | 1.28E-05 | 2.28E-19 |
| MPA | f.26528.2.0 | Freesurfer ASEG | Volume of WM-hypointensities (whole brain) | -0.593788713 | -0.046575914 | 0.09492926 | -6.255065257 | 4.07E-10 | -0.77985993 | -0.407717496 | 4.07E-09 |
| MPA | f.25902.2.0 | Regional grey matter volumes (FAST) | Volume of grey matter in Crus I Cerebellum (right) | 0.239191463 | 0.030314835 | 0.057353956 | 4.17044402 | 3.06E-05 | 0.126771747 | 0.351611178 | 2.04E-04 |
| MPA | f.26666.2.0 | Freesurfer subsegmentation | Volume of PuI (left hemisphere) | -0.002509839 | -0.024899062 | 7.45E-04 | -3.369336108 | 7.55E-04 | -0.003969932 | -0.001049745 | 0.003775871 |
| MPA | f.25882.2.0 | Regional grey matter volumes (FAST) | Volume of grey matter in Putamen (left) | 0.048793648 | 0.025597474 | 0.014849171 | 3.285950986 | 0.001018451 | 0.019687729 | 0.077899567 | 0.004073805 |
| MPA | f.26564.2.0 | Freesurfer ASEG | Volume of Accumbens-area (left hemisphere) | -0.008000832 | -0.019824327 | 0.002815961 | -2.841244192 | 0.004499148 | -0.013520407 | -0.002481256 | 0.013636566 |
| MPA | f.25837.2.0 | Regional grey matter volumes (FAST) | Volume of grey matter in Paracingulate Gyrus (right) | -0.078119601 | -0.020117514 | 0.027960305 | -2.793946619 | 0.005212752 | -0.132924706 | -0.023314496 | 0.013636566 |
| MPA | f.25869.2.0 | Regional grey matter volumes (FAST) | Volume of grey matter in Planum Polare (right) | -0.020607028 | -0.020047233 | 0.007414627 | -2.779239951 | 0.005454627 | -0.035140467 | -0.006073588 | 0.013636566 |
| MPA | f.25846.2.0 | Regional grey matter volumes (FAST) | Volume of grey matter in Frontal Orbital Cortex (left) | -0.069999368 | -0.018313587 | 0.026576155 | -2.633916272 | 0.008448297 | -0.122091395 | -0.017907341 | 0.018388856 |
| MPA | f.26683.2.0 | Freesurfer subsegmentation | Volume of Pt (left hemisphere) | -7.13E-05 | -0.018969576 | 2.74E-05 | -2.605038067 | 0.009194428 | -1.25E-04 | -1.77E-05 | 0.018388856 |
| MPA | f.26573.2.0 | Freesurfer ASEG | Mean intensity of Thalamus-Proper (right hemisphere) | 1.50E-04 | 0.018323052 | 5.94E-05 | 2.520706351 | 0.011721055 | 3.33E-05 | 2.66E-04 | 0.021311009 |
| MPA | f.26548.2.0 | Freesurfer ASEG | Mean intensity of Accumbens-area (left hemisphere) | -1.60E-04 | -0.01608623 | 7.48E-05 | -2.134499139 | 0.032816248 | -3.06E-04 | -1.30E-05 | 0.054693747 |
| MPA | f.27051.2.0 | Freesurfer desikan gw | Grey-white contrast in superiorfrontal (right hemisphere) | 1.44E-04 | 0.012307979 | 8.16E-05 | 1.767898563 | 0.07709585 | -1.57E-05 | 3.04E-04 | 0.118609 |
| MPA | f.26688.2.0 | Freesurfer subsegmentation | Volume of LGN (right hemisphere) | 0.001531228 | 0.011781984 | 9.23E-04 | 1.658708058 | 0.097193137 | -2.78E-04 | 0.003340687 | 0.138847339 |
| MPA | f.25892.2.0 | Regional grey matter volumes (FAST) | Volume of grey matter in Brain-Stem | -0.036877137 | -0.01028026 | 0.025574102 | -1.441971891 | 0.149328779 | -0.087005036 | 0.013250762 | 0.199105039 |
| MPA | f.26586.2.0 | Freesurfer ASEG | Volume of Inf-Lat-Vent (right hemisphere) | -0.012432043 | -0.009664513 | 0.009159147 | -1.357336351 | 0.174692434 | -0.030384924 | 0.005520837 | 0.218365543 |
| MPA | f.26545.2.0 | Freesurfer ASEG | Mean intensity of Pallidum (left hemisphere) | -6.38E-05 | -0.008145188 | 6.07E-05 | -1.050431177 | 0.293534894 | -1.83E-04 | 5.52E-05 | 0.328144718 |
| MPA | f.27199.2.0 | Freesurfer DKT | Mean thickness of superiorfrontal (left hemisphere) | 5.48E-06 | 0.00761687 | 5.24E-06 | 1.046532337 | 0.295330246 | -4.79E-06 | 1.58E-05 | 0.328144718 |
| MPA | f.26717.2.0 | Freesurfer subsegmentation | Volume of Pons (whole brain) | -0.035369308 | -0.004169642 | 0.059638259 | -0.593064055 | 0.553146198 | -0.152266496 | 0.08152788 | 0.582259155 |
| MPA | f.27047.2.0 | Freesurfer desikan gw | Grey-white contrast in precentral (right hemisphere) | -9.44E-06 | -0.001082078 | 6.44E-05 | -0.146568036 | 0.883474728 | -1.36E-04 | 1.17E-04 | 0.883474728 |
| VPA | f.26536.2.0 | Freesurfer ASEG | Volume-ratio of BrainSegVol-to-eTIV (whole brain) | 4.41E-05 | 0.046179443 | 7.29E-06 | 6.056587385 | 1.42E-09 | 2.99E-05 | 5.84E-05 | 2.84E-08 |
| VPA | f.26528.2.0 | Freesurfer ASEG | Volume of WM-hypointensities (whole brain) | -2.273472142 | -0.027812617 | 0.612194598 | -3.71364293 | 2.05E-04 | -3.473437193 | -1.073507091 | 0.00204952 |
| VPA | f.25882.2.0 | Regional grey matter volumes (FAST) | Volume of grey matter in Putamen (left) | 0.315307918 | 0.025798323 | 0.09569005 | 3.295096167 | 9.86E-04 | 0.127745472 | 0.502870364 | 0.00657266 |
| VPA | f.25902.2.0 | Regional grey matter volumes (FAST) | Volume of grey matter in Crus I Cerebellum (right) | 1.071366412 | 0.021177288 | 0.369695241 | 2.89797188 | 0.003760655 | 0.34672531 | 1.796007515 | 0.018803274 |
| VPA | f.27199.2.0 | Freesurfer DKT | Mean thickness of superiorfrontal (left hemisphere) | 9.39E-05 | 0.020335782 | 3.38E-05 | 2.780570102 | 0.005432341 | 2.77E-05 | 1.60E-04 | 0.021729362 |
| VPA | f.25846.2.0 | Regional grey matter volumes (FAST) | Volume of grey matter in Frontal Orbital Cortex (left) | -0.391187317 | -0.015961983 | 0.171269303 | -2.28404805 | 0.022381015 | -0.726892954 | -0.05548168 | 0.074603384 |
| VPA | f.26548.2.0 | Freesurfer ASEG | Mean intensity of Accumbens-area (left hemisphere) | -0.001006986 | -0.01583305 | 4.82E-04 | -2.090334414 | 0.036602631 | -0.001951235 | -6.27E-05 | 0.08133918 |
| VPA | f.26545.2.0 | Freesurfer ASEG | Mean intensity of Pallidum (left hemisphere) | -8.33E-04 | -0.01659117 | 3.91E-04 | -2.129116057 | 0.033259033 | -0.00159981 | -6.61E-05 | 0.08133918 |
| VPA | f.26688.2.0 | Freesurfer subsegmentation | Volume of LGN (right hemisphere) | 0.012690489 | 0.015229294 | 0.005948563 | 2.133370348 | 0.032908676 | 0.001030686 | 0.024350291 | 0.08133918 |
| VPA | f.25892.2.0 | Regional grey matter volumes (FAST) | Volume of grey matter in Brain-Stem | -0.325135714 | -0.014136255 | 0.164794424 | -1.972977637 | 0.048514332 | -0.648149916 | -0.002121512 | 0.097028665 |
| VPA | f.25837.2.0 | Regional grey matter volumes (FAST) | Volume of grey matter in Paracingulate Gyrus (right) | -0.342431306 | -0.013753415 | 0.180202569 | -1.900257623 | 0.057416271 | -0.695647074 | 0.010784462 | 0.10439322 |
| VPA | f.26586.2.0 | Freesurfer ASEG | Volume of Inf-Lat-Vent (right hemisphere) | 0.057296833 | 0.006946897 | 0.059024444 | 0.970730583 | 0.33169631 | -0.058397213 | 0.17299088 | 0.442261746 |
| VPA | f.27051.2.0 | Freesurfer desikan gw | Grey-white contrast in superiorfrontal (right hemisphere) | 5.56E-04 | 0.00740444 | 5.26E-04 | 1.058154213 | 0.290000221 | -4.74E-04 | 0.001586655 | 0.442261746 |
| VPA | f.26683.2.0 | Freesurfer subsegmentation | Volume of Pt (left hemisphere) | -1.84E-04 | -0.007650753 | 1.76E-04 | -1.04520045 | 0.295945241 | -5.30E-04 | 1.61E-04 | 0.442261746 |
| VPA | f.26666.2.0 | Freesurfer subsegmentation | Volume of PuI (left hemisphere) | -0.004731041 | -0.007320095 | 0.004801752 | -0.985273903 | 0.324503612 | -0.014142975 | 0.004680892 | 0.442261746 |
| VPA | f.26573.2.0 | Freesurfer ASEG | Mean intensity of Thalamus-Proper (right hemisphere) | 3.07E-04 | 0.005860912 | 3.83E-04 | 0.802099729 | 0.422506517 | -4.43E-04 | 0.001057716 | 0.528133146 |
| VPA | f.26564.2.0 | Freesurfer ASEG | Volume of Accumbens-area (left hemisphere) | -0.012006079 | -0.004639672 | 0.018150547 | -0.661472025 | 0.508318641 | -0.047583037 | 0.02357088 | 0.56479849 |
| VPA | f.25869.2.0 | Regional grey matter volumes (FAST) | Volume of grey matter in Planum Polare (right) | -0.031929805 | -0.004844602 | 0.047791192 | -0.668110657 | 0.504072057 | -0.125605509 | 0.0617459 | 0.56479849 |
| VPA | f.27047.2.0 | Freesurfer desikan gw | Grey-white contrast in precentral (right hemisphere) | 1.29E-04 | 0.002310979 | 4.15E-04 | 0.311450875 | 0.755461714 | -6.84E-04 | 9.43E-04 | 0.795222856 |
| VPA | f.26717.2.0 | Freesurfer subsegmentation | Volume of Pons (whole brain) | -0.017664473 | -3.25E-04 | 0.384321587 | -0.04596274 | 0.963340489 | -0.770974735 | 0.735645789 | 0.963340489 |
| MVPA | f.26536.2.0 | Freesurfer ASEG | Volume-ratio of BrainSegVol-to-eTIV (whole brain) | 9.96E-06 | 0.072397057 | 1.05E-06 | 9.519727296 | 1.96E-21 | 7.91E-06 | 1.20E-05 | 3.93E-20 |
| MVPA | f.26528.2.0 | Freesurfer ASEG | Volume of WM-hypointensities (whole brain) | -0.556533365 | -0.047314499 | 0.087936047 | -6.328842184 | 2.53E-10 | -0.728897159 | -0.384169571 | 2.53E-09 |
| MVPA | f.25902.2.0 | Regional grey matter volumes (FAST) | Volume of grey matter in Crus I Cerebellum (right) | 0.227398766 | 0.031237137 | 0.053128827 | 4.280139029 | 1.88E-05 | 0.123260742 | 0.33153679 | 1.25E-04 |
| MVPA | f.25882.2.0 | Regional grey matter volumes (FAST) | Volume of grey matter in Putamen (left) | 0.04838742 | 0.027513122 | 0.013755004 | 3.517804807 | 4.36E-04 | 0.021426182 | 0.075348658 | 0.002181356 |
| MVPA | f.26666.2.0 | Freesurfer subsegmentation | Volume of PuI (left hemisphere) | -0.002251554 | -0.024209913 | 6.90E-04 | -3.262820766 | 0.001105275 | -0.003604151 | -8.99E-04 | 0.004421101 |
| MVPA | f.25837.2.0 | Regional grey matter volumes (FAST) | Volume of grey matter in Paracingulate Gyrus (right) | -0.074113645 | -0.020686454 | 0.025900953 | -2.861425441 | 0.004222567 | -0.124882204 | -0.023345085 | 0.014075224 |
| MVPA | f.26564.2.0 | Freesurfer ASEG | Volume of Accumbens-area (left hemisphere) | -0.007113923 | -0.019104957 | 0.002608636 | -2.72706652 | 0.006396589 | -0.01222712 | -0.002000726 | 0.015991472 |
| MVPA | f.25846.2.0 | Regional grey matter volumes (FAST) | Volume of grey matter in Frontal Orbital Cortex (left) | -0.068152882 | -0.019325784 | 0.024618499 | -2.768360627 | 0.005640025 | -0.116407699 | -0.019898066 | 0.015991472 |
| MVPA | f.25869.2.0 | Regional grey matter volumes (FAST) | Volume of grey matter in Planum Polare (right) | -0.018343502 | -0.019341712 | 0.006868717 | -2.670586426 | 0.007579114 | -0.0318069 | -0.004880103 | 0.016842475 |
| MVPA | f.26683.2.0 | Freesurfer subsegmentation | Volume of Pt (left hemisphere) | -6.50E-05 | -0.018742365 | 2.54E-05 | -2.563455515 | 0.010372146 | -1.15E-04 | -1.53E-05 | 0.020744291 |
| MVPA | f.26573.2.0 | Freesurfer ASEG | Mean intensity of Thalamus-Proper (right hemisphere) | 1.35E-04 | 0.017883997 | 5.50E-05 | 2.45037318 | 0.014280858 | 2.70E-05 | 2.43E-04 | 0.025965196 |
| MVPA | f.26548.2.0 | Freesurfer ASEG | Mean intensity of Accumbens-area (left hemisphere) | -1.58E-04 | -0.017235597 | 6.92E-05 | -2.277843229 | 0.022748313 | -2.93E-04 | -2.20E-05 | 0.037913854 |
| MVPA | f.26688.2.0 | Freesurfer subsegmentation | Volume of LGN (right hemisphere) | 0.001576249 | 0.013145501 | 8.55E-04 | 1.843251708 | 0.065309716 | -9.99E-05 | 0.003252424 | 0.100476486 |
| MVPA | f.27051.2.0 | Freesurfer desikan gw | Grey-white contrast in superiorfrontal (right hemisphere) | 1.35E-04 | 0.012511022 | 7.56E-05 | 1.789831155 | 0.073498904 | -1.29E-05 | 2.83E-04 | 0.104998434 |
| MVPA | f.25892.2.0 | Regional grey matter volumes (FAST) | Volume of grey matter in Brain-Stem | -0.038364489 | -0.011591774 | 0.023690387 | -1.619411671 | 0.105377321 | -0.08480011 | 0.008071132 | 0.140503095 |
| MVPA | f.27199.2.0 | Freesurfer DKT | Mean thickness of superiorfrontal (left hemisphere) | 6.65E-06 | 0.010004847 | 4.85E-06 | 1.369128562 | 0.170977233 | -2.87E-06 | 1.62E-05 | 0.213721541 |
| MVPA | f.26545.2.0 | Freesurfer ASEG | Mean intensity of Pallidum (left hemisphere) | -7.19E-05 | -0.009958482 | 5.62E-05 | -1.279128466 | 0.200869331 | -1.82E-04 | 3.83E-05 | 0.236316861 |
| MVPA | f.26586.2.0 | Freesurfer ASEG | Volume of Inf-Lat-Vent (right hemisphere) | -0.009484401 | -0.007991364 | 0.008484795 | -1.117811425 | 0.263663384 | -0.02611548 | 0.007146679 | 0.292959315 |
| MVPA | f.26717.2.0 | Freesurfer subsegmentation | Volume of Pons (whole brain) | -0.030716797 | -0.003924838 | 0.05524643 | -0.555996048 | 0.578220896 | -0.139005542 | 0.077571949 | 0.608653574 |
| MVPA | f.27047.2.0 | Freesurfer desikan gw | Grey-white contrast in precentral (right hemisphere) | -5.43E-06 | -6.75E-04 | 5.97E-05 | -0.09100286 | 0.927491394 | -1.22E-04 | 1.12E-04 | 0.927491394 |

Note: β_std_ means standardized β. *P*-FDR was calculated by FDR correction (α=0.05) of *P*. Models were adjusted for age, sex, ethnicity, educational attainment, Townsend deprivation index, smoking status, alcohol consumption, and dietary pattern.

**Table S15. Nonlinear associations between PA and brain structure**

| **Exposure** | **Outcome** | **Features** | **Category** | **EDF** | **F-statistic** | ***P*** | ***P*-FDR** | **nlogP-FDR** |
| --- | --- | --- | --- | --- | --- | --- | --- | --- |
| LPA | f.26789.2.0 | Volume of bankssts (left hemisphere) | Freesurfer desikan white | 1.000000036 | 0.03620374 | 0.849098191 | 0.889531438 | 0.117060429 |
| LPA | f.26790.2.0 | Volume of caudalanteriorcingulate (left hemisphere) | Freesurfer desikan white | 1.000000023 | 0.074115267 | 0.785440466 | 0.849820832 | 0.162729737 |
| LPA | f.26791.2.0 | Volume of caudalmiddlefrontal (left hemisphere) | Freesurfer desikan white | 1.000000024 | 4.000074274 | 0.045514059 | 0.215989689 | 1.53252461 |
| LPA | f.26792.2.0 | Volume of cuneus (left hemisphere) | Freesurfer desikan white | 1.345596336 | 2.859884204 | 0.04583935 | 0.215989689 | 1.53252461 |
| LPA | f.26793.2.0 | Volume of entorhinal (left hemisphere) | Freesurfer desikan white | 1.752058126 | 0.902737988 | 0.44798098 | 0.64275532 | 0.441991157 |
| LPA | f.26794.2.0 | Volume of fusiform (left hemisphere) | Freesurfer desikan white | 1.047345594 | 1.569633286 | 0.192045877 | 0.422000059 | 0.862749826 |
| LPA | f.26795.2.0 | Volume of inferiorparietal (left hemisphere) | Freesurfer desikan white | 1.542844904 | 0.705722061 | 0.421920074 | 0.618816109 | 0.479947128 |
| LPA | f.26796.2.0 | Volume of inferiortemporal (left hemisphere) | Freesurfer desikan white | 1.000000021 | 0.02901073 | 0.864756745 | 0.891780393 | 0.114535373 |
| LPA | f.26797.2.0 | Volume of isthmuscingulate (left hemisphere) | Freesurfer desikan white | 1.194550984 | 3.502356547 | 0.070891413 | 0.222801583 | 1.501473668 |
| LPA | f.26798.2.0 | Volume of lateraloccipital (left hemisphere) | Freesurfer desikan white | 2.647744916 | 0.970686175 | 0.350251891 | 0.571557481 | 0.559390222 |
| LPA | f.26799.2.0 | Volume of lateralorbitofrontal (left hemisphere) | Freesurfer desikan white | 1.000000032 | 0.227422084 | 0.633448522 | 0.733466709 | 0.309973069 |
| LPA | f.26800.2.0 | Volume of lingual (left hemisphere) | Freesurfer desikan white | 5.278760056 | 2.579448526 | 0.015499864 | 0.215989689 | 1.53252461 |
| LPA | f.26801.2.0 | Volume of medialorbitofrontal (left hemisphere) | Freesurfer desikan white | 1.314423181 | 3.795563764 | 0.021551635 | 0.215989689 | 1.53252461 |
| LPA | f.26802.2.0 | Volume of middletemporal (left hemisphere) | Freesurfer desikan white | 2.482546455 | 0.975142664 | 0.398807633 | 0.607951905 | 0.497659504 |
| LPA | f.26803.2.0 | Volume of parahippocampal (left hemisphere) | Freesurfer desikan white | 1.000000137 | 11.5941381 | 6.63E-04 | 0.043757737 | 3.12908684 |
| LPA | f.26804.2.0 | Volume of paracentral (left hemisphere) | Freesurfer desikan white | 1.851759309 | 2.785451491 | 0.058971756 | 0.222801583 | 1.501473668 |
| LPA | f.26805.2.0 | Volume of parsopercularis (left hemisphere) | Freesurfer desikan white | 1.209097935 | 4.12002758 | 0.046179832 | 0.215989689 | 1.53252461 |
| LPA | f.26806.2.0 | Volume of parsorbitalis (left hemisphere) | Freesurfer desikan white | 1.000000021 | 0.134079102 | 0.714244661 | 0.798985553 | 0.224412415 |
| LPA | f.26807.2.0 | Volume of parstriangularis (left hemisphere) | Freesurfer desikan white | 1.407685024 | 1.540853761 | 0.315613512 | 0.562986265 | 0.574500047 |
| LPA | f.26808.2.0 | Volume of pericalcarine (left hemisphere) | Freesurfer desikan white | 3.708466853 | 2.201219023 | 0.06874818 | 0.222801583 | 1.501473668 |
| LPA | f.26809.2.0 | Volume of postcentral (left hemisphere) | Freesurfer desikan white | 1.000000069 | 2.831476678 | 0.092451612 | 0.265295932 | 1.326909353 |
| LPA | f.26810.2.0 | Volume of posteriorcingulate (left hemisphere) | Freesurfer desikan white | 1.000000011 | 0.454703389 | 0.500119106 | 0.647212961 | 0.435079888 |
| LPA | f.26811.2.0 | Volume of precentral (left hemisphere) | Freesurfer desikan white | 2.073870965 | 3.247046996 | 0.029988555 | 0.215989689 | 1.53252461 |
| LPA | f.26812.2.0 | Volume of precuneus (left hemisphere) | Freesurfer desikan white | 1.869729223 | 1.638482263 | 0.182678306 | 0.422000059 | 0.862749826 |
| LPA | f.26813.2.0 | Volume of rostralanteriorcingulate (left hemisphere) | Freesurfer desikan white | 4.455821171 | 1.472042397 | 0.198496376 | 0.422000059 | 0.862749826 |
| LPA | f.26814.2.0 | Volume of rostralmiddlefrontal (left hemisphere) | Freesurfer desikan white | 1.000000018 | 0.457934597 | 0.49860017 | 0.647212961 | 0.435079888 |
| LPA | f.26815.2.0 | Volume of superiorfrontal (left hemisphere) | Freesurfer desikan white | 1.329850634 | 2.817081141 | 0.047957826 | 0.215989689 | 1.53252461 |
| LPA | f.26816.2.0 | Volume of superiorparietal (left hemisphere) | Freesurfer desikan white | 2.248759887 | 2.278307587 | 0.109203941 | 0.288298405 | 1.243759207 |
| LPA | f.26817.2.0 | Volume of superiortemporal (left hemisphere) | Freesurfer desikan white | 1.000000021 | 0.053430758 | 0.817200917 | 0.869923557 | 0.139349937 |
| LPA | f.26818.2.0 | Volume of supramarginal (left hemisphere) | Freesurfer desikan white | 1.365163871 | 2.39744105 | 0.066657232 | 0.222801583 | 1.501473668 |
| LPA | f.26819.2.0 | Volume of frontalpole (left hemisphere) | Freesurfer desikan white | 1.000000021 | 0.087499125 | 0.76738509 | 0.844123599 | 0.169456351 |
| LPA | f.26820.2.0 | Volume of transversetemporal (left hemisphere) | Freesurfer desikan white | 1.000000023 | 0.490749252 | 0.483602974 | 0.647212961 | 0.435079888 |
| LPA | f.26821.2.0 | Volume of insula (left hemisphere) | Freesurfer desikan white | 1.000000035 | 2.692661252 | 0.100829738 | 0.277281781 | 1.282721032 |
| LPA | f.26890.2.0 | Volume of bankssts (right hemisphere) | Freesurfer desikan white | 1.000000024 | 0.868370747 | 0.351419995 | 0.571557481 | 0.559390222 |
| LPA | f.26891.2.0 | Volume of caudalanteriorcingulate (right hemisphere) | Freesurfer desikan white | 1.000000024 | 1.038085773 | 0.308281574 | 0.562986265 | 0.574500047 |
| LPA | f.26892.2.0 | Volume of caudalmiddlefrontal (right hemisphere) | Freesurfer desikan white | 1.000000022 | 0.005887467 | 0.938840092 | 0.943052566 | 0.058633255 |
| LPA | f.26893.2.0 | Volume of cuneus (right hemisphere) | Freesurfer desikan white | 1.000000055 | 0.490200245 | 0.48384767 | 0.647212961 | 0.435079888 |
| LPA | f.26894.2.0 | Volume of entorhinal (right hemisphere) | Freesurfer desikan white | 1.539321816 | 1.09786229 | 0.267327244 | 0.518929356 | 0.655987521 |
| LPA | f.26895.2.0 | Volume of fusiform (right hemisphere) | Freesurfer desikan white | 1.000000014 | 3.839221576 | 0.050083007 | 0.215989689 | 1.53252461 |
| LPA | f.26896.2.0 | Volume of inferiorparietal (right hemisphere) | Freesurfer desikan white | 1.671797356 | 1.59904224 | 0.204606089 | 0.422000059 | 0.862749826 |
| LPA | f.26897.2.0 | Volume of inferiortemporal (right hemisphere) | Freesurfer desikan white | 1.000000025 | 0.69257598 | 0.40530127 | 0.607951905 | 0.497659504 |
| LPA | f.26898.2.0 | Volume of isthmuscingulate (right hemisphere) | Freesurfer desikan white | 1.000000022 | 3.424304359 | 0.064260577 | 0.222801583 | 1.501473668 |
| LPA | f.26899.2.0 | Volume of lateraloccipital (right hemisphere) | Freesurfer desikan white | 2.580305199 | 1.000601208 | 0.341949903 | 0.571557481 | 0.559390222 |
| LPA | f.26900.2.0 | Volume of lateralorbitofrontal (right hemisphere) | Freesurfer desikan white | 1.000000021 | 0.005103042 | 0.943052566 | 0.943052566 | 0.058633255 |
| LPA | f.26901.2.0 | Volume of lingual (right hemisphere) | Freesurfer desikan white | 3.222019764 | 1.487282202 | 0.197006679 | 0.422000059 | 0.862749826 |
| LPA | f.26902.2.0 | Volume of medialorbitofrontal (right hemisphere) | Freesurfer desikan white | 1.000000042 | 0.376414972 | 0.5395366 | 0.668677403 | 0.402453543 |
| LPA | f.26903.2.0 | Volume of middletemporal (right hemisphere) | Freesurfer desikan white | 1.789219983 | 1.52739263 | 0.253072208 | 0.506144416 | 0.680933243 |
| LPA | f.26904.2.0 | Volume of parahippocampal (right hemisphere) | Freesurfer desikan white | 2.693929413 | 3.059686691 | 0.02657539 | 0.215989689 | 1.53252461 |
| LPA | f.26905.2.0 | Volume of paracentral (right hemisphere) | Freesurfer desikan white | 1.547623671 | 3.59194067 | 0.021266822 | 0.215989689 | 1.53252461 |
| LPA | f.26906.2.0 | Volume of parsopercularis (right hemisphere) | Freesurfer desikan white | 1.000000071 | 0.382743678 | 0.536147061 | 0.668677403 | 0.402453543 |
| LPA | f.26907.2.0 | Volume of parsorbitalis (right hemisphere) | Freesurfer desikan white | 1.000000021 | 3.764717538 | 0.052361137 | 0.215989689 | 1.53252461 |
| LPA | f.26908.2.0 | Volume of parstriangularis (right hemisphere) | Freesurfer desikan white | 1.722008043 | 0.857541269 | 0.48261981 | 0.647212961 | 0.435079888 |
| LPA | f.26909.2.0 | Volume of pericalcarine (right hemisphere) | Freesurfer desikan white | 2.866926497 | 1.064219332 | 0.293340961 | 0.553157241 | 0.592112977 |
| LPA | f.26910.2.0 | Volume of postcentral (right hemisphere) | Freesurfer desikan white | 1.000000047 | 2.47223497 | 0.115891593 | 0.294186352 | 1.223541862 |
| LPA | f.26911.2.0 | Volume of posteriorcingulate (right hemisphere) | Freesurfer desikan white | 1.17688309 | 1.409413643 | 0.188920277 | 0.422000059 | 0.862749826 |
| LPA | f.26912.2.0 | Volume of precentral (right hemisphere) | Freesurfer desikan white | 2.609055736 | 3.142456713 | 0.020150852 | 0.215989689 | 1.53252461 |
| LPA | f.26913.2.0 | Volume of precuneus (right hemisphere) | Freesurfer desikan white | 1.729523309 | 1.140643072 | 0.355058435 | 0.571557481 | 0.559390222 |
| LPA | f.26914.2.0 | Volume of rostralanteriorcingulate (right hemisphere) | Freesurfer desikan white | 1.000000109 | 2.853823189 | 0.091175204 | 0.265295932 | 1.326909353 |
| LPA | f.26915.2.0 | Volume of rostralmiddlefrontal (right hemisphere) | Freesurfer desikan white | 1.683258673 | 0.537350485 | 0.547099693 | 0.668677403 | 0.402453543 |
| LPA | f.26916.2.0 | Volume of superiorfrontal (right hemisphere) | Freesurfer desikan white | 1.000000018 | 4.676365465 | 0.030594201 | 0.215989689 | 1.53252461 |
| LPA | f.26917.2.0 | Volume of superiorparietal (right hemisphere) | Freesurfer desikan white | 2.273561064 | 1.225945633 | 0.381646815 | 0.59973071 | 0.511274541 |
| LPA | f.26918.2.0 | Volume of superiortemporal (right hemisphere) | Freesurfer desikan white | 1.000000012 | 0.259995153 | 0.610130459 | 0.719082327 | 0.329779426 |
| LPA | f.26919.2.0 | Volume of supramarginal (right hemisphere) | Freesurfer desikan white | 1.000000022 | 0.314023614 | 0.575229308 | 0.69027517 | 0.370664964 |
| LPA | f.26920.2.0 | Volume of frontalpole (right hemisphere) | Freesurfer desikan white | 1.455818428 | 0.284539664 | 0.698466181 | 0.794806344 | 0.229656786 |
| LPA | f.26921.2.0 | Volume of transversetemporal (right hemisphere) | Freesurfer desikan white | 1.000000016 | 4.928112734 | 0.026436214 | 0.215989689 | 1.53252461 |
| LPA | f.26922.2.0 | Volume of insula (right hemisphere) | Freesurfer desikan white | 1.000000024 | 4.272283077 | 0.038753865 | 0.215989689 | 1.53252461 |
| MPA | f.26789.2.0 | Volume of bankssts (left hemisphere) | Freesurfer desikan white | 4.906363344 | 1.540295421 | 0.145246586 | 0.191725494 | 1.651690651 |
| MPA | f.26790.2.0 | Volume of caudalanteriorcingulate (left hemisphere) | Freesurfer desikan white | 2.263536669 | 1.270811618 | 0.21867304 | 0.264557322 | 1.329697332 |
| MPA | f.26791.2.0 | Volume of caudalmiddlefrontal (left hemisphere) | Freesurfer desikan white | 1.633113568 | 2.563272312 | 0.072269976 | 0.125462179 | 2.075750932 |
| MPA | f.26792.2.0 | Volume of cuneus (left hemisphere) | Freesurfer desikan white | 1.219629802 | 0.049506588 | 0.884988912 | 0.884988912 | 0.122180163 |
| MPA | f.26793.2.0 | Volume of entorhinal (left hemisphere) | Freesurfer desikan white | 6.099828742 | 1.567818939 | 0.152840495 | 0.197793582 | 1.620531306 |
| MPA | f.26794.2.0 | Volume of fusiform (left hemisphere) | Freesurfer desikan white | 5.279278466 | 1.862319595 | 0.08576186 | 0.134768638 | 2.004195766 |
| MPA | f.26795.2.0 | Volume of inferiorparietal (left hemisphere) | Freesurfer desikan white | 4.783995818 | 1.179904335 | 0.311819048 | 0.354828572 | 1.036120501 |
| MPA | f.26796.2.0 | Volume of inferiortemporal (left hemisphere) | Freesurfer desikan white | 5.758510714 | 1.345469145 | 0.220464435 | 0.264557322 | 1.329697332 |
| MPA | f.26797.2.0 | Volume of isthmuscingulate (left hemisphere) | Freesurfer desikan white | 1.000000026 | 16.84445155 | 4.14E-05 | 0.001365413 | 6.596298695 |
| MPA | f.26798.2.0 | Volume of lateraloccipital (left hemisphere) | Freesurfer desikan white | 5.28111547 | 2.48588844 | 0.018953967 | 0.053647174 | 2.925326495 |
| MPA | f.26799.2.0 | Volume of lateralorbitofrontal (left hemisphere) | Freesurfer desikan white | 5.430153575 | 2.851795514 | 0.007466084 | 0.028690869 | 3.551176356 |
| MPA | f.26800.2.0 | Volume of lingual (left hemisphere) | Freesurfer desikan white | 2.69479617 | 2.152479698 | 0.076037684 | 0.125462179 | 2.075750932 |
| MPA | f.26801.2.0 | Volume of medialorbitofrontal (left hemisphere) | Freesurfer desikan white | 5.567546231 | 2.351991707 | 0.028282366 | 0.06566985 | 2.723115369 |
| MPA | f.26802.2.0 | Volume of middletemporal (left hemisphere) | Freesurfer desikan white | 2.089667701 | 1.255429929 | 0.350975106 | 0.392616221 | 0.934922682 |
| MPA | f.26803.2.0 | Volume of parahippocampal (left hemisphere) | Freesurfer desikan white | 1.000000077 | 1.844903365 | 0.174395265 | 0.217171462 | 1.527068089 |
| MPA | f.26804.2.0 | Volume of paracentral (left hemisphere) | Freesurfer desikan white | 2.452855137 | 1.736015083 | 0.160710612 | 0.203978854 | 1.589738946 |
| MPA | f.26805.2.0 | Volume of parsopercularis (left hemisphere) | Freesurfer desikan white | 5.196552701 | 2.878169748 | 0.007824782 | 0.028690869 | 3.551176356 |
| MPA | f.26806.2.0 | Volume of parsorbitalis (left hemisphere) | Freesurfer desikan white | 6.310625585 | 2.067096989 | 0.040772485 | 0.08409325 | 2.47582898 |
| MPA | f.26807.2.0 | Volume of parstriangularis (left hemisphere) | Freesurfer desikan white | 4.373697898 | 2.817400469 | 0.012880229 | 0.040480721 | 3.206929442 |
| MPA | f.26808.2.0 | Volume of pericalcarine (left hemisphere) | Freesurfer desikan white | 1.588048468 | 0.522976608 | 0.575178556 | 0.584027457 | 0.537807281 |
| MPA | f.26809.2.0 | Volume of postcentral (left hemisphere) | Freesurfer desikan white | 6.922826348 | 2.484751679 | 0.010624475 | 0.035060768 | 3.350672494 |
| MPA | f.26810.2.0 | Volume of posteriorcingulate (left hemisphere) | Freesurfer desikan white | 2.236553155 | 1.159696328 | 0.270983694 | 0.313770593 | 1.159093155 |
| MPA | f.26811.2.0 | Volume of precentral (left hemisphere) | Freesurfer desikan white | 5.668053942 | 4.348634258 | 1.22E-04 | 0.002007604 | 6.210813383 |
| MPA | f.26812.2.0 | Volume of precuneus (left hemisphere) | Freesurfer desikan white | 5.523096838 | 4.635068418 | 6.34E-05 | 0.001394381 | 6.575304745 |
| MPA | f.26813.2.0 | Volume of rostralanteriorcingulate (left hemisphere) | Freesurfer desikan white | 1.259245873 | 9.034225603 | 0.002164454 | 0.012986721 | 4.343827882 |
| MPA | f.26814.2.0 | Volume of rostralmiddlefrontal (left hemisphere) | Freesurfer desikan white | 8.011218382 | 3.098706758 | 8.28E-04 | 0.006827237 | 4.986835292 |
| MPA | f.26815.2.0 | Volume of superiorfrontal (left hemisphere) | Freesurfer desikan white | 6.635099274 | 2.964857913 | 0.003736064 | 0.017612871 | 4.039125342 |
| MPA | f.26816.2.0 | Volume of superiorparietal (left hemisphere) | Freesurfer desikan white | 5.384758424 | 3.661550199 | 9.97E-04 | 0.007310956 | 4.9183812 |
| MPA | f.26817.2.0 | Volume of superiortemporal (left hemisphere) | Freesurfer desikan white | 2.070605015 | 1.894429503 | 0.144075207 | 0.191725494 | 1.651690651 |
| MPA | f.26818.2.0 | Volume of supramarginal (left hemisphere) | Freesurfer desikan white | 6.640294743 | 3.990539366 | 2.26E-04 | 0.002980518 | 5.815658277 |
| MPA | f.26819.2.0 | Volume of frontalpole (left hemisphere) | Freesurfer desikan white | 6.506704615 | 1.656458598 | 0.117759843 | 0.161919785 | 1.820654223 |
| MPA | f.26820.2.0 | Volume of transversetemporal (left hemisphere) | Freesurfer desikan white | 5.477241557 | 2.71795005 | 0.010427115 | 0.035060768 | 3.350672494 |
| MPA | f.26821.2.0 | Volume of insula (left hemisphere) | Freesurfer desikan white | 5.367090394 | 2.506210595 | 0.018623396 | 0.053647174 | 2.925326495 |
| MPA | f.26890.2.0 | Volume of bankssts (right hemisphere) | Freesurfer desikan white | 3.167833797 | 0.725357164 | 0.553414383 | 0.570708583 | 0.560876563 |
| MPA | f.26891.2.0 | Volume of caudalanteriorcingulate (right hemisphere) | Freesurfer desikan white | 1.000000023 | 3.514578925 | 0.06084818 | 0.108539998 | 2.220636533 |
| MPA | f.26892.2.0 | Volume of caudalmiddlefrontal (right hemisphere) | Freesurfer desikan white | 1.912875376 | 3.421630315 | 0.024370194 | 0.059571586 | 2.820576562 |
| MPA | f.26893.2.0 | Volume of cuneus (right hemisphere) | Freesurfer desikan white | 4.389872417 | 1.025288607 | 0.460295609 | 0.482214448 | 0.729366351 |
| MPA | f.26894.2.0 | Volume of entorhinal (right hemisphere) | Freesurfer desikan white | 5.853579114 | 1.966125839 | 0.057081969 | 0.104650277 | 2.257131184 |
| MPA | f.26895.2.0 | Volume of fusiform (right hemisphere) | Freesurfer desikan white | 5.688006578 | 2.09956677 | 0.04704629 | 0.091325152 | 2.393329047 |
| MPA | f.26896.2.0 | Volume of inferiorparietal (right hemisphere) | Freesurfer desikan white | 4.940253754 | 2.106542943 | 0.049174322 | 0.092728721 | 2.378077029 |
| MPA | f.26897.2.0 | Volume of inferiortemporal (right hemisphere) | Freesurfer desikan white | 4.947843764 | 1.026533831 | 0.409958506 | 0.436407442 | 0.829178972 |
| MPA | f.26898.2.0 | Volume of isthmuscingulate (right hemisphere) | Freesurfer desikan white | 4.305091493 | 3.478632326 | 0.003059131 | 0.015530972 | 4.164919061 |
| MPA | f.26899.2.0 | Volume of lateraloccipital (right hemisphere) | Freesurfer desikan white | 6.692341447 | 2.230186933 | 0.020710794 | 0.054676496 | 2.90632136 |
| MPA | f.26900.2.0 | Volume of lateralorbitofrontal (right hemisphere) | Freesurfer desikan white | 5.529872393 | 2.087495952 | 0.04481858 | 0.089637159 | 2.411985322 |
| MPA | f.26901.2.0 | Volume of lingual (right hemisphere) | Freesurfer desikan white | 2.137787823 | 1.401353615 | 0.268483814 | 0.313770593 | 1.159093155 |
| MPA | f.26902.2.0 | Volume of medialorbitofrontal (right hemisphere) | Freesurfer desikan white | 6.115532822 | 1.68718868 | 0.117503765 | 0.161919785 | 1.820654223 |
| MPA | f.26903.2.0 | Volume of middletemporal (right hemisphere) | Freesurfer desikan white | 5.341678163 | 1.772667401 | 0.096005623 | 0.144008434 | 1.937883409 |
| MPA | f.26904.2.0 | Volume of parahippocampal (right hemisphere) | Freesurfer desikan white | 2.71468578 | 2.180205938 | 0.075837703 | 0.125462179 | 2.075750932 |
| MPA | f.26905.2.0 | Volume of paracentral (right hemisphere) | Freesurfer desikan white | 2.511491701 | 3.16102266 | 0.022564999 | 0.057280382 | 2.859797092 |
| MPA | f.26906.2.0 | Volume of parsopercularis (right hemisphere) | Freesurfer desikan white | 5.097116909 | 3.041689311 | 0.005339999 | 0.023495995 | 3.750925304 |
| MPA | f.26907.2.0 | Volume of parsorbitalis (right hemisphere) | Freesurfer desikan white | 7.094201726 | 2.246488719 | 0.019508063 | 0.053647174 | 2.925326495 |
| MPA | f.26908.2.0 | Volume of parstriangularis (right hemisphere) | Freesurfer desikan white | 1.000000025 | 4.63860633 | 0.031274311 | 0.068803484 | 2.676500894 |
| MPA | f.26909.2.0 | Volume of pericalcarine (right hemisphere) | Freesurfer desikan white | 4.277667038 | 1.045701908 | 0.403276459 | 0.436331906 | 0.829352073 |
| MPA | f.26910.2.0 | Volume of postcentral (right hemisphere) | Freesurfer desikan white | 5.653649345 | 2.210515884 | 0.028854934 | 0.06566985 | 2.723115369 |
| MPA | f.26911.2.0 | Volume of posteriorcingulate (right hemisphere) | Freesurfer desikan white | 3.712569141 | 1.823351677 | 0.089874586 | 0.13794704 | 1.980885438 |
| MPA | f.26912.2.0 | Volume of precentral (right hemisphere) | Freesurfer desikan white | 5.563761384 | 3.75596606 | 7.06E-04 | 0.00665628 | 5.012194506 |
| MPA | f.26913.2.0 | Volume of precuneus (right hemisphere) | Freesurfer desikan white | 5.05998349 | 3.364877385 | 0.002530054 | 0.013915299 | 4.274766399 |
| MPA | f.26914.2.0 | Volume of rostralanteriorcingulate (right hemisphere) | Freesurfer desikan white | 1.00000001 | 7.393252565 | 0.006553439 | 0.027032937 | 3.610699251 |
| MPA | f.26915.2.0 | Volume of rostralmiddlefrontal (right hemisphere) | Freesurfer desikan white | 1.00000001 | 3.029404365 | 0.081786054 | 0.131655599 | 2.027565868 |
| MPA | f.26916.2.0 | Volume of superiorfrontal (right hemisphere) | Freesurfer desikan white | 5.643901421 | 2.183596886 | 0.035231084 | 0.075008113 | 2.590158994 |
| MPA | f.26917.2.0 | Volume of superiorparietal (right hemisphere) | Freesurfer desikan white | 5.847260103 | 3.707802329 | 6.03E-04 | 0.006628486 | 5.016378854 |
| MPA | f.26918.2.0 | Volume of superiortemporal (right hemisphere) | Freesurfer desikan white | 1.00000003 | 0.838829862 | 0.359744612 | 0.395719074 | 0.927050729 |
| MPA | f.26919.2.0 | Volume of supramarginal (right hemisphere) | Freesurfer desikan white | 5.005985089 | 3.519999208 | 0.001749661 | 0.011547763 | 4.461263566 |
| MPA | f.26920.2.0 | Volume of frontalpole (right hemisphere) | Freesurfer desikan white | 1.00000001 | 2.574146143 | 0.108641208 | 0.155876515 | 1.858691153 |
| MPA | f.26921.2.0 | Volume of transversetemporal (right hemisphere) | Freesurfer desikan white | 1.000000794 | 23.74597624 | 1.28E-06 | 8.47E-05 | 9.376728694 |
| MPA | f.26922.2.0 | Volume of insula (right hemisphere) | Freesurfer desikan white | 5.413471488 | 1.741337933 | 0.101450756 | 0.148794441 | 1.905189513 |
| MVPA | f.26789.2.0 | Volume of bankssts (left hemisphere) | Freesurfer desikan white | 5.140063768 | 1.599775294 | 0.151354688 | 0.203865498 | 1.590294827 |
| MVPA | f.26790.2.0 | Volume of caudalanteriorcingulate (left hemisphere) | Freesurfer desikan white | 2.06708938 | 1.161964399 | 0.244464522 | 0.298789972 | 1.208014388 |
| MVPA | f.26791.2.0 | Volume of caudalmiddlefrontal (left hemisphere) | Freesurfer desikan white | 1.820353796 | 2.334028505 | 0.076540598 | 0.132938934 | 2.0178654 |
| MVPA | f.26792.2.0 | Volume of cuneus (left hemisphere) | Freesurfer desikan white | 1.250966083 | 0.068978079 | 0.868841494 | 0.868841494 | 0.140594571 |
| MVPA | f.26793.2.0 | Volume of entorhinal (left hemisphere) | Freesurfer desikan white | 1.387192533 | 0.447191253 | 0.497400135 | 0.529490467 | 0.635840119 |
| MVPA | f.26794.2.0 | Volume of fusiform (left hemisphere) | Freesurfer desikan white | 5.171805093 | 1.696652324 | 0.131708611 | 0.191867572 | 1.650949875 |
| MVPA | f.26795.2.0 | Volume of inferiorparietal (left hemisphere) | Freesurfer desikan white | 1.88339802 | 0.823468023 | 0.525756967 | 0.550793013 | 0.596396198 |
| MVPA | f.26796.2.0 | Volume of inferiortemporal (left hemisphere) | Freesurfer desikan white | 1.923618375 | 0.931441681 | 0.397968171 | 0.452860332 | 0.792171519 |
| MVPA | f.26797.2.0 | Volume of isthmuscingulate (left hemisphere) | Freesurfer desikan white | 1.000000031 | 16.91243254 | 3.99E-05 | 0.001315982 | 6.633172461 |
| MVPA | f.26798.2.0 | Volume of lateraloccipital (left hemisphere) | Freesurfer desikan white | 4.935087623 | 2.353954788 | 0.028170268 | 0.071509141 | 2.637929993 |
| MVPA | f.26799.2.0 | Volume of lateralorbitofrontal (left hemisphere) | Freesurfer desikan white | 4.993501702 | 3.010238238 | 0.006155385 | 0.025390964 | 3.673361927 |
| MVPA | f.26800.2.0 | Volume of lingual (left hemisphere) | Freesurfer desikan white | 2.547027723 | 1.822769457 | 0.133703045 | 0.191867572 | 1.650949875 |
| MVPA | f.26801.2.0 | Volume of medialorbitofrontal (left hemisphere) | Freesurfer desikan white | 5.655605257 | 2.632533659 | 0.014012789 | 0.04402803 | 3.122928808 |
| MVPA | f.26802.2.0 | Volume of middletemporal (left hemisphere) | Freesurfer desikan white | 1.949356569 | 1.219543145 | 0.341757607 | 0.395719335 | 0.92705007 |
| MVPA | f.26803.2.0 | Volume of parahippocampal (left hemisphere) | Freesurfer desikan white | 1.000000028 | 1.608554744 | 0.20471238 | 0.260651748 | 1.344570063 |
| MVPA | f.26804.2.0 | Volume of paracentral (left hemisphere) | Freesurfer desikan white | 2.295089134 | 1.722661625 | 0.133725883 | 0.191867572 | 1.650949875 |
| MVPA | f.26805.2.0 | Volume of parsopercularis (left hemisphere) | Freesurfer desikan white | 4.873474752 | 2.66486381 | 0.015923325 | 0.045693019 | 3.085809748 |
| MVPA | f.26806.2.0 | Volume of parsorbitalis (left hemisphere) | Freesurfer desikan white | 6.050183118 | 2.104531403 | 0.037957039 | 0.086384985 | 2.448941405 |
| MVPA | f.26807.2.0 | Volume of parstriangularis (left hemisphere) | Freesurfer desikan white | 5.220393267 | 2.913361687 | 0.006791435 | 0.026366749 | 3.635651557 |
| MVPA | f.26808.2.0 | Volume of pericalcarine (left hemisphere) | Freesurfer desikan white | 1.470920481 | 0.306449138 | 0.648646919 | 0.658626102 | 0.417599277 |
| MVPA | f.26809.2.0 | Volume of postcentral (left hemisphere) | Freesurfer desikan white | 6.36163408 | 2.018480665 | 0.051820437 | 0.105360833 | 2.250364314 |
| MVPA | f.26810.2.0 | Volume of posteriorcingulate (left hemisphere) | Freesurfer desikan white | 1.882111068 | 1.204101806 | 0.261006242 | 0.31320749 | 1.160889401 |
| MVPA | f.26811.2.0 | Volume of precentral (left hemisphere) | Freesurfer desikan white | 6.010436138 | 4.203734712 | 1.26E-04 | 0.002032024 | 6.198722908 |
| MVPA | f.26812.2.0 | Volume of precuneus (left hemisphere) | Freesurfer desikan white | 5.371049482 | 4.132344765 | 2.96E-04 | 0.003251519 | 5.728632903 |
| MVPA | f.26813.2.0 | Volume of rostralanteriorcingulate (left hemisphere) | Freesurfer desikan white | 1.465752553 | 8.05224277 | 0.002191329 | 0.013147972 | 4.331487731 |
| MVPA | f.26814.2.0 | Volume of rostralmiddlefrontal (left hemisphere) | Freesurfer desikan white | 6.672292089 | 2.986191317 | 0.002811452 | 0.015462985 | 4.169306201 |
| MVPA | f.26815.2.0 | Volume of superiorfrontal (left hemisphere) | Freesurfer desikan white | 6.28634508 | 2.912277301 | 0.005554219 | 0.025390964 | 3.673361927 |
| MVPA | f.26816.2.0 | Volume of superiorparietal (left hemisphere) | Freesurfer desikan white | 5.196897339 | 3.681892312 | 0.001150427 | 0.008436464 | 4.775191995 |
| MVPA | f.26817.2.0 | Volume of superiortemporal (left hemisphere) | Freesurfer desikan white | 1.983909379 | 1.835471204 | 0.151310388 | 0.203865498 | 1.590294827 |
| MVPA | f.26818.2.0 | Volume of supramarginal (left hemisphere) | Freesurfer desikan white | 6.344956579 | 3.605537747 | 6.06E-04 | 0.005000047 | 5.298308048 |
| MVPA | f.26819.2.0 | Volume of frontalpole (left hemisphere) | Freesurfer desikan white | 6.611466526 | 1.954595483 | 0.056090967 | 0.105771537 | 2.246473821 |
| MVPA | f.26820.2.0 | Volume of transversetemporal (left hemisphere) | Freesurfer desikan white | 5.632466949 | 2.820768607 | 0.007693932 | 0.02672629 | 3.622107554 |
| MVPA | f.26821.2.0 | Volume of insula (left hemisphere) | Freesurfer desikan white | 5.622524603 | 2.501154956 | 0.019378038 | 0.05115802 | 2.972836009 |
| MVPA | f.26890.2.0 | Volume of bankssts (right hemisphere) | Freesurfer desikan white | 3.345928273 | 0.873646922 | 0.429022494 | 0.479923468 | 0.734128629 |
| MVPA | f.26891.2.0 | Volume of caudalanteriorcingulate (right hemisphere) | Freesurfer desikan white | 1.000000009 | 3.704676156 | 0.054276793 | 0.105360833 | 2.250364314 |
| MVPA | f.26892.2.0 | Volume of caudalmiddlefrontal (right hemisphere) | Freesurfer desikan white | 2.113477655 | 3.642444401 | 0.01467601 | 0.04402803 | 3.122928808 |
| MVPA | f.26893.2.0 | Volume of cuneus (right hemisphere) | Freesurfer desikan white | 4.039183377 | 0.965818328 | 0.440376798 | 0.484414478 | 0.724814379 |
| MVPA | f.26894.2.0 | Volume of entorhinal (right hemisphere) | Freesurfer desikan white | 5.506629308 | 1.842900572 | 0.080788354 | 0.136096329 | 1.994392344 |
| MVPA | f.26895.2.0 | Volume of fusiform (right hemisphere) | Freesurfer desikan white | 5.46955454 | 2.001728372 | 0.067351534 | 0.120825596 | 2.113407127 |
| MVPA | f.26896.2.0 | Volume of inferiorparietal (right hemisphere) | Freesurfer desikan white | 4.433560074 | 2.100207682 | 0.052975481 | 0.105360833 | 2.250364314 |
| MVPA | f.26897.2.0 | Volume of inferiortemporal (right hemisphere) | Freesurfer desikan white | 4.535008442 | 0.649465284 | 0.578961942 | 0.597054503 | 0.515746875 |
| MVPA | f.26898.2.0 | Volume of isthmuscingulate (right hemisphere) | Freesurfer desikan white | 1.000000017 | 14.33043369 | 1.54E-04 | 0.002032024 | 6.198722908 |
| MVPA | f.26899.2.0 | Volume of lateraloccipital (right hemisphere) | Freesurfer desikan white | 5.321458638 | 2.009580262 | 0.05311665 | 0.105360833 | 2.250364314 |
| MVPA | f.26900.2.0 | Volume of lateralorbitofrontal (right hemisphere) | Freesurfer desikan white | 5.209181948 | 2.135084289 | 0.043335359 | 0.095337789 | 2.350329016 |
| MVPA | f.26901.2.0 | Volume of lingual (right hemisphere) | Freesurfer desikan white | 2.455194067 | 1.431004611 | 0.231250055 | 0.287971766 | 1.244892838 |
| MVPA | f.26902.2.0 | Volume of medialorbitofrontal (right hemisphere) | Freesurfer desikan white | 4.676054377 | 1.48045451 | 0.205361983 | 0.260651748 | 1.344570063 |
| MVPA | f.26903.2.0 | Volume of middletemporal (right hemisphere) | Freesurfer desikan white | 5.288526516 | 1.722910948 | 0.110409374 | 0.169465551 | 1.775105614 |
| MVPA | f.26904.2.0 | Volume of parahippocampal (right hemisphere) | Freesurfer desikan white | 4.317096253 | 1.816758727 | 0.084993835 | 0.136096329 | 1.994392344 |
| MVPA | f.26905.2.0 | Volume of paracentral (right hemisphere) | Freesurfer desikan white | 2.483849033 | 3.765088602 | 0.009681667 | 0.0319495 | 3.443598738 |
| MVPA | f.26906.2.0 | Volume of parsopercularis (right hemisphere) | Freesurfer desikan white | 5.154155933 | 2.867563571 | 0.00762875 | 0.02672629 | 3.622107554 |
| MVPA | f.26907.2.0 | Volume of parsorbitalis (right hemisphere) | Freesurfer desikan white | 5.960381179 | 1.52195534 | 0.149951171 | 0.203865498 | 1.590294827 |
| MVPA | f.26908.2.0 | Volume of parstriangularis (right hemisphere) | Freesurfer desikan white | 1.000000046 | 4.721604861 | 0.029799619 | 0.072843514 | 2.619441786 |
| MVPA | f.26909.2.0 | Volume of pericalcarine (right hemisphere) | Freesurfer desikan white | 4.066813568 | 0.938821681 | 0.448705777 | 0.485484939 | 0.722607013 |
| MVPA | f.26910.2.0 | Volume of postcentral (right hemisphere) | Freesurfer desikan white | 5.678702048 | 2.128730687 | 0.037281653 | 0.086384985 | 2.448941405 |
| MVPA | f.26911.2.0 | Volume of posteriorcingulate (right hemisphere) | Freesurfer desikan white | 2.442959066 | 2.221955011 | 0.084919833 | 0.136096329 | 1.994392344 |
| MVPA | f.26912.2.0 | Volume of precentral (right hemisphere) | Freesurfer desikan white | 5.582093386 | 4.008364633 | 3.88E-04 | 0.003660165 | 5.610247151 |
| MVPA | f.26913.2.0 | Volume of precuneus (right hemisphere) | Freesurfer desikan white | 5.335112586 | 3.414729417 | 0.002073534 | 0.013147972 | 4.331487731 |
| MVPA | f.26914.2.0 | Volume of rostralanteriorcingulate (right hemisphere) | Freesurfer desikan white | 1.000000011 | 7.58362379 | 0.005896248 | 0.025390964 | 3.673361927 |
| MVPA | f.26915.2.0 | Volume of rostralmiddlefrontal (right hemisphere) | Freesurfer desikan white | 6.464516376 | 1.363441575 | 0.17484446 | 0.230794687 | 1.466226766 |
| MVPA | f.26916.2.0 | Volume of superiorfrontal (right hemisphere) | Freesurfer desikan white | 5.648742186 | 2.459056582 | 0.016829173 | 0.046280225 | 3.073040506 |
| MVPA | f.26917.2.0 | Volume of superiorparietal (right hemisphere) | Freesurfer desikan white | 8.280025656 | 3.961752847 | 7.02E-05 | 0.001544118 | 6.473302332 |
| MVPA | f.26918.2.0 | Volume of superiortemporal (right hemisphere) | Freesurfer desikan white | 1.000000151 | 1.063466174 | 0.302440696 | 0.356447963 | 1.031567017 |
| MVPA | f.26919.2.0 | Volume of supramarginal (right hemisphere) | Freesurfer desikan white | 4.945310105 | 3.214944867 | 0.00377577 | 0.019169293 | 3.954445613 |
| MVPA | f.26920.2.0 | Volume of frontalpole (right hemisphere) | Freesurfer desikan white | 1.000000011 | 2.936678956 | 0.086606755 | 0.136096329 | 1.994392344 |
| MVPA | f.26921.2.0 | Volume of transversetemporal (right hemisphere) | Freesurfer desikan white | 1.000000099 | 23.35562378 | 1.70E-06 | 1.12E-04 | 9.094549142 |
| MVPA | f.26922.2.0 | Volume of insula (right hemisphere) | Freesurfer desikan white | 5.637070302 | 1.913403541 | 0.067735562 | 0.120825596 | 2.113407127 |
| VPA | f.26789.2.0 | Volume of bankssts (left hemisphere) | Freesurfer desikan white | 1.000000019 | 0.487049046 | 0.485256291 | 0.784113017 | 0.243202114 |
| VPA | f.26790.2.0 | Volume of caudalanteriorcingulate (left hemisphere) | Freesurfer desikan white | 1.000000012 | 6.379449248 | 0.011553836 | 0.238384037 | 1.433872306 |
| VPA | f.26791.2.0 | Volume of caudalmiddlefrontal (left hemisphere) | Freesurfer desikan white | 1.000000024 | 0.104786733 | 0.746163814 | 0.847709847 | 0.165216863 |
| VPA | f.26792.2.0 | Volume of cuneus (left hemisphere) | Freesurfer desikan white | 1.000000011 | 0.00172508 | 0.966871011 | 0.966871011 | 0.033690184 |
| VPA | f.26793.2.0 | Volume of entorhinal (left hemisphere) | Freesurfer desikan white | 1.000000158 | 0.001777385 | 0.966374128 | 0.966871011 | 0.033690184 |
| VPA | f.26794.2.0 | Volume of fusiform (left hemisphere) | Freesurfer desikan white | 1.046820238 | 0.271846989 | 0.600186797 | 0.784627302 | 0.242546449 |
| VPA | f.26795.2.0 | Volume of inferiorparietal (left hemisphere) | Freesurfer desikan white | 1.000000033 | 1.961698585 | 0.16134997 | 0.497308452 | 0.698544818 |
| VPA | f.26796.2.0 | Volume of inferiortemporal (left hemisphere) | Freesurfer desikan white | 1.000000012 | 0.314235749 | 0.575100268 | 0.784627302 | 0.242546449 |
| VPA | f.26797.2.0 | Volume of isthmuscingulate (left hemisphere) | Freesurfer desikan white | 2.866254154 | 3.450222486 | 0.014447517 | 0.238384037 | 1.433872306 |
| VPA | f.26798.2.0 | Volume of lateraloccipital (left hemisphere) | Freesurfer desikan white | 1.781217618 | 2.032496014 | 0.128339797 | 0.448568353 | 0.801694205 |
| VPA | f.26799.2.0 | Volume of lateralorbitofrontal (left hemisphere) | Freesurfer desikan white | 1.000000021 | 5.044400225 | 0.024718366 | 0.256145577 | 1.362009337 |
| VPA | f.26800.2.0 | Volume of lingual (left hemisphere) | Freesurfer desikan white | 1.000000014 | 1.574153281 | 0.209622061 | 0.548919692 | 0.599803129 |
| VPA | f.26801.2.0 | Volume of medialorbitofrontal (left hemisphere) | Freesurfer desikan white | 1.000000012 | 9.486554813 | 0.002073007 | 0.101171174 | 2.290941403 |
| VPA | f.26802.2.0 | Volume of middletemporal (left hemisphere) | Freesurfer desikan white | 1.00000004 | 8.770347296 | 0.003065793 | 0.101171174 | 2.290941403 |
| VPA | f.26803.2.0 | Volume of parahippocampal (left hemisphere) | Freesurfer desikan white | 1.877724114 | 1.069068471 | 0.408817701 | 0.744117645 | 0.295556131 |
| VPA | f.26804.2.0 | Volume of paracentral (left hemisphere) | Freesurfer desikan white | 1.004093525 | 1.157363709 | 0.280168143 | 0.634369061 | 0.455124379 |
| VPA | f.26805.2.0 | Volume of parsopercularis (left hemisphere) | Freesurfer desikan white | 1.000000135 | 0.382887498 | 0.536070398 | 0.784113017 | 0.243202114 |
| VPA | f.26806.2.0 | Volume of parsorbitalis (left hemisphere) | Freesurfer desikan white | 1.000000011 | 0.899394921 | 0.342957513 | 0.685915026 | 0.377001527 |
| VPA | f.26807.2.0 | Volume of parstriangularis (left hemisphere) | Freesurfer desikan white | 1.000000028 | 1.994904787 | 0.15784723 | 0.497308452 | 0.698544818 |
| VPA | f.26808.2.0 | Volume of pericalcarine (left hemisphere) | Freesurfer desikan white | 1.000000013 | 0.379327236 | 0.537971965 | 0.784113017 | 0.243202114 |
| VPA | f.26809.2.0 | Volume of postcentral (left hemisphere) | Freesurfer desikan white | 1.493022077 | 0.293641764 | 0.668506406 | 0.82824236 | 0.188449462 |
| VPA | f.26810.2.0 | Volume of posteriorcingulate (left hemisphere) | Freesurfer desikan white | 1.000000012 | 4.1581196 | 0.04145074 | 0.303972094 | 1.190819379 |
| VPA | f.26811.2.0 | Volume of precentral (left hemisphere) | Freesurfer desikan white | 3.631502953 | 1.817562949 | 0.129133314 | 0.448568353 | 0.801694205 |
| VPA | f.26812.2.0 | Volume of precuneus (left hemisphere) | Freesurfer desikan white | 1.00000003 | 0.311186116 | 0.576960888 | 0.784627302 | 0.242546449 |
| VPA | f.26813.2.0 | Volume of rostralanteriorcingulate (left hemisphere) | Freesurfer desikan white | 1.000000018 | 5.102869525 | 0.023898825 | 0.256145577 | 1.362009337 |
| VPA | f.26814.2.0 | Volume of rostralmiddlefrontal (left hemisphere) | Freesurfer desikan white | 1.000000043 | 0.095095273 | 0.757801227 | 0.847709847 | 0.165216863 |
| VPA | f.26815.2.0 | Volume of superiorfrontal (left hemisphere) | Freesurfer desikan white | 1.000000039 | 0.010303785 | 0.919149512 | 0.96143669 | 0.039326562 |
| VPA | f.26816.2.0 | Volume of superiorparietal (left hemisphere) | Freesurfer desikan white | 1.000000011 | 0.769782383 | 0.380296753 | 0.738223109 | 0.303509184 |
| VPA | f.26817.2.0 | Volume of superiortemporal (left hemisphere) | Freesurfer desikan white | 1.000000057 | 0.220915295 | 0.638349926 | 0.810213367 | 0.21045765 |
| VPA | f.26818.2.0 | Volume of supramarginal (left hemisphere) | Freesurfer desikan white | 1.000000054 | 0.028227165 | 0.866578463 | 0.922486751 | 0.080682265 |
| VPA | f.26819.2.0 | Volume of frontalpole (left hemisphere) | Freesurfer desikan white | 1.366734841 | 0.153482323 | 0.754842152 | 0.847709847 | 0.165216863 |
| VPA | f.26820.2.0 | Volume of transversetemporal (left hemisphere) | Freesurfer desikan white | 2.510132739 | 1.850265301 | 0.121315309 | 0.448568353 | 0.801694205 |
| VPA | f.26821.2.0 | Volume of insula (left hemisphere) | Freesurfer desikan white | 1.000000034 | 1.357803687 | 0.243934777 | 0.574989116 | 0.553404166 |
| VPA | f.26890.2.0 | Volume of bankssts (right hemisphere) | Freesurfer desikan white | 1.000000036 | 1.408254996 | 0.235362808 | 0.574989116 | 0.553404166 |
| VPA | f.26891.2.0 | Volume of caudalanteriorcingulate (right hemisphere) | Freesurfer desikan white | 1.399075808 | 0.883562974 | 0.299550356 | 0.634369061 | 0.455124379 |
| VPA | f.26892.2.0 | Volume of caudalmiddlefrontal (right hemisphere) | Freesurfer desikan white | 1.000000012 | 2.512162124 | 0.112989378 | 0.448568353 | 0.801694205 |
| VPA | f.26893.2.0 | Volume of cuneus (right hemisphere) | Freesurfer desikan white | 1.000000167 | 0.714485371 | 0.397970662 | 0.744117645 | 0.295556131 |
| VPA | f.26894.2.0 | Volume of entorhinal (right hemisphere) | Freesurfer desikan white | 1.370284739 | 0.194574959 | 0.793961621 | 0.873357783 | 0.135409976 |
| VPA | f.26895.2.0 | Volume of fusiform (right hemisphere) | Freesurfer desikan white | 8.379380395 | 1.759993977 | 0.06649399 | 0.426993881 | 0.850985595 |
| VPA | f.26896.2.0 | Volume of inferiorparietal (right hemisphere) | Freesurfer desikan white | 2.795594181 | 0.394107442 | 0.67765284 | 0.82824236 | 0.188449462 |
| VPA | f.26897.2.0 | Volume of inferiortemporal (right hemisphere) | Freesurfer desikan white | 1.000000152 | 0.00721673 | 0.932302245 | 0.96143669 | 0.039326562 |
| VPA | f.26898.2.0 | Volume of isthmuscingulate (right hemisphere) | Freesurfer desikan white | 2.891651829 | 2.89386032 | 0.030794002 | 0.256145577 | 1.362009337 |
| VPA | f.26899.2.0 | Volume of lateraloccipital (right hemisphere) | Freesurfer desikan white | 1.548516724 | 0.971150954 | 0.307572878 | 0.634369061 | 0.455124379 |
| VPA | f.26900.2.0 | Volume of lateralorbitofrontal (right hemisphere) | Freesurfer desikan white | 1.000000011 | 0.36363428 | 0.546503012 | 0.784113017 | 0.243202114 |
| VPA | f.26901.2.0 | Volume of lingual (right hemisphere) | Freesurfer desikan white | 1.000000012 | 0.108276954 | 0.742119112 | 0.847709847 | 0.165216863 |
| VPA | f.26902.2.0 | Volume of medialorbitofrontal (right hemisphere) | Freesurfer desikan white | 1.811405785 | 0.483768569 | 0.756186814 | 0.847709847 | 0.165216863 |
| VPA | f.26903.2.0 | Volume of middletemporal (right hemisphere) | Freesurfer desikan white | 1.000000028 | 0.587434184 | 0.443423767 | 0.770157069 | 0.2611608 |
| VPA | f.26904.2.0 | Volume of parahippocampal (right hemisphere) | Freesurfer desikan white | 1.000000021 | 0.035866846 | 0.849793506 | 0.919448712 | 0.083981015 |
| VPA | f.26905.2.0 | Volume of paracentral (right hemisphere) | Freesurfer desikan white | 1.950586935 | 1.570979073 | 0.201723621 | 0.548919692 | 0.599803129 |
| VPA | f.26906.2.0 | Volume of parsopercularis (right hemisphere) | Freesurfer desikan white | 1.000000251 | 0.459065322 | 0.498070382 | 0.784113017 | 0.243202114 |
| VPA | f.26907.2.0 | Volume of parsorbitalis (right hemisphere) | Freesurfer desikan white | 1.238701057 | 1.093862134 | 0.417156862 | 0.744117645 | 0.295556131 |
| VPA | f.26908.2.0 | Volume of parstriangularis (right hemisphere) | Freesurfer desikan white | 1.000000014 | 1.529256422 | 0.216241091 | 0.548919692 | 0.599803129 |
| VPA | f.26909.2.0 | Volume of pericalcarine (right hemisphere) | Freesurfer desikan white | 1.000000011 | 0.2656041 | 0.606302915 | 0.784627302 | 0.242546449 |
| VPA | f.26910.2.0 | Volume of postcentral (right hemisphere) | Freesurfer desikan white | 3.486247748 | 2.011646932 | 0.101490556 | 0.446558446 | 0.806184988 |
| VPA | f.26911.2.0 | Volume of posteriorcingulate (right hemisphere) | Freesurfer desikan white | 1.614091356 | 0.561006644 | 0.584093174 | 0.784627302 | 0.242546449 |
| VPA | f.26912.2.0 | Volume of precentral (right hemisphere) | Freesurfer desikan white | 5.262299618 | 1.700520106 | 0.097998037 | 0.446558446 | 0.806184988 |
| VPA | f.26913.2.0 | Volume of precuneus (right hemisphere) | Freesurfer desikan white | 1.000000038 | 0.405463948 | 0.524289613 | 0.784113017 | 0.243202114 |
| VPA | f.26914.2.0 | Volume of rostralanteriorcingulate (right hemisphere) | Freesurfer desikan white | 3.374127788 | 2.083147879 | 0.071165647 | 0.426993881 | 0.850985595 |
| VPA | f.26915.2.0 | Volume of rostralmiddlefrontal (right hemisphere) | Freesurfer desikan white | 1.000000011 | 2.699189129 | 0.100417725 | 0.446558446 | 0.806184988 |
| VPA | f.26916.2.0 | Volume of superiorfrontal (right hemisphere) | Freesurfer desikan white | 1.664489624 | 0.60539059 | 0.531678718 | 0.784113017 | 0.243202114 |
| VPA | f.26917.2.0 | Volume of superiorparietal (right hemisphere) | Freesurfer desikan white | 2.056534908 | 1.00333318 | 0.302068415 | 0.634369061 | 0.455124379 |
| VPA | f.26918.2.0 | Volume of superiortemporal (right hemisphere) | Freesurfer desikan white | 1.000000011 | 1.617289094 | 0.203487521 | 0.548919692 | 0.599803129 |
| VPA | f.26919.2.0 | Volume of supramarginal (right hemisphere) | Freesurfer desikan white | 1.396188923 | 0.435648471 | 0.500307721 | 0.784113017 | 0.243202114 |
| VPA | f.26920.2.0 | Volume of frontalpole (right hemisphere) | Freesurfer desikan white | 1.981992309 | 2.47656968 | 0.081221164 | 0.446558446 | 0.806184988 |
| VPA | f.26921.2.0 | Volume of transversetemporal (right hemisphere) | Freesurfer desikan white | 2.775384717 | 2.69895828 | 0.031047949 | 0.256145577 | 1.362009337 |
| VPA | f.26922.2.0 | Volume of insula (right hemisphere) | Freesurfer desikan white | 1.000000014 | 1.920955461 | 0.165769484 | 0.497308452 | 0.698544818 |
| LPA | f.26554.2.0 | Volume of Lateral-Ventricle (left hemisphere) | Freesurfer ASEG | 2.111709787 | 1.370574653 | 0.322203265 | 0.476269852 | 0.741770669 |
| LPA | f.26555.2.0 | Volume of Inf-Lat-Vent (left hemisphere) | Freesurfer ASEG | 5.508725795 | 2.406503413 | 0.019342747 | 0.123793582 | 2.089139765 |
| LPA | f.26556.2.0 | Volume of Cerebellum-White-Matter (left hemisphere) | Freesurfer ASEG | 1.000000147 | 3.790302063 | 0.051566696 | 0.226970042 | 1.482937242 |
| LPA | f.26557.2.0 | Volume of Cerebellum-Cortex (left hemisphere) | Freesurfer ASEG | 1.000000075 | 6.272243918 | 0.012273558 | 0.098188463 | 2.320866553 |
| LPA | f.26558.2.0 | Volume of Thalamus-Proper (left hemisphere) | Freesurfer ASEG | 1.000000074 | 1.28E-04 | 0.990979082 | 0.990979082 | 0.009061853 |
| LPA | f.26559.2.0 | Volume of Caudate (left hemisphere) | Freesurfer ASEG | 1.085077473 | 0.012274837 | 0.943568481 | 0.990979082 | 0.009061853 |
| LPA | f.26560.2.0 | Volume of Putamen (left hemisphere) | Freesurfer ASEG | 1.000000011 | 2.111725361 | 0.146192821 | 0.339526225 | 1.08020409 |
| LPA | f.26561.2.0 | Volume of Pallidum (left hemisphere) | Freesurfer ASEG | 1.000000021 | 1.38365386 | 0.239496136 | 0.414746785 | 0.880087102 |
| LPA | f.26523.2.0 | Volume of 3rd-Ventricle (whole brain) | Freesurfer ASEG | 1.735798893 | 1.462709724 | 0.25921674 | 0.414746785 | 0.880087102 |
| LPA | f.26524.2.0 | Volume of 4th-Ventricle (whole brain) | Freesurfer ASEG | 2.412167831 | 1.78826518 | 0.141643226 | 0.339526225 | 1.08020409 |
| LPA | f.26526.2.0 | Volume of Brain-Stem (whole brain) | Freesurfer ASEG | 1.393542671 | 1.562247155 | 0.148542723 | 0.339526225 | 1.08020409 |
| LPA | f.26562.2.0 | Volume of Hippocampus (left hemisphere) | Freesurfer ASEG | 1.507823591 | 4.304757583 | 0.030056236 | 0.160299923 | 1.830708701 |
| LPA | f.26563.2.0 | Volume of Amygdala (left hemisphere) | Freesurfer ASEG | 1.000001419 | 12.83228745 | 3.42E-04 | 0.005464328 | 5.209514081 |
| LPA | f.26564.2.0 | Volume of Accumbens-area (left hemisphere) | Freesurfer ASEG | 2.925635657 | 1.361055568 | 0.214963281 | 0.414746785 | 0.880087102 |
| LPA | f.26565.2.0 | Volume of VentralDC (left hemisphere) | Freesurfer ASEG | 1.000000025 | 0.026185859 | 0.87144965 | 0.969830116 | 0.030634361 |
| LPA | f.26585.2.0 | Volume of Lateral-Ventricle (right hemisphere) | Freesurfer ASEG | 2.905865302 | 1.628997909 | 0.188597041 | 0.402340355 | 0.910456895 |
| LPA | f.26586.2.0 | Volume of Inf-Lat-Vent (right hemisphere) | Freesurfer ASEG | 6.968090313 | 3.393825311 | 6.91E-04 | 0.007374561 | 4.909718969 |
| LPA | f.26587.2.0 | Volume of Cerebellum-White-Matter (right hemisphere) | Freesurfer ASEG | 1.320079827 | 0.272690537 | 0.611734216 | 0.752903651 | 0.283818013 |
| LPA | f.26588.2.0 | Volume of Cerebellum-Cortex (right hemisphere) | Freesurfer ASEG | 1.000000071 | 3.186323907 | 0.074274563 | 0.237678601 | 1.436835933 |
| LPA | f.26589.2.0 | Volume of Thalamus-Proper (right hemisphere) | Freesurfer ASEG | 2.186120168 | 1.032098956 | 0.460782847 | 0.589802044 | 0.527968317 |
| LPA | f.26590.2.0 | Volume of Caudate (right hemisphere) | Freesurfer ASEG | 1.797367787 | 1.180696812 | 0.327435523 | 0.476269852 | 0.741770669 |
| LPA | f.26591.2.0 | Volume of Putamen (right hemisphere) | Freesurfer ASEG | 1.000000021 | 0.854764771 | 0.355221031 | 0.494220565 | 0.704773374 |
| LPA | f.26592.2.0 | Volume of Pallidum (right hemisphere) | Freesurfer ASEG | 1.000000023 | 1.324951435 | 0.249721464 | 0.414746785 | 0.880087102 |
| LPA | f.26593.2.0 | Volume of Hippocampus (right hemisphere) | Freesurfer ASEG | 1.069133334 | 3.518712341 | 0.063722258 | 0.226970042 | 1.482937242 |
| LPA | f.26594.2.0 | Volume of Amygdala (right hemisphere) | Freesurfer ASEG | 1.000000077 | 23.74902574 | 1.28E-06 | 4.10E-05 | 10.10273292 |
| LPA | f.26595.2.0 | Volume of Accumbens-area (right hemisphere) | Freesurfer ASEG | 1.000000059 | 3.435271677 | 0.063835324 | 0.226970042 | 1.482937242 |
| LPA | f.26596.2.0 | Volume of VentralDC (right hemisphere) | Freesurfer ASEG | 1.000000054 | 0.002064124 | 0.963763823 | 0.990979082 | 0.009061853 |
| LPA | f.26531.2.0 | Volume of CC-Posterior (whole brain) | Freesurfer ASEG | 1.000000037 | 0.023212369 | 0.878908543 | 0.969830116 | 0.030634361 |
| LPA | f.26532.2.0 | Volume of CC-Mid-Posterior (whole brain) | Freesurfer ASEG | 1.529362326 | 0.293181646 | 0.714356287 | 0.846644489 | 0.166474403 |
| LPA | f.26533.2.0 | Volume of CC-Central (whole brain) | Freesurfer ASEG | 1.586047194 | 0.90194542 | 0.385551791 | 0.514069055 | 0.665397674 |
| LPA | f.26534.2.0 | Volume of CC-Mid-Anterior (whole brain) | Freesurfer ASEG | 1.000531336 | 2.584428128 | 0.107989649 | 0.314151708 | 1.157879264 |
| LPA | f.26535.2.0 | Volume of CC-Anterior (whole brain) | Freesurfer ASEG | 6.24277763 | 1.353639667 | 0.220404604 | 0.414746785 | 0.880087102 |
| MPA | f.26554.2.0 | Volume of Lateral-Ventricle (left hemisphere) | Freesurfer ASEG | 4.585423665 | 8.610641908 | 0 | 0 | Inf |
| MPA | f.26555.2.0 | Volume of Inf-Lat-Vent (left hemisphere) | Freesurfer ASEG | 7.06221019 | 3.065030776 | 0.001948428 | 0.005195807 | 5.259903247 |
| MPA | f.26556.2.0 | Volume of Cerebellum-White-Matter (left hemisphere) | Freesurfer ASEG | 3.28540269 | 3.770196398 | 0.004091269 | 0.008182539 | 4.805752819 |
| MPA | f.26557.2.0 | Volume of Cerebellum-Cortex (left hemisphere) | Freesurfer ASEG | 3.968923767 | 2.503886049 | 0.033681652 | 0.046861429 | 3.060560345 |
| MPA | f.26558.2.0 | Volume of Thalamus-Proper (left hemisphere) | Freesurfer ASEG | 2.887159358 | 1.300426683 | 0.291195432 | 0.291195432 | 1.23376065 |
| MPA | f.26559.2.0 | Volume of Caudate (left hemisphere) | Freesurfer ASEG | 1.000000873 | 14.23782077 | 1.62E-04 | 6.15E-04 | 7.394020731 |
| MPA | f.26560.2.0 | Volume of Putamen (left hemisphere) | Freesurfer ASEG | 1.000000027 | 17.54291161 | 2.85E-05 | 1.33E-04 | 8.925333322 |
| MPA | f.26561.2.0 | Volume of Pallidum (left hemisphere) | Freesurfer ASEG | 2.657565142 | 4.620567827 | 0.002303811 | 0.005670919 | 5.172404164 |
| MPA | f.26523.2.0 | Volume of 3rd-Ventricle (whole brain) | Freesurfer ASEG | 7.58447518 | 6.915585938 | 0 | 0 | Inf |
| MPA | f.26524.2.0 | Volume of 4th-Ventricle (whole brain) | Freesurfer ASEG | 4.98474865 | 1.603022147 | 0.142493795 | 0.157234532 | 1.850016752 |
| MPA | f.26526.2.0 | Volume of Brain-Stem (whole brain) | Freesurfer ASEG | 4.231155675 | 1.686990938 | 0.138539987 | 0.157234532 | 1.850016752 |
| MPA | f.26562.2.0 | Volume of Hippocampus (left hemisphere) | Freesurfer ASEG | 5.995802467 | 2.50179359 | 0.015223615 | 0.027064205 | 3.609543286 |
| MPA | f.26563.2.0 | Volume of Amygdala (left hemisphere) | Freesurfer ASEG | 6.442865864 | 3.707832094 | 4.95E-04 | 0.001584657 | 6.447387035 |
| MPA | f.26564.2.0 | Volume of Accumbens-area (left hemisphere) | Freesurfer ASEG | 4.646322917 | 2.591040943 | 0.017179236 | 0.02893345 | 3.542756908 |
| MPA | f.26565.2.0 | Volume of VentralDC (left hemisphere) | Freesurfer ASEG | 5.253281988 | 3.198005398 | 0.003531749 | 0.00807257 | 4.819283416 |
| MPA | f.26585.2.0 | Volume of Lateral-Ventricle (right hemisphere) | Freesurfer ASEG | 6.617325065 | 7.431220463 | 0 | 0 | Inf |
| MPA | f.26586.2.0 | Volume of Inf-Lat-Vent (right hemisphere) | Freesurfer ASEG | 3.231664692 | 2.437859631 | 0.042721451 | 0.054683457 | 2.90619404 |
| MPA | f.26587.2.0 | Volume of Cerebellum-White-Matter (right hemisphere) | Freesurfer ASEG | 2.736895381 | 2.562903869 | 0.041479522 | 0.054683457 | 2.90619404 |
| MPA | f.26588.2.0 | Volume of Cerebellum-Cortex (right hemisphere) | Freesurfer ASEG | 4.307978758 | 1.814568643 | 0.087479369 | 0.107666916 | 2.22871293 |
| MPA | f.26589.2.0 | Volume of Thalamus-Proper (right hemisphere) | Freesurfer ASEG | 2.467192772 | 2.967015579 | 0.028950379 | 0.042109642 | 3.167478539 |
| MPA | f.26590.2.0 | Volume of Caudate (right hemisphere) | Freesurfer ASEG | 1.549710429 | 11.85737002 | 1.00E-05 | 6.42E-05 | 9.653155978 |
| MPA | f.26591.2.0 | Volume of Putamen (right hemisphere) | Freesurfer ASEG | 1.000000018 | 17.50225928 | 2.91E-05 | 1.33E-04 | 8.925333322 |
| MPA | f.26592.2.0 | Volume of Pallidum (right hemisphere) | Freesurfer ASEG | 2.712139368 | 3.133711585 | 0.020150464 | 0.032240742 | 3.434524352 |
| MPA | f.26593.2.0 | Volume of Hippocampus (right hemisphere) | Freesurfer ASEG | 6.352891357 | 3.040944383 | 0.003803544 | 0.008114228 | 4.814136273 |
| MPA | f.26594.2.0 | Volume of Amygdala (right hemisphere) | Freesurfer ASEG | 5.801845907 | 5.639872432 | 2.24E-06 | 1.79E-05 | 10.93065017 |
| MPA | f.26595.2.0 | Volume of Accumbens-area (right hemisphere) | Freesurfer ASEG | 5.489593137 | 2.753768128 | 0.009160089 | 0.01724252 | 4.060376846 |
| MPA | f.26596.2.0 | Volume of VentralDC (right hemisphere) | Freesurfer ASEG | 5.516409089 | 3.641719954 | 9.38E-04 | 0.00272972 | 5.903556374 |
| MPA | f.26531.2.0 | Volume of CC-Posterior (whole brain) | Freesurfer ASEG | 1.64033543 | 1.85890237 | 0.163978609 | 0.174910516 | 1.743480772 |
| MPA | f.26532.2.0 | Volume of CC-Mid-Posterior (whole brain) | Freesurfer ASEG | 1.000000027 | 4.800186287 | 0.028470108 | 0.042109642 | 3.167478539 |
| MPA | f.26533.2.0 | Volume of CC-Central (whole brain) | Freesurfer ASEG | 2.696726695 | 1.743475443 | 0.124291412 | 0.14730834 | 1.915227339 |
| MPA | f.26534.2.0 | Volume of CC-Mid-Anterior (whole brain) | Freesurfer ASEG | 2.561495067 | 1.590975448 | 0.170188949 | 0.175678915 | 1.739097296 |
| MPA | f.26535.2.0 | Volume of CC-Anterior (whole brain) | Freesurfer ASEG | 1.000000023 | 14.11140735 | 1.73E-04 | 6.15E-04 | 7.394020731 |
| MVPA | f.26554.2.0 | Volume of Lateral-Ventricle (left hemisphere) | Freesurfer ASEG | 5.117092178 | 8.708621429 | 0 | 0 | Inf |
| MVPA | f.26555.2.0 | Volume of Inf-Lat-Vent (left hemisphere) | Freesurfer ASEG | 5.76877237 | 2.986954512 | 0.005381634 | 0.010763268 | 4.531616034 |
| MVPA | f.26556.2.0 | Volume of Cerebellum-White-Matter (left hemisphere) | Freesurfer ASEG | 2.99745976 | 4.472833259 | 0.001793716 | 0.004415302 | 5.422679038 |
| MVPA | f.26557.2.0 | Volume of Cerebellum-Cortex (left hemisphere) | Freesurfer ASEG | 3.322343398 | 2.902264164 | 0.021786232 | 0.034857971 | 3.356473444 |
| MVPA | f.26558.2.0 | Volume of Thalamus-Proper (left hemisphere) | Freesurfer ASEG | 2.405792033 | 1.133481946 | 0.336408604 | 0.336408604 | 1.089428774 |
| MVPA | f.26559.2.0 | Volume of Caudate (left hemisphere) | Freesurfer ASEG | 1.000000031 | 15.61027343 | 7.82E-05 | 2.78E-04 | 8.188050617 |
| MVPA | f.26560.2.0 | Volume of Putamen (left hemisphere) | Freesurfer ASEG | 1.000000092 | 17.75147222 | 2.54E-05 | 1.16E-04 | 9.059834672 |
| MVPA | f.26561.2.0 | Volume of Pallidum (left hemisphere) | Freesurfer ASEG | 2.513181094 | 5.420679186 | 8.31E-04 | 0.002660321 | 5.929308474 |
| MVPA | f.26523.2.0 | Volume of 3rd-Ventricle (whole brain) | Freesurfer ASEG | 6.603795894 | 7.485389391 | 0 | 0 | Inf |
| MVPA | f.26524.2.0 | Volume of 4th-Ventricle (whole brain) | Freesurfer ASEG | 3.820889194 | 1.464460677 | 0.217228221 | 0.224235583 | 1.495058069 |
| MVPA | f.26526.2.0 | Volume of Brain-Stem (whole brain) | Freesurfer ASEG | 2.69530144 | 1.866520806 | 0.12473934 | 0.133708312 | 2.012094625 |
| MVPA | f.26562.2.0 | Volume of Hippocampus (left hemisphere) | Freesurfer ASEG | 5.820395813 | 2.231135792 | 0.028984402 | 0.042627173 | 3.15526336 |
| MVPA | f.26563.2.0 | Volume of Amygdala (left hemisphere) | Freesurfer ASEG | 6.01499409 | 3.31174203 | 0.001636014 | 0.004362704 | 5.434663262 |
| MVPA | f.26564.2.0 | Volume of Accumbens-area (left hemisphere) | Freesurfer ASEG | 4.371268009 | 2.382506814 | 0.030638281 | 0.042627173 | 3.15526336 |
| MVPA | f.26565.2.0 | Volume of VentralDC (left hemisphere) | Freesurfer ASEG | 5.323382288 | 3.224745254 | 0.003190114 | 0.006805576 | 4.990012937 |
| MVPA | f.26585.2.0 | Volume of Lateral-Ventricle (right hemisphere) | Freesurfer ASEG | 6.211483428 | 7.996676189 | 0 | 0 | Inf |
| MVPA | f.26586.2.0 | Volume of Inf-Lat-Vent (right hemisphere) | Freesurfer ASEG | 3.17949829 | 2.379617624 | 0.048217439 | 0.061718323 | 2.785174431 |
| MVPA | f.26587.2.0 | Volume of Cerebellum-White-Matter (right hemisphere) | Freesurfer ASEG | 2.457960598 | 2.672461913 | 0.042033068 | 0.056044091 | 2.881616555 |
| MVPA | f.26588.2.0 | Volume of Cerebellum-Cortex (right hemisphere) | Freesurfer ASEG | 4.326833072 | 1.948870738 | 0.065824957 | 0.081015332 | 2.513116864 |
| MVPA | f.26589.2.0 | Volume of Thalamus-Proper (right hemisphere) | Freesurfer ASEG | 2.330419116 | 3.464997193 | 0.019888373 | 0.033496207 | 3.396323069 |
| MVPA | f.26590.2.0 | Volume of Caudate (right hemisphere) | Freesurfer ASEG | 1.122959725 | 19.85930557 | 2.40E-06 | 1.92E-05 | 10.86264179 |
| MVPA | f.26591.2.0 | Volume of Putamen (right hemisphere) | Freesurfer ASEG | 1.000000095 | 17.9912251 | 2.24E-05 | 1.16E-04 | 9.059834672 |
| MVPA | f.26592.2.0 | Volume of Pallidum (right hemisphere) | Freesurfer ASEG | 2.413534185 | 3.435970852 | 0.015614947 | 0.027759906 | 3.584162512 |
| MVPA | f.26593.2.0 | Volume of Hippocampus (right hemisphere) | Freesurfer ASEG | 6.878286555 | 2.992962055 | 0.003104014 | 0.006805576 | 4.990012937 |
| MVPA | f.26594.2.0 | Volume of Amygdala (right hemisphere) | Freesurfer ASEG | 5.60162086 | 5.465271242 | 4.64E-06 | 2.97E-05 | 10.42514095 |
| MVPA | f.26595.2.0 | Volume of Accumbens-area (right hemisphere) | Freesurfer ASEG | 5.802201445 | 2.205687592 | 0.029411981 | 0.042627173 | 3.15526336 |
| MVPA | f.26596.2.0 | Volume of VentralDC (right hemisphere) | Freesurfer ASEG | 5.458588188 | 3.554047718 | 0.001258803 | 0.003661973 | 5.609753126 |
| MVPA | f.26531.2.0 | Volume of CC-Posterior (whole brain) | Freesurfer ASEG | 1.825127474 | 2.245781022 | 0.112780408 | 0.128891895 | 2.048781249 |
| MVPA | f.26532.2.0 | Volume of CC-Mid-Posterior (whole brain) | Freesurfer ASEG | 1.000000031 | 6.311202329 | 0.012006839 | 0.022601108 | 3.789756327 |
| MVPA | f.26533.2.0 | Volume of CC-Central (whole brain) | Freesurfer ASEG | 2.618750893 | 2.0159174 | 0.087812793 | 0.104074421 | 2.262649049 |
| MVPA | f.26534.2.0 | Volume of CC-Mid-Anterior (whole brain) | Freesurfer ASEG | 2.500637918 | 1.833983247 | 0.125351543 | 0.133708312 | 2.012094625 |
| MVPA | f.26535.2.0 | Volume of CC-Anterior (whole brain) | Freesurfer ASEG | 1.000000017 | 16.28771704 | 5.46E-05 | 2.18E-04 | 8.428937646 |
| VPA | f.26554.2.0 | Volume of Lateral-Ventricle (left hemisphere) | Freesurfer ASEG | 3.534487997 | 12.83051726 | 0 | 0 | Inf |
| VPA | f.26555.2.0 | Volume of Inf-Lat-Vent (left hemisphere) | Freesurfer ASEG | 4.596591098 | 2.797555958 | 0.016401404 | 0.041155086 | 3.190407763 |
| VPA | f.26556.2.0 | Volume of Cerebellum-White-Matter (left hemisphere) | Freesurfer ASEG | 1.000000051 | 0.454416654 | 0.500254267 | 0.602451907 | 0.506747439 |
| VPA | f.26557.2.0 | Volume of Cerebellum-Cortex (left hemisphere) | Freesurfer ASEG | 1.69372659 | 3.640122359 | 0.025417282 | 0.054223534 | 2.914640252 |
| VPA | f.26558.2.0 | Volume of Thalamus-Proper (left hemisphere) | Freesurfer ASEG | 1.000000031 | 1.514024608 | 0.218542956 | 0.317880663 | 1.14607924 |
| VPA | f.26559.2.0 | Volume of Caudate (left hemisphere) | Freesurfer ASEG | 6.143523064 | 3.259766037 | 0.001566661 | 0.008355524 | 4.784832452 |
| VPA | f.26560.2.0 | Volume of Putamen (left hemisphere) | Freesurfer ASEG | 1.818245951 | 3.188903895 | 0.041741865 | 0.083483729 | 2.483103528 |
| VPA | f.26561.2.0 | Volume of Pallidum (left hemisphere) | Freesurfer ASEG | 1.000000025 | 6.540542396 | 0.010552814 | 0.037521118 | 3.282851359 |
| VPA | f.26523.2.0 | Volume of 3rd-Ventricle (whole brain) | Freesurfer ASEG | 3.593693855 | 11.2852097 | 0 | 0 | Inf |
| VPA | f.26524.2.0 | Volume of 4th-Ventricle (whole brain) | Freesurfer ASEG | 2.829821195 | 2.125374846 | 0.087056755 | 0.154767564 | 1.865830875 |
| VPA | f.26526.2.0 | Volume of Brain-Stem (whole brain) | Freesurfer ASEG | 1.000000011 | 0.114327477 | 0.735274872 | 0.784293197 | 0.242972352 |
| VPA | f.26562.2.0 | Volume of Hippocampus (left hemisphere) | Freesurfer ASEG | 1.000000012 | 0.122583066 | 0.726254493 | 0.784293197 | 0.242972352 |
| VPA | f.26563.2.0 | Volume of Amygdala (left hemisphere) | Freesurfer ASEG | 1.000000032 | 6.078167227 | 0.013695979 | 0.039842847 | 3.22281239 |
| VPA | f.26564.2.0 | Volume of Accumbens-area (left hemisphere) | Freesurfer ASEG | 1.000000021 | 0.437545211 | 0.508318797 | 0.602451907 | 0.506747439 |
| VPA | f.26565.2.0 | Volume of VentralDC (left hemisphere) | Freesurfer ASEG | 1.000000011 | 0.912420069 | 0.339486708 | 0.472329333 | 0.750078798 |
| VPA | f.26585.2.0 | Volume of Lateral-Ventricle (right hemisphere) | Freesurfer ASEG | 3.732089379 | 10.86803671 | 0 | 0 | Inf |
| VPA | f.26586.2.0 | Volume of Inf-Lat-Vent (right hemisphere) | Freesurfer ASEG | 3.275910077 | 3.556964157 | 0.0067316 | 0.0269264 | 3.614648071 |
| VPA | f.26587.2.0 | Volume of Cerebellum-White-Matter (right hemisphere) | Freesurfer ASEG | 1.000000027 | 0.007732863 | 0.929928821 | 0.938114247 | 0.063883539 |
| VPA | f.26588.2.0 | Volume of Cerebellum-Cortex (right hemisphere) | Freesurfer ASEG | 1.094649978 | 3.380786859 | 0.049199585 | 0.092610984 | 2.379347522 |
| VPA | f.26589.2.0 | Volume of Thalamus-Proper (right hemisphere) | Freesurfer ASEG | 1.000000038 | 2.348750613 | 0.12540199 | 0.211203352 | 1.554933858 |
| VPA | f.26590.2.0 | Volume of Caudate (right hemisphere) | Freesurfer ASEG | 2.93629533 | 6.345906092 | 1.28E-04 | 8.19E-04 | 7.1068985 |
| VPA | f.26591.2.0 | Volume of Putamen (right hemisphere) | Freesurfer ASEG | 1.619480905 | 3.848527693 | 0.021316981 | 0.048724528 | 3.02157273 |
| VPA | f.26592.2.0 | Volume of Pallidum (right hemisphere) | Freesurfer ASEG | 1.0045326 | 2.081931899 | 0.147554574 | 0.236087318 | 1.44355355 |
| VPA | f.26593.2.0 | Volume of Hippocampus (right hemisphere) | Freesurfer ASEG | 1.000000012 | 0.131185995 | 0.717210601 | 0.784293197 | 0.242972352 |
| VPA | f.26594.2.0 | Volume of Amygdala (right hemisphere) | Freesurfer ASEG | 1.679691393 | 4.08837492 | 0.016719254 | 0.041155086 | 3.190407763 |
| VPA | f.26595.2.0 | Volume of Accumbens-area (right hemisphere) | Freesurfer ASEG | 1.000000014 | 0.006028298 | 0.938114247 | 0.938114247 | 0.063883539 |
| VPA | f.26596.2.0 | Volume of VentralDC (right hemisphere) | Freesurfer ASEG | 1.387286995 | 0.425239215 | 0.502957689 | 0.602451907 | 0.506747439 |
| VPA | f.26531.2.0 | Volume of CC-Posterior (whole brain) | Freesurfer ASEG | 1.171783624 | 1.313298512 | 0.202658862 | 0.308813504 | 1.175017732 |
| VPA | f.26532.2.0 | Volume of CC-Mid-Posterior (whole brain) | Freesurfer ASEG | 2.023304723 | 5.299545014 | 0.002802705 | 0.012812368 | 4.357344331 |
| VPA | f.26533.2.0 | Volume of CC-Central (whole brain) | Freesurfer ASEG | 2.002637444 | 3.899084688 | 0.012984359 | 0.039842847 | 3.22281239 |
| VPA | f.26534.2.0 | Volume of CC-Mid-Anterior (whole brain) | Freesurfer ASEG | 2.003219204 | 1.124244891 | 0.425485643 | 0.56731419 | 0.566842002 |
| VPA | f.26535.2.0 | Volume of CC-Anterior (whole brain) | Freesurfer ASEG | 1.000000014 | 14.94260546 | 1.11E-04 | 8.19E-04 | 7.1068985 |

Note: *P*-FDR was calculated by FDR correction (α=0.05) of *P*. nlogP-FDR was calculated by -log_10_(*P*-FDR). Models were adjusted for age, sex, ethnicity, educational attainment, Townsend deprivation index, smoking status, alcohol consumption, and dietary pattern. EDF (effective degrees of freedom) estimated from generalized additive models were used as a proxy for the degree of non-linearity. FreeSurfer aparc (category ID 192) and the aseg (category ID 190) atlas corresponding to 66 cortical regions and 32 subcortical regions were used.
